# Screening for Toxic Stress Response and Buffering Factors: A Case-Based, Trauma-Informed Approach to Health Equity

**DOI:** 10.15766/mep_2374-8265.11224

**Published:** 2022-03-04

**Authors:** Adwoa Osei, Camila Garcia Paz, Mallory Stuparich, Rebeca Racataian-Gavan, Laurel Nelms, Yasmine Suliman, Amanda Smith, Moazzum Bajwa

**Affiliations:** 1 Assistant Professor, Department of Pediatrics, University of California, Riverside, School of Medicine; 2 Resident Physician, Department of Pediatrics, UCLA Health, University of California, Los Angeles; 3 Assistant Professor, Department of Obstetrics and Gynecology, University of California, Riverside, School of Medicine; 4 Fourth-Year Medical Student, University of California, Riverside, School of Medicine; 5 Second-Year Medical Student, University of California, Riverside, School of Medicine; 6 Director of Medical Student Support and Wellness, University of California, Riverside, School of Medicine; 7 Assistant Professor, Department of Family Medicine, University of California, Riverside, School of Medicine

**Keywords:** Trauma-Informed Care, Toxic Stress Response, Resiliency, Adverse Childhood Experience, Social Justice, Cultural Sensitivity, Case-Based Learning, Diversity, Inclusion, Health Equity, Anti-racism

## Abstract

**Introduction:**

Exposure to adverse childhood experiences (ACEs) can lead to a toxic stress response with impacts on health that affect health equity. As part of our Health Equity, Social Justice, and Anti-racism curriculum, our aim was to introduce second-year medical students to a case-based method using a template-based screening and application of toxic stress, buffering factors, and resiliency-fostering tools to address health disparities and inequities with a trauma-informed care approach.

**Methods:**

We developed an asynchronous e-learning module that demonstrated the impact of ACEs by introducing students to screening for toxic stress response and buffering factors on health, their role as health equity determinants, and the use of brief in-clinic resilience-fostering tools in patient care. This was followed by a synchronous, facilitated, small-group, virtual discussion of a clinical case. Pre- and postworkshop surveys assessed changes in knowledge, skills, and attitudes. A 3-month follow-up survey assessed students’ behavioral changes.

**Results:**

Sixty-four students completed the learning module. Paired *t*-test analysis showed a statistically significant increase in students’ knowledge, skills, and attitudes regarding the Educational Objectives, with a survey response rate of 98%. Three months after the workshop, a third of students were applying these concepts, with a survey response rate of 87%.

**Discussion:**

Implementing this case-based curriculum in trauma-informed patient care helped increase opportunities for equitable health in patient encounters by providing students with the skills to screen for toxic stress, buffering, and brief in-clinic resiliency-fostering tools. Such skills will become even more impactful as we emerge from the COVID-19 pandemic.

## Educational Objectives

By the end of this workshop, learners will be able to:

1. Define adverse childhood experiences (ACEs) and describe their prevalence and impacts on physical, mental, and social health.
2. Describe ACEs as determinants of health equity.
3. Demonstrate ACEs and toxic stress screening in patient care.
4. Identify buffering factors in patients exposed to ACEs.
5. Apply three brief in-clinic trauma-informed resilience-fostering techniques for patients exposed to ACEs.

## Introduction

Adverse childhood experiences (ACEs) are traumatic experiences that occur during the first 18 years of life.^1^ These experiences play critical roles in behavioral, social, and neurobiological changes, with persistent and long-term consequences on health and life opportunities across the life span, and may even be transmitted across generations.^2^ Earlier definitions highlighted dysfunction in the relationship between a child and a caregiver such as neglect, abuse, and household instability. Recent definitions include experiences like racism, community violence, life-altering medical conditions, and commercial sexual exploitation.^3^ These experiences can lead to dysregulated neurologic, immune, endocrine, autonomic, inflammatory, and metabolic processes that otherwise serve as reversible protective mechanisms. This has been described as the toxic stress response.^4^ Both the duration and the cumulative dose of ACEs increase the risk of a toxic stress response and have a direct correlation to its adverse effect on multiple domains of the life span.^2^ In children, the effects include impaired growth, delayed development, and learning problems. In adolescents and adults, ACEs have been associated with risk-taking behaviors, mood disorders, chronic health conditions, and an overall shortened life span.^1^ Four or more ACEs increase the risk of a toxic stress response and have been linked to the top 10 leading causes of death in the United States.^5^

According to the National Center for Chronic Disease Prevention and Health Promotion, health equity is the opportunity to attain one's “full health potential” without being disadvantaged by “social position or other socially determined circumstances.”^6^ Health inequities are reflected in disparities in quality of life; rates of morbidity, disability, and mortality; and access to health care.^7^ Cumulative and prolonged ACEs are associated with poor social outcomes, such as a shortened life span, decreased worker performance, unemployment, and disability, and have been described as “social determinants of health that affect individuals and communities.”^8^ With resultant toxic stress, ACEs are a major contributor to health disparities^9^ and may operate sequentially with other social determinants of health. Due to lack of equity, unfairly disadvantaged community environments with high rates of poverty, poor housing conditions, violence, and homelessness have higher rates of ACEs such as abuse, neglect, parental substance abuse, and incarceration.^10^ Vulnerable and historically disadvantaged populations tend to have a disproportionately higher prevalence of ACEs and ACE scores because they are burdened by experiences of racism and inequities in quality housing, education, health care, historical injustice, and cultural complexity.^11–15^ The American Academy of Pediatrics proposes that “many adult diseases should be viewed as developmental disorders that begin early in life and that persistent health disparities associated with poverty, discrimination, or maltreatment could be reduced by the alleviation of toxic stress in childhood.”^4^ Attention to these aspects of a person's lived experience provides opportunities for equitable and optimal health care.

Trauma-informed care (TIC) includes recognition of ACEs, such that an individual gains not only a clear understanding of the health sequelae of a toxic stress response but also the ability to respond appropriately in a culturally, historically, and gender-sensitive way.^16^ Best practices on optimal screening timing for ACEs are still evolving. However, in the context of signs and symptoms of a toxic stress response, asking the question “What has happened to you?” provides opportunities for cultural, historical, and gender contexts to be considered in health care optimization for people exposed to prolonged adversities. A toxic stress response may present as dysregulated behaviors, a sense of feeling unsafe, and a loss of hope. Approaches that promote restoring hope and safety, routines, and regulation foster resilience and enhance protective factors. To identify protective factors such as hope and social and emotional support against poor physical and mental health and unemployment, TIC resilience and healing‐centered approaches ask, “What is strong with you?”^17,18^ In children, the risk of toxic stress can be reduced when there is a safe and nurturing buffering relationship between a caregiver and a child.^19^ Children with high ACE scores and a toxic stress response have a higher risk of adverse and traumatic experiences as adults, perpetuating intergenerational trauma.^20^ By considering contextual roles of social determinants of health, culture, history, gender, and the impact of ACEs, TIC increases opportunities for compassionate, relationship-centered, whole care, as well as for the identification of protective factors to support resiliency.

It is critical that health care providers can navigate these topics during patient encounters to maximize opportunities for reinforcing resiliency and positive coping strategies as a means of maximizing opportunities for equity in health.^21^ Therefore, it is imperative that medical education curricula include and make standard roles of these topics in health disparities and inequities. Current *MedEdPORTAL* publications feature introductory ACEs lectures, small-group case discussions, and presentations on trauma-informed physical examination techniques for medical students and residents.^22–25^ Particularly lacking are a consistent screening template for toxic stress response and buffering factors, instruction on the application of brief point-of-care resiliency-fostering tools, and follow-up with learners to assess the implementation of skills in clinical practice.

In our case-based approach, consisting of a virtual didactic e-module and facilitated small-group dialogue, we aimed to address health disparities and inequities using a trauma-informed approach. Our objective was to equip medical students with easy-to-use screening templates for toxic stress, buffering factors, and resiliency-fostering tools for use in point-of-care interactions with patients to add to the health equity discussion in medical education curricula using a trauma-informed approach.

## Methods

We incorporated the workshop as a required course in the Health Equity, Social Justice, and Anti-racism (HESJAR) curriculum thread, as part of the Longitudinal Ambulatory Care Experience (LACE) for second-year medical students. LACE consisted of both longitudinal integrated clerkship and curriculum threads. During the LACE clerkship, students were paired with community primary care physicians for 1 half-day of clinic, every other week, during the first 2 years of medical school. The students participated in patient care under direct supervision of their community preceptors, with a minimum requirement of 16 patient encounters per year. Each LACE clerkship site followed curriculum guidelines to ensure comparable learning experiences across different sites. Our second-year HESJAR curriculum focused on communication, advocacy, and TIC. We introduced the workshop 6 months before the onset of traditional third-year clerkship rotations and sent a 3-month follow-up survey to reinforce concepts and practice during the third- and fourth-year clerkship years. Although our curriculum allowed for early patient contact, the workshop could be completed by students with no prior direct patient care experience.

### Materials

Materials required for the workshop included a learning management system to host an e-learning module, WiFi accessibility for synchronous and asynchronous learning, a virtual meeting platform with breakout room features, closed captioning, an online survey platform, PowerPoint slides, (Appendix A), a facilitator guide (Appendix B) and slides (Appendix C), a student handout (Appendix D), and screening templates, which were used with permission from the American Academy of Pediatrics Trauma and Resiliency Project.^17,18^ Our workshop was exempted from review by the Institutional Review Board at the University of California, Riverside, School of Medicine.

### Session I: E-learning Module

A 30-minute e-learning module was created using PowerPoint slides (Appendix A) and hosted on a learning management system. Students viewed the slides independently and completed the module asynchronously in 1 week. At the end of the module, students were encouraged to move away from a perspective of “What's wrong with you?” toward a perspective of “What happened to you?” and an approach of “What's strong with you?” in patient care. We tracked students’ completion of the module in a Google Document. To ensure the module was easy to navigate and relevant to the learning outcomes, faculty from the Office of Medical Education, OB/GYN, and social sciences reviewed and completed the module prior to students’ access.

### Session II: Small-Group Case Discussion

Facilitators were trained and oriented through an online session 1 week prior to the sessions with students. Facilitators completed and discussed the clinical case, guides, and resources (Appendices B and C) to ensure consistency among small groups. Students were given access to electronic copies of a clinical case study with questions and screening templates (Appendix D) for preview 24 hours prior to small-group discussions. The case highlighted a 12-year-old male and his mother with signs and symptoms of toxic stress response to cumulative ACEs exposure. Faculty from pediatrics and family medicine and fourth-year medical students facilitated sessions. Students gathered synchronously for a live virtual discussion of the case. Designated faculty presented a 10-minute narrative overview of topics covered by the e-module (Appendix A) to students prior to small-group breakouts. Students were randomly assigned into facilitated small groups for 30 minutes. Using the case as a guide, students worked virtually on a clinical case to identify and score cumulative ACEs, screen for signs and symptoms of toxic stress, discuss how ACEs could be determinants of health equity, screen for buffering factors, and discuss how to use the given template to foster resiliency tools in point-of-care interactions. They returned to the large group for 20 minutes to report on their findings and explore how they would incorporate these skills into patient care.

### Evaluation

Using Kirkpatrick's model of learning,^26^ we designed our pre- and postsurveys to reflect the degree to which students self-rated acquired knowledge, skills, and attitudes reflected in the Educational Objectives (Level 2, Learning; Appendix E) by asking questions such as “Describe one way you plan to promote buffering and protective factors in patient care.” To assess self-reported behavioral change in the application of skills learned, a 3-month follow-up survey asked LACE students questions such as “In the last 3 months, have you screened for ACEs and toxic stress in patients?” (Level 3, Behavior; Appendix E). We piloted the surveys with a group of fourth-year medical students as well as with faculty from social sciences, pediatrics, and family medicine and revised the surveys for usability. Data were collected anonymously from surveys administered using the Qualtrics Online Surveys platform. The Office of Assessment and Evaluation at our institution used Microsoft Excel to calculate a paired *t* test to analyze pre- and postsurvey data for statistical significance.

Three months after the initial workshop, we implemented it again at a conference for Building the Next Generation of Academic Physicians (BNGAP), with participants of different learning levels and other health professions, such as social work, nursing, resident physicians, first- to fourth-year medical students, and nonclinical medical educators. This conference implementation helped us to evaluate the generalizability of the workshop. The e-module was presented live as a 20-minute introductory PowerPoint didactic to participants and was followed by a large-group case discussion session. Based on feedback from students after the first implementation of the workshop, the case discussion session was 1 hour long.

## Results

Sixty-five second-year medical students completed both sessions, with a response rate of 98% (*n* = 64) on pre- and postsurveys. After 3 months, the response rate was 87% (*n* = 57). Overall, there was a statistically significant increase in students’ self-rated knowledge and skills across all five Educational Objectives postworkshop (Table 1). Additionally, the percentage of students who responded *agree* to the Educational Objectives increased in the postworkshop survey compared to the preworkshop survey (Figure). Students also reported implementing concepts and skills in patient care 3 months after the workshop.

**Table 1.**
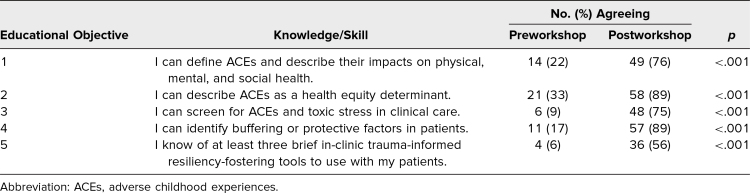
Students’ Self-Reported Knowledge and Skills Pre- and Postworkshop (*N* = 64)

**Figure.**
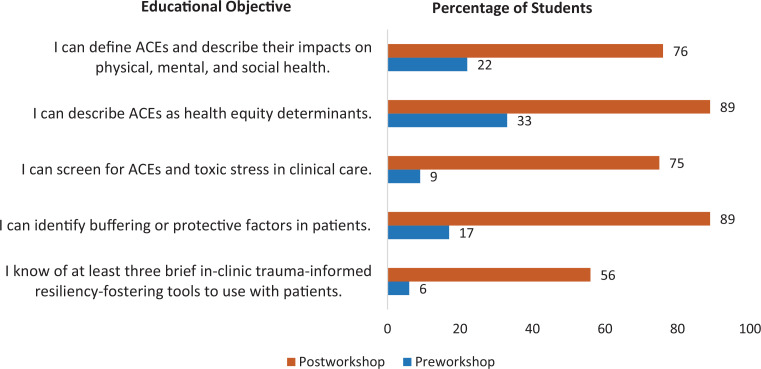
Percentage of students who responded *agree* to the Educational Objectives on pre- and postworkshop surveys (*N* = 64). Abbreviation: ACEs, adverse childhood experiences.

After 3 months, 19% of students had screened for ACEs and toxic stress in patient care, 43% had identified buffering factors in patients in clinical care, 42% had used resiliency-promoting tools in clinical patient care, and 26% reported using all TIC concepts (i.e., incorporating a patient's history of ACEs, toxic stress, and buffering system) as part of their clinical encounter. Overall, roughly one-fifth to one-third of the students had put these concepts into practice.

Students commented favorably on the practical use of screening tools and the interactive nature and conciseness of the case-based sessions (Table 2). They indicated that they planned to incorporate these skills into patient care, such as through comments referencing the use of templates and “placing strengths in summary reports.” Students suggested increasing the time for small-group discussions, adding more clinical cases, and including live patient interviews in future implementations of the workshop. The most common barriers reported by students for implementation 3 months after the workshop were (1) lack of patient volume due to the COVID-19 pandemic and (2) TIC not routinely being practiced in attending clinics.

**Table 2.**
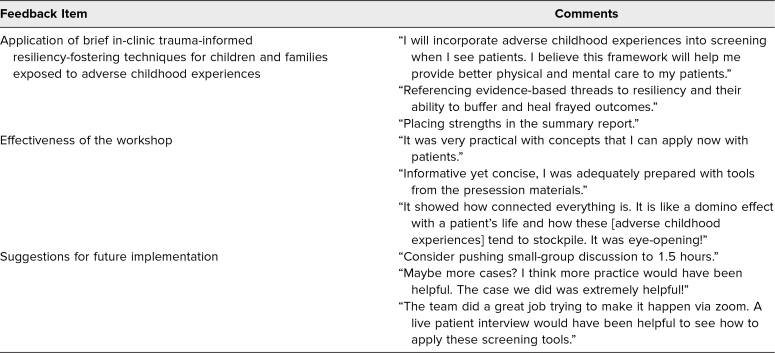
Summary of Students’ Qualitative Feedback on the Postworkshop Survey

Data from BNGAP conference attendees were not included in the quantitative analysis due to small sample size (*n* = 14). After completing the workshop, participants at the BNGAP conference reported that “the screening tools will be a game changer in the assessment of social determinants of health and their impact on health outcomes” and that “non-clinicians would be able to explain its use to improve patient outcomes.”

## Discussion

To address a trauma-informed approach to health equity, we developed and implemented a case-based workshop that incorporated the screening of toxic stress, identification of resilience factors, and application of TIC in patient care for second-year medical students. The workshop required students to self-learn the material in advance of the small-group, case-based session. The introduction of this workshop was received well by students. They loved the practicality, interactive nature, and conciseness of the workshop. They also found that the workshop increased their knowledge and confidence regarding critical concepts on ACEs, toxic stress, and buffering factors, as well as empowering them to utilize TIC skills in clinical care. Our work builds upon previously developed and published medical schools’ modules by connecting these critical concepts to health equity, the use of practical tools and templates, and follow-up data on clinical behaviors.

Overall, there was a high utilization of tools and templates, with approximately a third of the students reporting using all concepts of TIC described in the workshop in practice 3 months after the workshop. Though the longitudinal nature of students’ clinical experience through LACE allowed for an almost immediate implementation of learned skills, this workshop could be implemented at the beginning of clerkship years when students begin to interact consistently with patients. The workshop's implementation with other learners through the BNGAP conference indicates it would be applicable across multiple settings and that no prior patient experience is required.

Notably, students felt most comfortable using resilience tools as opposed to screening for ACEs and/or toxic stress. This may have been due to the discomfort associated with asking about trauma in attendings’ clinics and our lack of providing self-care/preservation tools during the initial workshop. The emphasis on using an approach of “What's strong with you?” at the end of the workshop may have also contributed to this finding. Although exploring resilience is a tool for practicing TIC, students reported applying TIC concepts less frequently than identifying patient resilience. The questions designed to query incorporation of TIC tools and TIC concepts in the 3-month follow-up survey were vaguely phrased, which may have contributed to this finding.

### Limitations

Students’ self-rating on a 3-point instead of a 5-point Likert scale and self-reporting on behavioral change may have overinterpreted our positive findings. Students noted a lack of time for in-depth discussions in the small groups and proposed increasing this to an hour. The COVID-19 pandemic necessitated remote instruction and limited our students’ exposure to patients, thereby impacting the implementation of learned skills for some students. The lack of routine TIC in some clinical sites also impacted students’ implementation and made it challenging to screen for ACEs and/or toxic stress. Our workshop utilized facilitators from pediatrics and family medicine and none from other specialties.

### Future Directions

We have reformatted our surveys to reflect a 5-point scale for future use. Three-month follow-up surveys have been updated to measure specific TIC tools and concepts used by students as well as skills gained. We are in the process of gathering longitudinal data as students complete the first half of their clerkship year. Given the amount of content and stages of learners, we recommend future workshops be at least 1.5–2 hours and incorporate live or standardized patient interviews or guest speakers. We have updated our materials (Appendix B) to reflect a recommended workshop outline that can be used for both virtual and in-person settings.

Areas of future education design and implementation are the training of clinical faculty in TIC at clinical sites and the providing of portable templates of screening tools to students for use in patient care. We understand that the effects of ACEs and toxic stress are relevant across many medical specialties and thus intend to broaden specialty exposure of the facilitators in future sessions and to include multiple clinical cases in the small-group discussions.

During small-group discussions, discussing traumatic exposures may trigger students exposed to ACEs or toxic stress in their own childhood and affect their active participation. Updated workshop materials now include trigger warnings, self-care/preservation strategies, and protocols^27^ for distressed students developed with our director of medical student wellness. We recommend facilitators be familiar with preferred strategies and protocols before small-group sessions.

As we emerge from the COVID-19 pandemic and its traumatic effects, using an approach of “What happened to you?” and an approach of “What's strong with you?” creates avenues to build relationship-centered care and incorporate TIC to maximize opportunities for equitable health in patients.

## Appendices


ACEs and Health Equity Slides.pptxFacilitator Guide.docxFacilitator Slides.pptxStudent Handout.docxPre-, Post-, and 3-Month Follow-up Surveys.docx
*All appendices are peer reviewed as integral parts of the Original Publication.*

## References

[1] Anda RF, Felitti VJ, Bremner JD, et al. The enduring effects of abuse and related adverse experiences in childhood: a convergence of evidence from neurobiology and epidemiology. Eur Arch Psychiatry Clin Neurosci. 2006;256(3):174–186. 10.1007/s00406-005-0624-4 16311898 PMC3232061

[2] Metzler M, Merrick MT, Klevens J, Ports KA, Ford DC. Adverse childhood experiences and life opportunities: shifting the narrative. Child Youth Serv Rev. 2017;72:141–149. 10.1016/j.childyouth.2016.10.021 PMC10642285 37961044

[3] Recognizing and treating child traumatic stress. Substance Abuse and Mental Health Services Administration. Updated November 5, 2021. Accessed January 7, 2022. https://www.samhsa.gov/child-trauma/recognizing-and-treating-child-traumatic-stress

[4] Shonkoff JP, Garner AS; Committee on Psychosocial Aspects of Child and Family Health; Committee on Early Childhood, Adoption, and Dependent Care; Section on Developmental and Behavioral Pediatrics. The lifelong effects of early childhood adversity and toxic stress. Pediatrics. 2012;129(1):e232–e246. 10.1542/peds.2011-2663 22201156

[5] Ten leading causes of death and injury—images. Centers for Disease Control and Prevention. Updated June 24, 2020. Accessed January 7, 2022. https://www.cdc.gov/injury/wisqars/LeadingCauses_images.html

[6] Health equity. Centers for Disease Control and Prevention. Updated March 11, 2020. Accessed January 12, 2022. https://www.cdc.gov/chronicdisease/healthequity/index.htm

[7] Marmot M, Friel S, Bell R, Houweling TAJ, Taylor S; Commission on Social Determinants of Health. Closing the gap in a generation: health equity through action on the social determinants of health. Lancet. 2008;372(9650):1661–1669. 10.1016/S0140-6736(08)61690-6 18994664

[8] Let's Talk: moving upstream. National Collaborating Centre for Determinants of Health. 2014. Accessed January 7, 2022. https://nccdh.ca/resources/entry/lets-talk-moving-upstream

[9] Andersen JP, Blosnich J. Disparities in adverse childhood experiences among sexual minority and heterosexual adults: results from a multi-state probability-based sample. PLoS One. 2013;8(1):e54691. 10.1371/journal.pone.0054691 23372755 PMC3553068

[10] Braveman P, Barclay C. Health disparities beginning in childhood: a life-course perspective. Pediatrics. 2009;124(suppl 3):S163–S175. 10.1542/peds.2009-1100D 19861467

[11] Kenney MK, Singh GK. Adverse childhood experiences among American Indian/Alaska Native children: the 2011–2012 National Survey of Children's Health. *Scientifica* *(Cairo)*. 2016; 2016:7424239. 10.1155/2016/7424239 PMC4977380 27529052

[12] Messina N, Grella C. Childhood trauma and women's health outcomes in a California prison population. Am J Public Health. 2006;96(10):1842–1848. 10.2105/AJPH.2005.082016 17008581 PMC1586137

[13] Mohatt NV, Thompson AB, Thai ND, Tebes JK. Historical trauma as public narrative: a conceptual review of how history impacts present-day health. Soc Sci Med. 2014;106:128–136. 10.1016/j.socscimed.2014.01.043 24561774 PMC4001826

[14] Liu SR, Kia-Keating M, Nylund-Gibson K. Patterns of adversity and pathways to health among White, Black, and Latinx youth. Child Abuse Negl. 2018;86:89–99. 10.1016/j.chiabu.2018.09.007 30273815

[15] Font SA, Maguire-Jack K. Pathways from childhood abuse and other adversities to adult health risks: the role of adult socioeconomic conditions. Child Abuse Negl. 2016;51:390–399. 10.1016/j.chiabu.2015.05.013 26059537 PMC4670808

[16] SAMHSA's concept of trauma and guidance for a trauma-informed approach. National Institute of Corrections. October 10, 2014. Accessed January 7, 2022. https://nicic.gov/samhsas-concept-trauma-and-guidance-trauma-informed-approach

[17] Forkey HC, Griffin JL, Szilagyi M. Childhood Trauma and Resilience: A Practical Guide. American Academy of Pediatrics; 2021. 10.1542/9781610025072

[18] Pediatric Approach to Trauma, Treatment and Resilience Project. American Academy of Pediatrics. Accessed May 11, 2021. https://www.aap.org/en-us/advocacy-and-policy/aap-health-initiatives/resilience/related-initiatives/Pages/Pediatric-Approach-to-Trauma-Treatement-and-Resilience-Project.aspx

[19] Center on the Developing Child at Harvard University. Harvard University. Updated March 13, 2015. Accessed May 11, 2021. https://developingchild.harvard.edu/

[20] McEwen CA, McEwen BS. Social structure, adversity, toxic stress, and intergenerational poverty: an early childhood model. Annu Rev Sociol. 2017;43:445–472. 10.1146/annurev-soc-060116-053252

[21] Centers for Disease Control and Prevention. Preventing Adverse Childhood Experiences (ACEs): Leveraging the Best Available Evidence. National Center for Injury Prevention and Control, Centers for Disease Control and Prevention; 2019. https://www.cdc.gov/violenceprevention/pdf/preventingACES.pdf

[22] Onigu-Otite E, Idicula S. Introducing ACEs (adverse childhood experiences) and resilience to first-year medical students. MedEdPORTAL. 2020;16:10964. 10.15766/mep_2374-8265.10964 32964120 PMC7499813

[23] Goldstein E, Murray-García J, Sciolla AF, Topitzes J. Medical students’ perspectives on trauma-informed care training. Perm J. 2018;22(1):17–126. 10.7812/TPP/17-126 PMC5798942 29401053

[24] Pletcher BA, O'Connor M, Swift-Taylor ME, DallaPiazza M. Adverse childhood experiences: a case-based workshop introducing medical students to trauma-informed care. MedEdPORTAL. 2019;15:10803. 10.15766/mep_2374-8265.10803 30931382 PMC6415008

[25] Elisseou S, Puranam S, Nandi M. A novel, trauma-informed physical examination curriculum for first-year medical students. MedEdPORTAL. 2019;15:10799. 10.15766/mep_2374-8265.10799 30800999 PMC6376894

[26] Smidt A, Balandin S, Sigafoos J, Reed VA. The Kirkpatrick model: a useful tool for evaluating training outcomes. J Intellect Dev Disabil. 2009;34(3):266–274. 10.1080/13668250903093125 19681007

[27] Wolf C, Serpa JG. A Clinician's Guide to Teaching Mindfulness: The Comprehensive Session-by-Session Program for Mental Health Professionals and Health Care Providers. New Harbinger Publications; 2015.

