# Supplementary material for: Screening for Toxic Stress Response and Buffering Factors: A Case-Based, Trauma-Informed Approach to Health Equity
Source: MedEdPORTAL. 2022 Mar 4;18:11224. doi: 10.15766/mep_2374-8265.11224 (PMC8894523; doi:10.15766/mep_2374-8265.11224)
Supplement: Supplementary file 1 — ACEs and Health Equity Slides.pptxFacilitator Guide.docxFacilitator Slides.pptxStudent Handout.docxPre-, Post-, and 3-Month Follow-up Surveys.docx [file mep_2374-8265.11224-s001.zip › B. Facilitator Guide.docx]

**Facilitator Guide**

***Screening for Toxic Stress Response and Buffering Factors: A Case-based Trauma Informed Approach to Health Equity***

**Breakout Educational Objectives:**

1. Describe ACEs as determinants of Health Equity.
2. Demonstrate ACEs and toxic stress screening in patient care.
3. Identify buffering factors in patients exposed to ACEs.
4. Apply three brief in-clinic Trauma-Informed Care resilience-fostering techniques for patients exposed to ACEs

**Participants per group & timing:** Please no more than 10 participants per group, 90-120 minutes.

**Materials:**

- Facilitator Guide (including Facilitator Reference, Notetaking Page, and Patient Case)
- Facilitator Slides (for Facilitator to display to group, if needed)

**Participant Handouts:**

- Patient Case
- FRAYED, THREADS and 3R’s templates.

**Facilitator roles:**

- To keep track of time for breakout, cover all questions, support discussion and equal participation.
- Call on small groups to initiate discussion during report out to large group sessions.
- Direct distressed participants to self-care/ preservation practice tools and designated resource person.

**Self-Care/Preservation Practice Tools:**

List tool(s) to be used by participants when distressed during the session.

Facilitators need to be trained and familiar with the tools before the session.

**S**- Stop for just a moment. Don’t react. Give yourself a moment.

**T**-Take a breath. Breath in and out. Sense the chest rising and falling.

**O**-Observe your experience- Notice the sensations in the body. Observe the thoughts or the story going through your mind and appreciate that thoughts are not facts.
**P**- Proceed with intentionality. Move forward with what feels right for you.

**Protocol for Distressed Participants (virtual sessions):**

Please review with participants before small group discussions.

1. Notify facilitator by private chat.
2. Your facilitator will refer to designated person for wellness for further support.
3. Please schedule a follow up with designated person for wellness after the session if needed.

**Protocol for Distressed Participants (for in-person sessions)**

Please review with participants before small group discussions

1. Student may exit the discussion room.
2. Your facilitator will refer to designated person for wellness for further support.
3. Please schedule a follow up with designated person for wellness after the session if needed.

**Session Outline for Virtual and In-person Settings.**

| **Topic** | **Duration** | **Details** |
| --- | --- | --- |
| Introduction to self-care/preservation tool. | 5 min | Introduction to and demonstration of self-care/preservation tool and practice by designated wellness resource person. |
| Overview of Toxic stress and Buffering factors | 10 min | Designated faculty will give a brief overview on ACEs, toxic stress, buffering/resilience and how they act as determinants of health equity. |
| Patient Perspective/Interview | 10 min | Invited patient/community member will share their story reflections/experience on toxic stress, buffering factors, resilience and health equity. |
| Pause Write Exercise | 5 min | Students write in 3-5 sentences, initial thoughts about a clinical case or patient perspective using self-care/preservation tool before transitioning into small groups. |
| Students transition into small groups for discussion and dialogue | | |
| Small Group Discussion: Identifying ACEs and Toxic Stress | 30 min | Screening for toxic stress in clinical case scenario using FRAYED and discuss how ACEs /toxic stress are health equity determinants. |
| Small Group Discussion: Identifying Buffering and Resilience Factors | 30 min | Identifying buffering factors in clinical case scenario using THREADS and discussing TIC resilience fostering with 3Rs |
| Report out to large group | 20 min | Each group reports on findings for ACE score, FRAYED, THREADS, 3Rs and how ACEs/toxic stress are health equity determinants. |
| Closing plenary: Shift from “What’s wrong with you?” to “What happened to you?” and What’s strong with you?” | 10 min | Students reflect on how they plan to use concepts in patient care, anticipated challenges, and utilization of self-care skills. |

***Please review protocol and resources for distressed participants before small group discussions begin.**

**Clinical Case**

Author: Adwoa Osei, MD

A 12-year-old boy, “Henry,” is brought in by his mother for a new well child visit. She wants to learn how to be a better parent.

Birth/Developmental/Medical History

Henry was born at term. Henry’s dad was mostly absent during the pregnancy and mom reports “feeling sad and crying all the time.” She was diagnosed with postpartum depression and struggled parenting him. She "yelled a lot at him.” She also reports feeling “unsafe” when dad came around but refused to specify why. She did not take any unprescribed medications, addictive substances, and alcohol before or during her pregnancy. She kept up with all her prenatal visits. During the visit, mom became teary eyed, as she talked about her partner’s absence during the pregnancy. She confided that “it reminded her of her father.” Apart from a history of "prolonged temper tantrums," he met all his developmental milestones. He has fairly controlled asthma and misses school because of wheezing.

Social History

Henry lives with his mother and maternal grandmother, their main source of support. His maternal uncles visit often, and he enjoys having them around. His dad visits occasionally, unannounced. Mom dropped out of college and is unemployed due to multiple chronic health needs. When questioned alone and directly about his father, Henry reports “I feel unloved by my father.” “There is no point in being alive.” He feels unsafe when his dad does visit but refuses to say why. “It’s like we have to walk on eggshells around him.” He is also sad about the death of his “favorite uncle” 2 years ago to witnessed assault in his neighborhood. He was like a father to him. He is afraid “his mother will die too.” He is happy to have his grandmother and mom who care “deeply about him.” He shrugs his shoulders when asked about school. He is in 6^th^ grade and struggles with his schoolwork. He reports "I don’t get math and the words don’t make sense when I read. School is boring.” Henry confides in you that this is his third school and kids always pick on him. He barely speaks to anyone in school because the kids are “mean and dumb.” He is up till 2am playing games most nights and struggles to wake up for school in the mornings. When asked to limit screen time by mom, he becomes “very angry and threatens to burn down the house.” He also sneaks food into his room and eats through the night. Video games and attending church with his mother make him happy. He would like to study coding and programming in future.

Family History

Mom lives with generalized anxiety disorder, depression, pseudo seizures, diabetes, and hypertension. She had a cerebrovascular accident a few years ago that affects her memory. She lost her father at age 10, to witnessed violent assault in their neighborhood. Maternal grandfather lived with addiction illness.

Objective and Physical Findings

Depression screening shows a high risk for moderate to severe depression. Screening for alcohol and substance abuse is negative. He has attention difficulties. He has no thoughts of suicidal or homicidal ideations. He avoids eye contact and plays on his phone through the visit. BMI > 99% (Normal range: 5%-85%).

**Small Group Discussion**

*1. From the history provided, provide an ACE score for Henry (~5min)*

**ACE Score for Henry- Minimum score of 4**

Parental separation

Witness to physical assault

Maternal mental illness

Death of uncle

Possible bullying

*2. Using the FRAYED acronym, what symptoms do you note in Henry? (~5min)*

| **F**its, **F**rets and **F**ear |
| --- |
| **R**estricted development |
| **A**ttachment concerns |
| **Y**elling and yawning |
| **E**ducational delays/Eating problems |
| **D**efeated /Dissociation/Disease |

**Henry FRAYED**

F- fear of dad, angry outbursts, prolonged temper tantrums

R- limited friendships in school, attention difficulties

A- strained relationship with dad, few friendships in school

Y- angry outbursts, prolonged tantrums. Poor sleep hygiene

E- learning challenges in school, sneaking food into room BMI>99%

D- depressed, passive thoughts of self-harm, apathy regarding academics, BMI>99%

*3. Using the FRAYED template, what symptoms and signs of toxic stress do you note in his mother? (~5min)*

**Mom FRAYED**

F- yelling when henry was younger, fear of Henry’s dad, generalized anxiety disorder.

R- unemployment

A- struggles with parenting Henry as a baby

Y- yelling at her son.

E- Stalled college completion.

D- multiple chronic health problems

*4. Briefly describe how ACEs and toxic stress might contribute to health and social inequities in the case above. (~15min)*

Childhood adversity or trauma such as abuse and neglect, parental substance abuse and incarceration, oftentimes are rooted in inequities in communities with poverty, poor housing conditions, higher risk to violence and victimization, and homelessness. Cumulative and prolonged ACES are associated with poor social outcomes, including impaired worker performance, unemployment, self-reported disability, and a shortened life span. These cumulative and prolonged traumatic experiences can be described as social determinants of health that affect not just individuals but also families, communities, and society. Since ACES are a major contributor to health disparities, they can be seen to operate in tandem with social determinants of health.

- *Describe how the signs and symptoms of toxic stress in Henry’s mother has affected parenting, education, employment, and health?*
- *How has she been affected by her father’s death and addiction illness? How could violence in the neighborhood be contributing to her experience of trauma?*
- *How are Henry’s community adverse experiences impacting his health and education?*
- *How is Henry’s toxic stress response to trauma impacting his health and education?*

***Deeper Dive:*** *How may race or ethnicity play a role in the pursuit of health equity if this family is Black, White, Latino/x, First Nation/Native American, Asian or Pacific Islander? This question should elicit elements of structural and systemic racism. Students may explore unique cultural challenges for the groups listed. Encourage students to delve deeper by asking them to consider systemic, cultural, and structural factors that may affect health equity.*

Vulnerable and historically disadvantaged populations tend to have disproportionately higher prevalence of ACEs and ACE scores. Not coincidentally, these same populations are often burdened by social determinants of health such as experiences of racism, sexism, poor access to high quality and affordable housing and food, poverty, and inequitable education and health care.

***Summary point: ACEs are highly prevalent and affect all communities, but consequences are more prevalent in marginalized groups. ACEs and its effects can be intergenerational. This may perpetuate health, social and economic inequities.***

**Small Group Discussion (cont.)**

*1. Using the THREADS acronym, what buffering and protective factors do you note for Henry. (~5min)*

| **T**hinking and learning brain |
| --- |
| **H**ope |
| **R**egulation or self-control |
| **E**fficacy |
| **A**ttachment |
| **D**evelopmental skill mastery |
| **S**ocial connectedness |

**Henry THREADS**

T- able to navigate video games.

H- coding and programming in future, likes church.

R- no alcohol or substance use

E- willingness to engage with provider in visit.

A- mom, grandmother, uncles

D- adept at video games

S- church, video gaming community, maternal uncles

*2. Using the THREADS template, what buffering and protective factors do you note for his mother? (~5min)*

**Mom THREADS**

T- Made it into college.

H- seeking ways to help Henry.

R- no harmful substances or alcohol during pregnancy

E- continues to parent Henry with significant chronic medical history, seeks preventive care for him.

A- Henry, maternal grandmother

D- bringing Henry in for appointments, honored prenatal visits.

S- Church

*3. Describe how you would provide some TIC resilience fostering tools in today’s visit using the 3 R’s (~20min)*

| **R**eassuring/Restoring Safety and Hope |
| --- |
| **R**estoring Routine |
| **R**elaxation and Regulation |

**Reassuring/Restoring Safety and Hope:** Remind Henry that his mom, grandma and uncles will keep him safe and look out for him. Remind him of his church community. Congratulate him on having plans for becoming a programmer and coder. Remind him his brain continues to learn new things and in new ways. Remind Mom she has kept Henry safe from infancy till now. Remind her of church community, grandmother, and her uncles. Connect family to community resources to bolster connections and mentoring.

**Restoring Routine:** Discuss sleep hygiene and mealtime and snack routines. Encourage Henry to pick scheduled times for video games. Maybe play video games with uncles or mom. Scheduled time in nature/outside with trusted adult or peers.

**Relaxation and Regulation:** Elicit cultural and faith values important to family. Discuss relaxation techniques (e.g., breathing exercises, body scan) with family. Discuss mindful eating habits. Encourage cooking skills (with family’s collaboration) to enhance knowledge of nutritious meals. Together with family, identify calming/soothing skills beneficial to both Henry and mom. Refer for Trauma Informed mental health counseling.

**Large Group Discussion**

**Report out to large group (~***20min)*. Students return to the large group to report on their discussions and dialogue that occurred during small group session.

**Closing plenary: Shift from “What’s wrong with you?” to “What happened to you?” and “What’s strong with you?”** (~*10min)*. Students should reflect and share when and how they plan to use concepts in patient care, anticipated challenges, and utilization of self-care skills in the large group.

**Optional resources for discussion**

1. <https://www.cdc.gov/violenceprevention/aces/riskprotectivefactors.html>
2. <https://ebookcentral.proquest.com/lib/ucr/reader.action?docID=4004183&ppg=22> Refer to Chapter 1
3. <https://www.chcs.org/media/TA-Tool-Screening-for-ACEs-and-Trauma_020619.pdf>
