# Supplementary material for: Screening for Toxic Stress Response and Buffering Factors: A Case-Based, Trauma-Informed Approach to Health Equity
Source: MedEdPORTAL. 2022 Mar 4;18:11224. doi: 10.15766/mep_2374-8265.11224 (PMC8894523; doi:10.15766/mep_2374-8265.11224)
Supplement: Supplementary file 1 — ACEs and Health Equity Slides.pptxFacilitator Guide.docxFacilitator Slides.pptxStudent Handout.docxPre-, Post-, and 3-Month Follow-up Surveys.docx [file mep_2374-8265.11224-s001.zip › C. Facilitator Slides.pptx]

## Slide 1
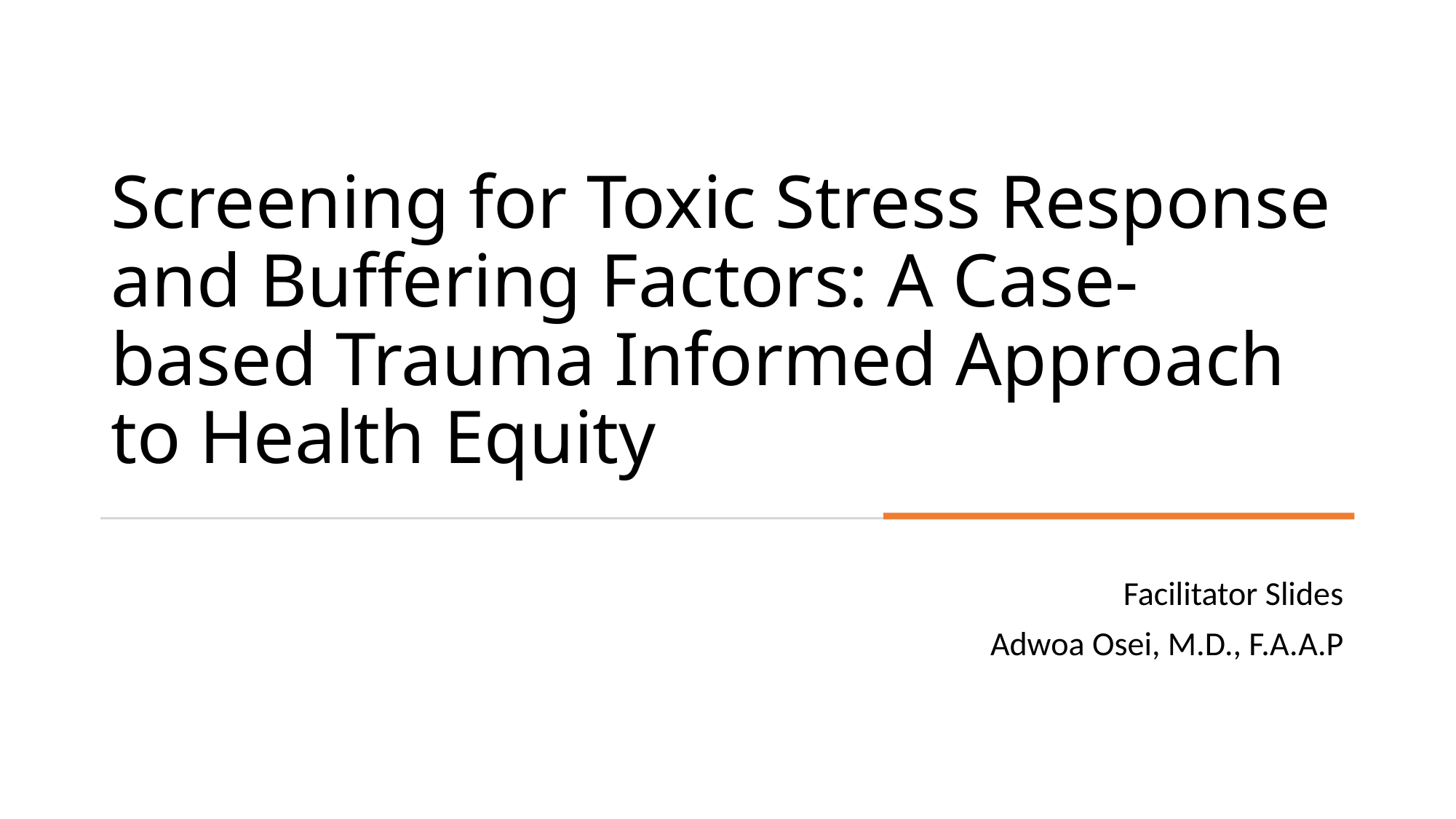

# Screening for Toxic Stress Response and Buffering Factors: A Case-based Trauma Informed Approach to Health Equity
Facilitator Slides
Adwoa Osei, M.D., F.A.A.P

## Slide 2
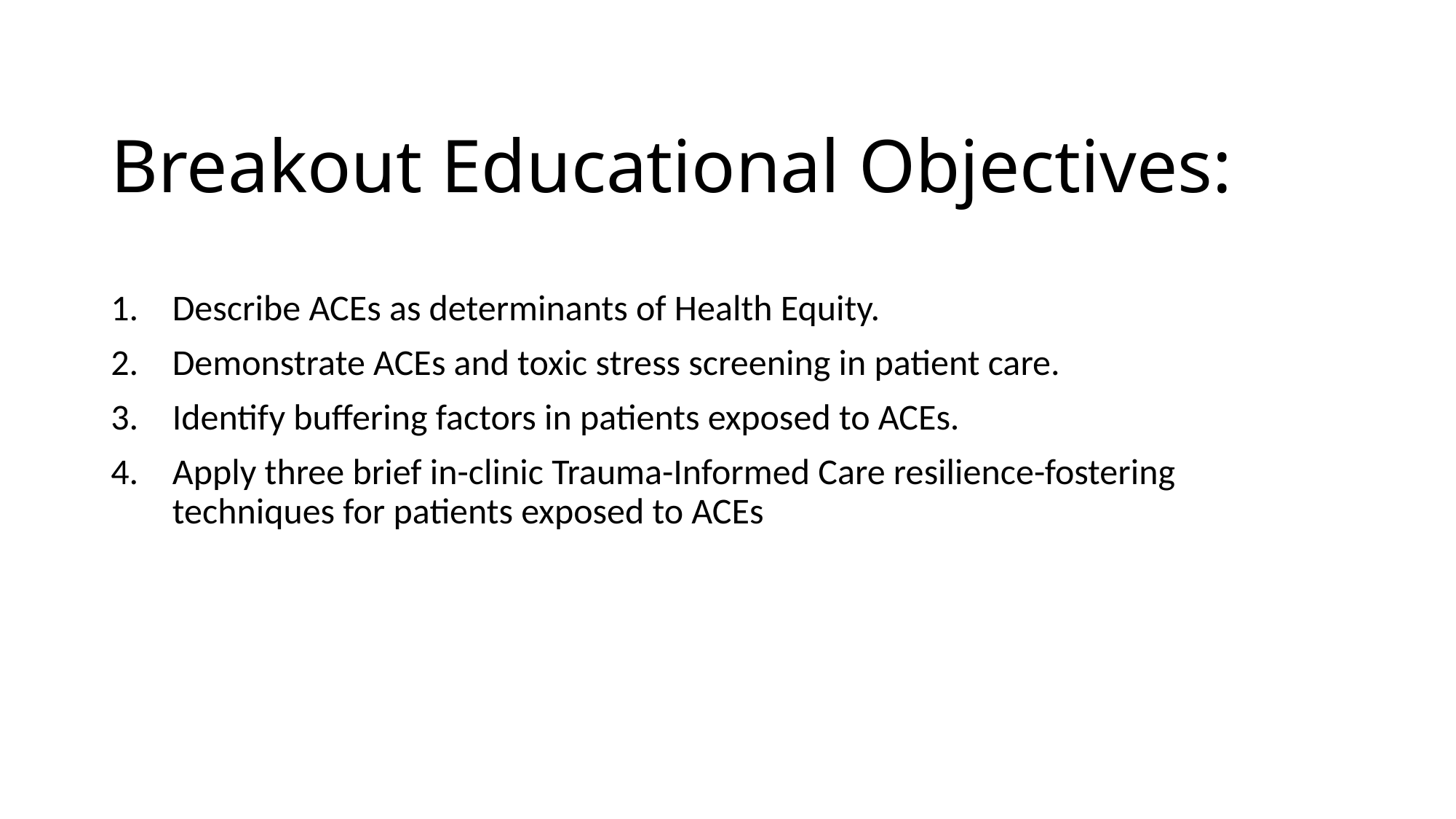

# Breakout Educational Objectives:
Describe ACEs as determinants of Health Equity.
Demonstrate ACEs and toxic stress screening in patient care.
Identify buffering factors in patients exposed to ACEs.
Apply three brief in-clinic Trauma-Informed Care resilience-fostering techniques for patients exposed to ACEs

## Slide 3
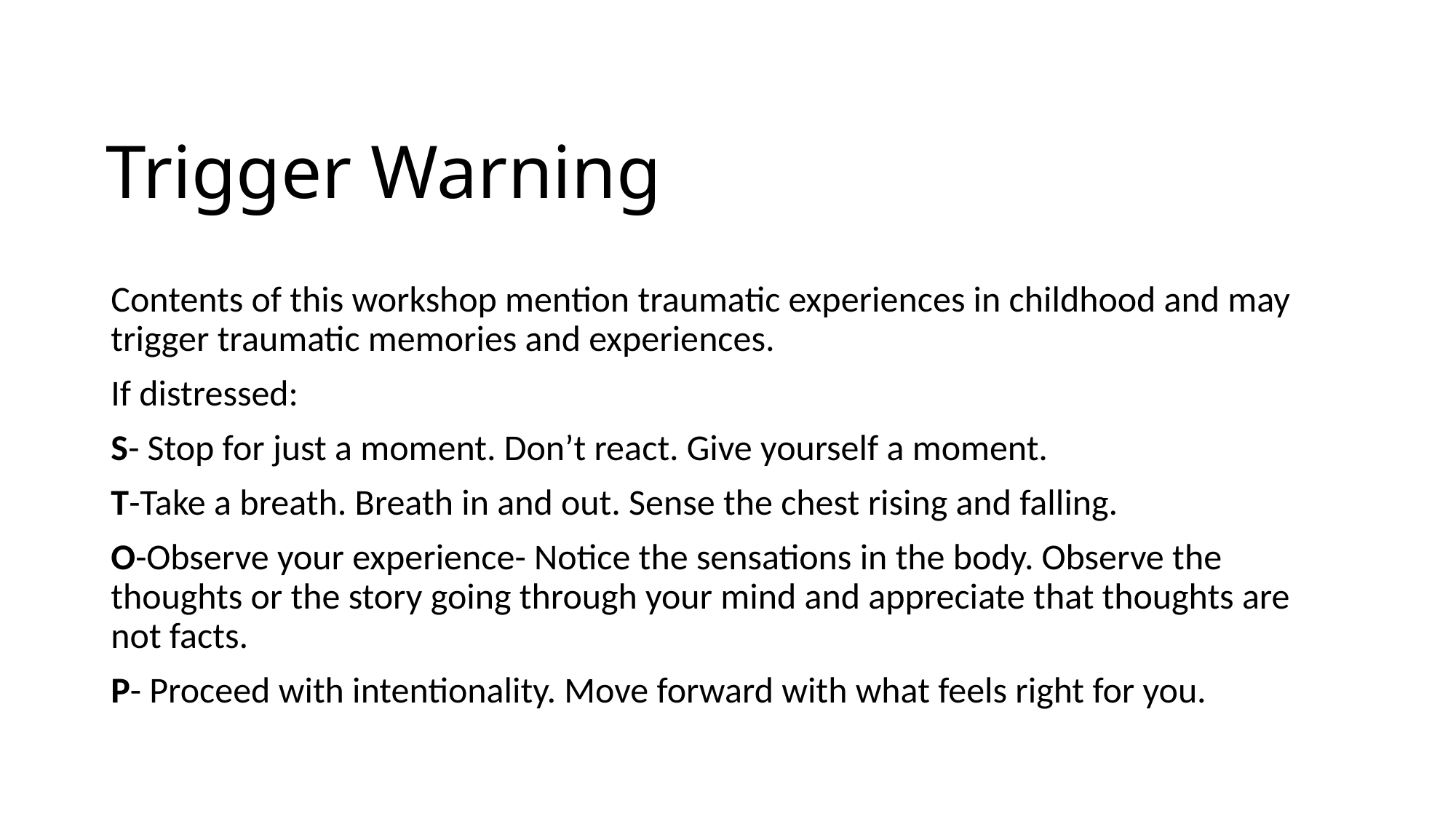

# Trigger Warning
Contents of this workshop mention traumatic experiences in childhood and may trigger traumatic memories and experiences.
If distressed:
S- Stop for just a moment. Don’t react. Give yourself a moment.
T-Take a breath. Breath in and out. Sense the chest rising and falling.
O-Observe your experience- Notice the sensations in the body. Observe the thoughts or the story going through your mind and appreciate that thoughts are not facts.
P- Proceed with intentionality. Move forward with what feels right for you.

## Slide 4
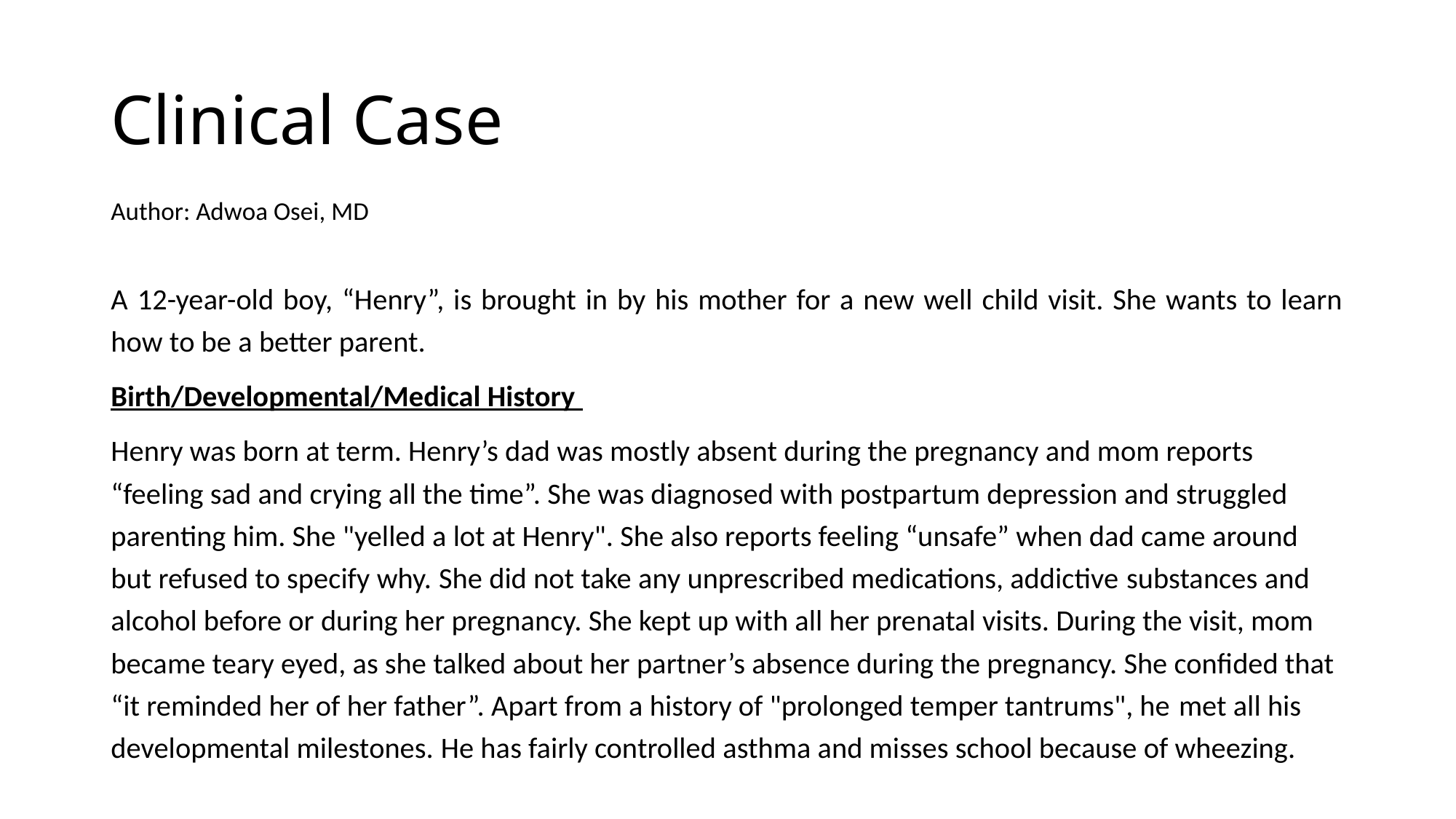

# Clinical Case
Author: Adwoa Osei, MD
A 12-year-old boy, “Henry”, is brought in by his mother for a new well child visit. She wants to learn how to be a better parent.
Birth/Developmental/Medical History
Henry was born at term. Henry’s dad was mostly absent during the pregnancy and mom reports “feeling sad and crying all the time”. She was diagnosed with postpartum depression and struggled parenting him. She "yelled a lot at Henry". She also reports feeling “unsafe” when dad came around but refused to specify why. She did not take any unprescribed medications, addictive substances and alcohol before or during her pregnancy. She kept up with all her prenatal visits. During the visit, mom became teary eyed, as she talked about her partner’s absence during the pregnancy. She confided that “it reminded her of her father”. Apart from a history of "prolonged temper tantrums", he met all his developmental milestones. He has fairly controlled asthma and misses school because of wheezing.

## Slide 5
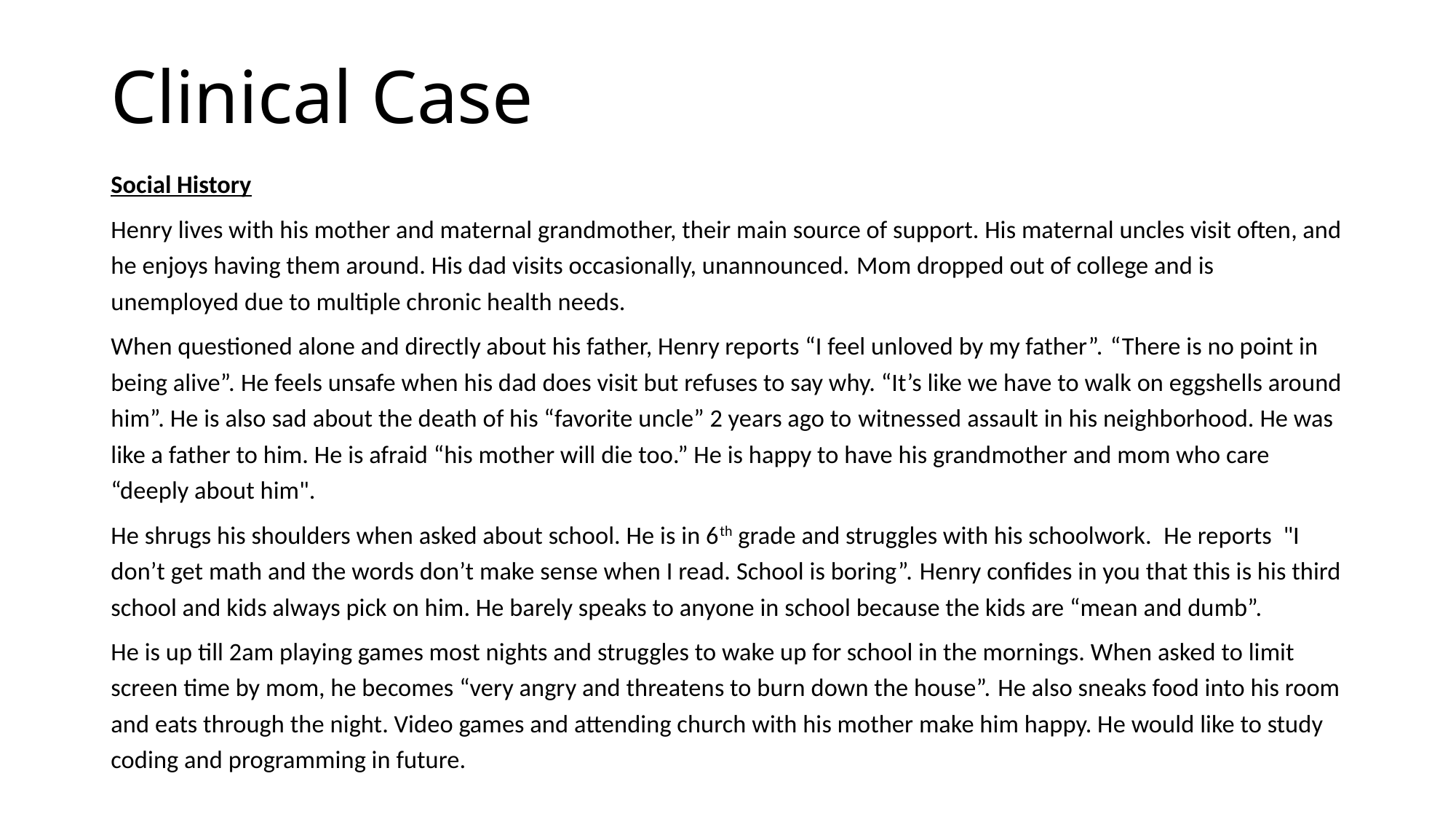

# Clinical Case
Social History
Henry lives with his mother and maternal grandmother, their main source of support. His maternal uncles visit often, and he enjoys having them around. His dad visits occasionally, unannounced. Mom dropped out of college and is unemployed due to multiple chronic health needs.
When questioned alone and directly about his father, Henry reports “I feel unloved by my father”. “There is no point in being alive”. He feels unsafe when his dad does visit but refuses to say why. “It’s like we have to walk on eggshells around him”. He is also sad about the death of his “favorite uncle” 2 years ago to witnessed assault in his neighborhood. He was like a father to him. He is afraid “his mother will die too.” He is happy to have his grandmother and mom who care “deeply about him".
He shrugs his shoulders when asked about school. He is in 6th grade and struggles with his schoolwork.  He reports  "I don’t get math and the words don’t make sense when I read. School is boring”. Henry confides in you that this is his third school and kids always pick on him. He barely speaks to anyone in school because the kids are “mean and dumb”.
He is up till 2am playing games most nights and struggles to wake up for school in the mornings. When asked to limit screen time by mom, he becomes “very angry and threatens to burn down the house”. He also sneaks food into his room and eats through the night. Video games and attending church with his mother make him happy. He would like to study coding and programming in future.

## Slide 6
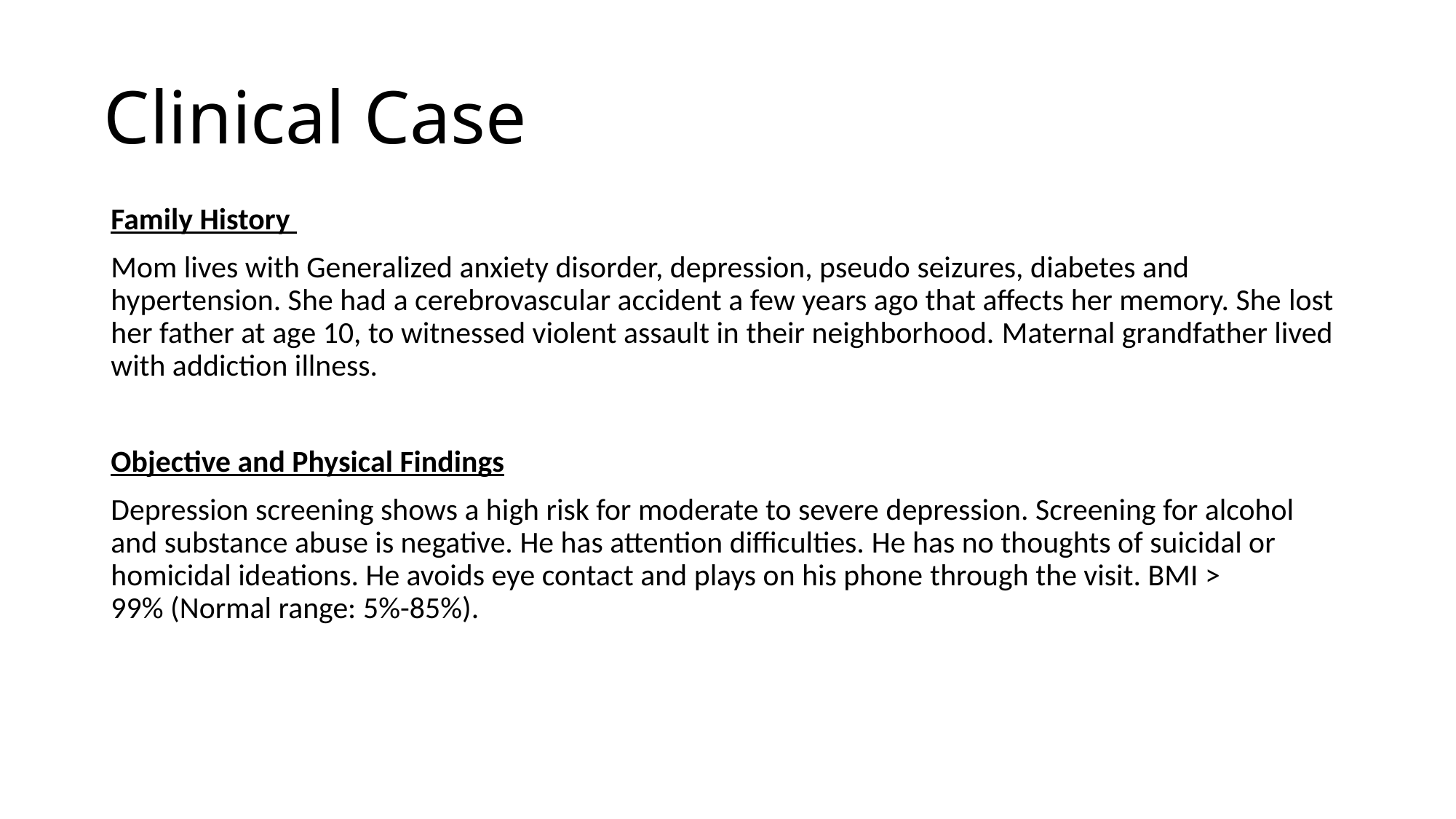

Clinical Case
Family History
Mom lives with Generalized anxiety disorder, depression, pseudo seizures, diabetes and hypertension. She had a cerebrovascular accident a few years ago that affects her memory. She lost her father at age 10, to witnessed violent assault in their neighborhood. Maternal grandfather lived with addiction illness.
Objective and Physical Findings
Depression screening shows a high risk for moderate to severe depression. Screening for alcohol and substance abuse is negative. He has attention difficulties. He has no thoughts of suicidal or homicidal ideations. He avoids eye contact and plays on his phone through the visit. BMI > 99% (Normal range: 5%-85%).

## Slide 7
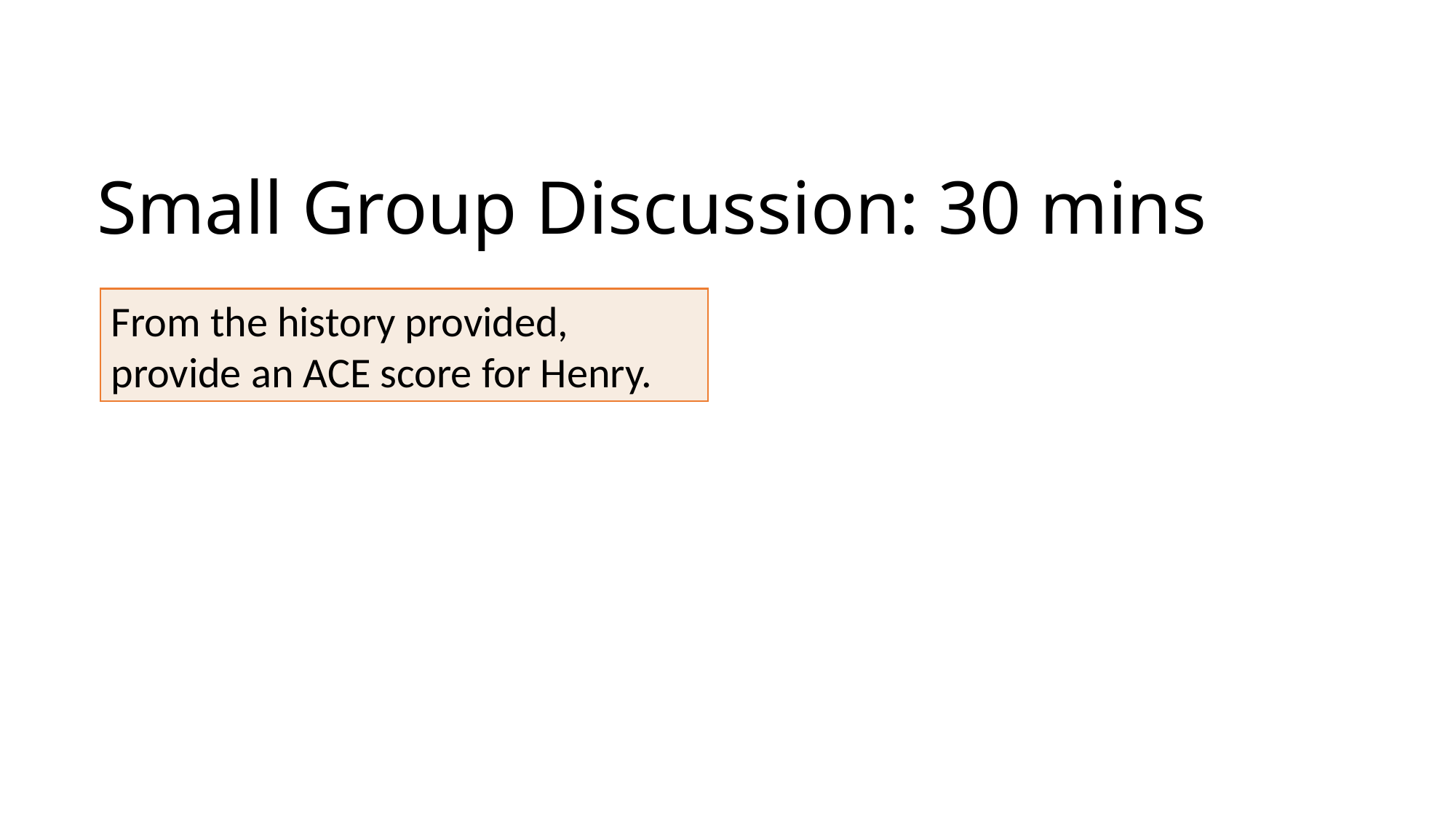

Small Group Discussion: 30 mins
From the history provided, provide an ACE score for Henry.

## Slide 8
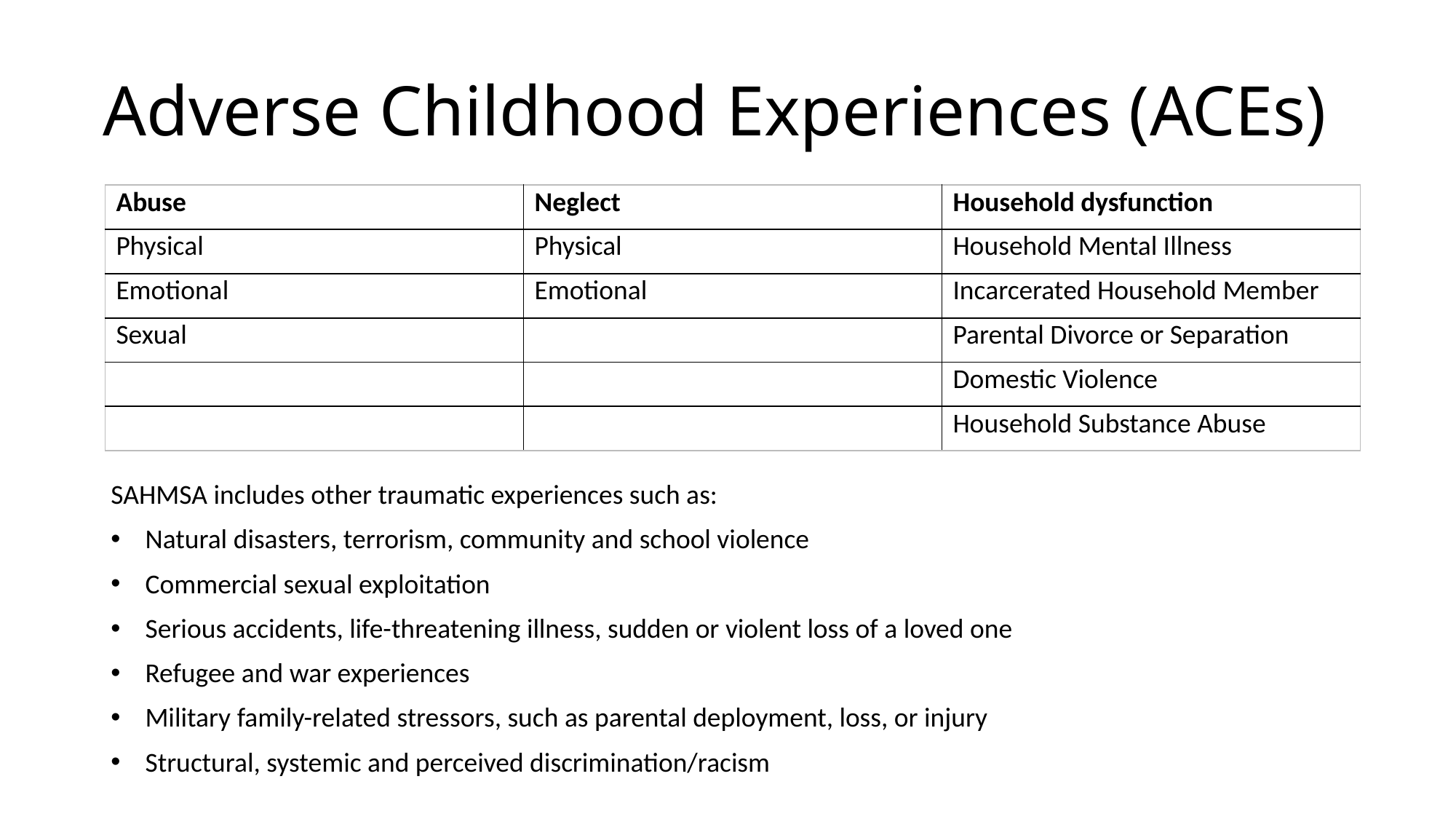

Adverse Childhood Experiences (ACEs)
| Abuse | Neglect | Household dysfunction |
| --- | --- | --- |
| Physical | Physical | Household Mental Illness |
| Emotional | Emotional | Incarcerated Household Member |
| Sexual | | Parental Divorce or Separation |
| | | Domestic Violence |
| | | Household Substance Abuse |
SAHMSA includes other traumatic experiences such as:
Natural disasters, terrorism, community and school violence
Commercial sexual exploitation
Serious accidents, life-threatening illness, sudden or violent loss of a loved one
Refugee and war experiences
Military family-related stressors, such as parental deployment, loss, or injury
Structural, systemic and perceived discrimination/racism

## Slide 9
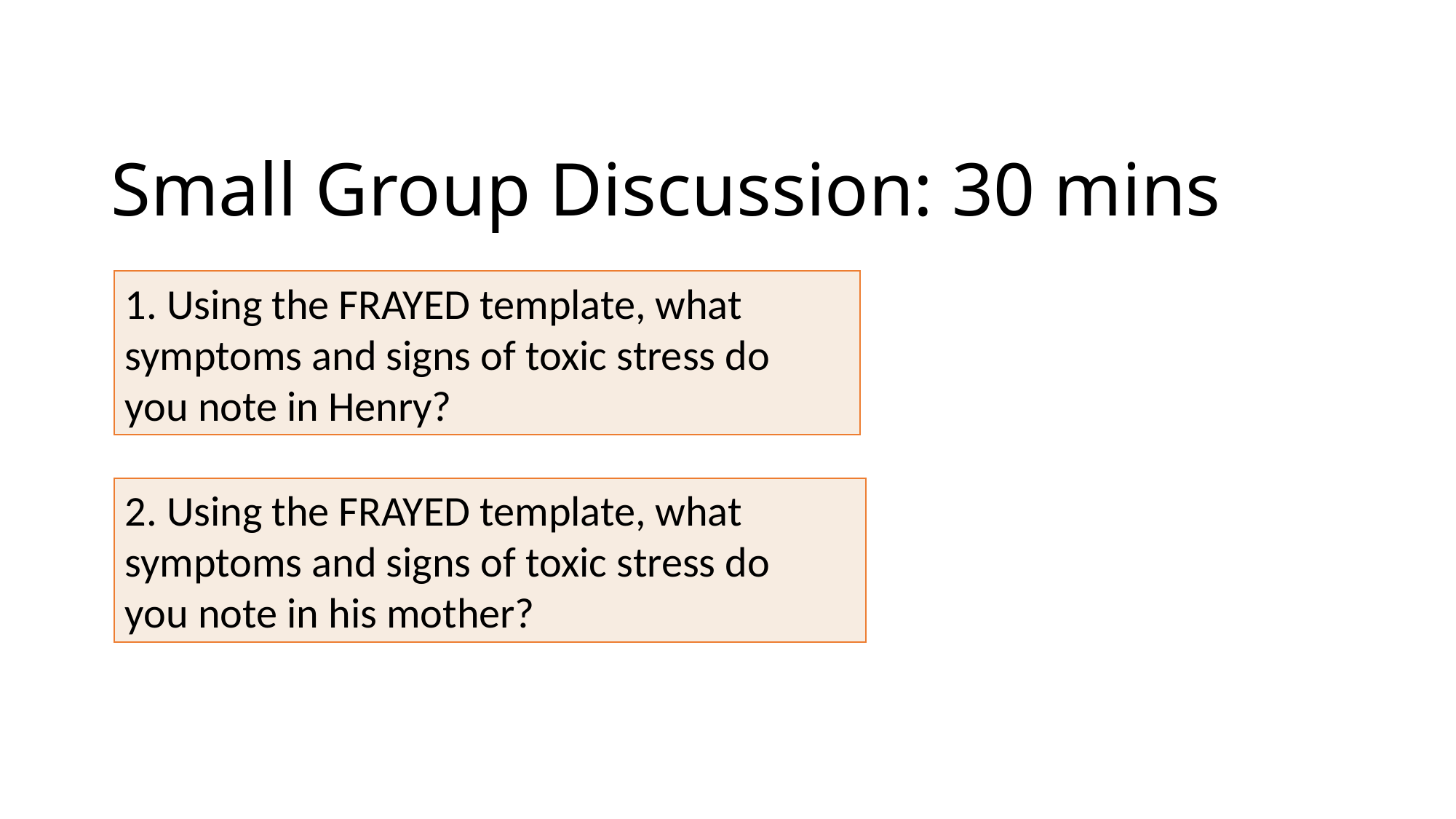

Small Group​ Discussion: 30 mins
1. Using the FRAYED template, what symptoms and signs of toxic stress do you note in Henry?
2. Using the FRAYED template, what symptoms and signs of toxic stress do you note in his mother?

## Slide 10
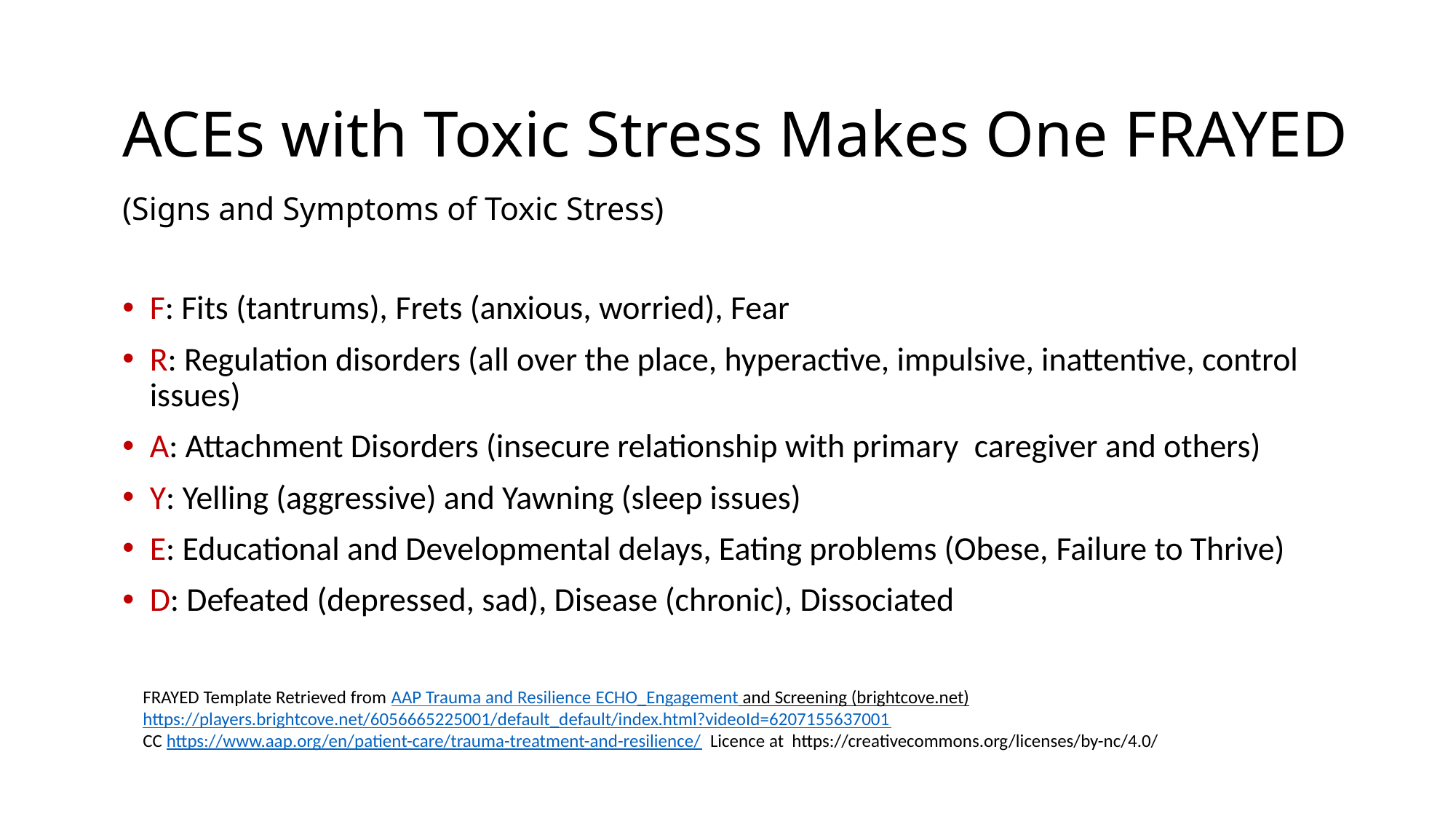

# ACEs with Toxic Stress Makes One FRAYED (Signs and Symptoms of Toxic Stress)
F: Fits (tantrums), Frets (anxious, worried), Fear
R: Regulation disorders (all over the place, hyperactive, impulsive, inattentive, control issues)
A: Attachment Disorders (insecure relationship with primary  caregiver and others)
Y: Yelling (aggressive) and Yawning (sleep issues)
E: Educational and Developmental delays, Eating problems (Obese, Failure to Thrive)
D: Defeated (depressed, sad), Disease (chronic), Dissociated
FRAYED Template Retrieved from AAP Trauma and Resilience ECHO_Engagement and Screening (brightcove.net) https://players.brightcove.net/6056665225001/default_default/index.html?videoId=6207155637001
CC https://www.aap.org/en/patient-care/trauma-treatment-and-resilience/ Licence at https://creativecommons.org/licenses/by-nc/4.0/

## Slide 11
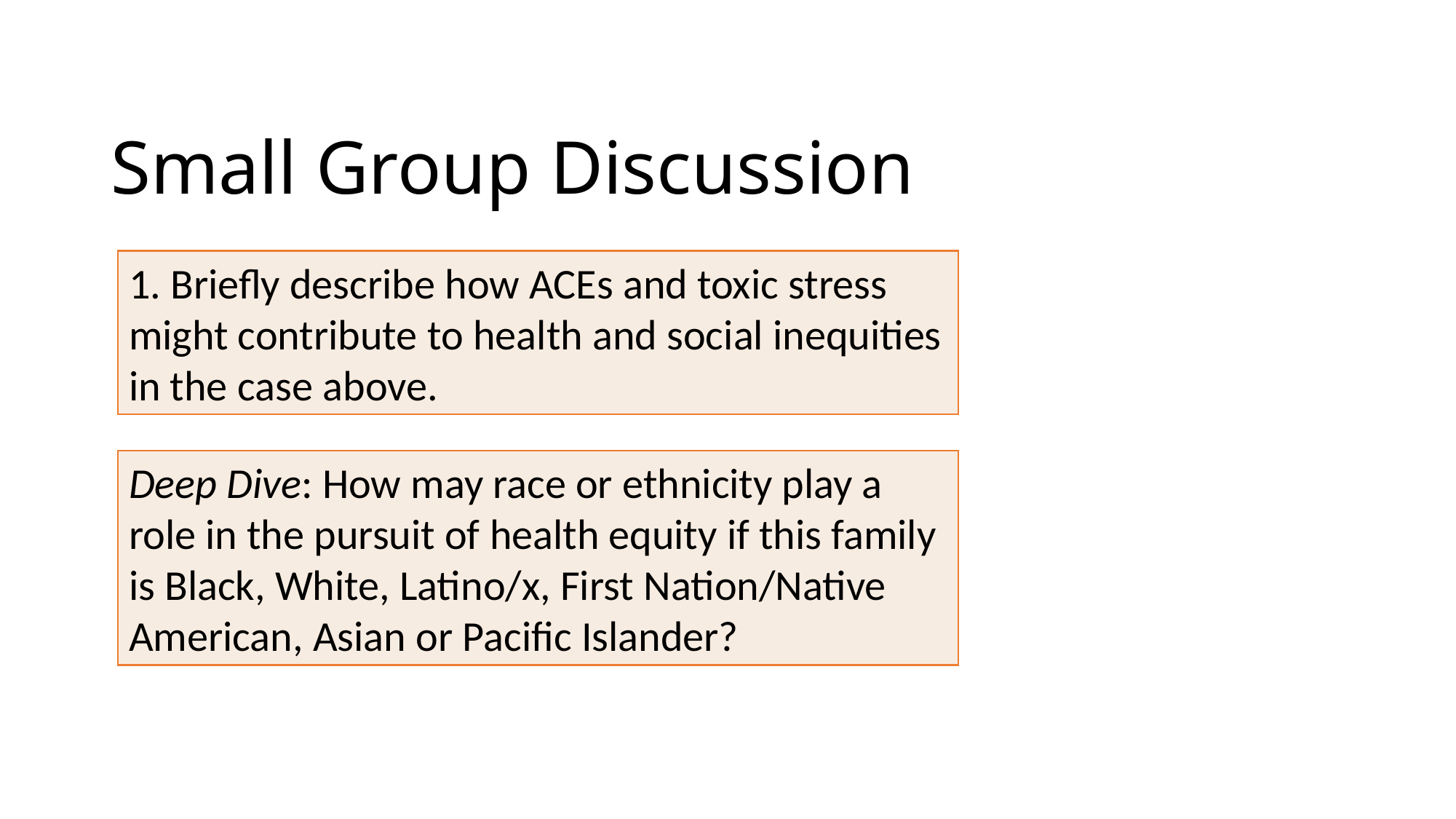

# Small Group​ Discussion
1. Briefly describe how ACEs and toxic stress might contribute to health and social inequities in the case above.
Deep Dive: How may race or ethnicity play a role in the pursuit of health equity if this family is Black, White, Latino/x, First Nation/Native American, Asian or Pacific Islander?

## Slide 12
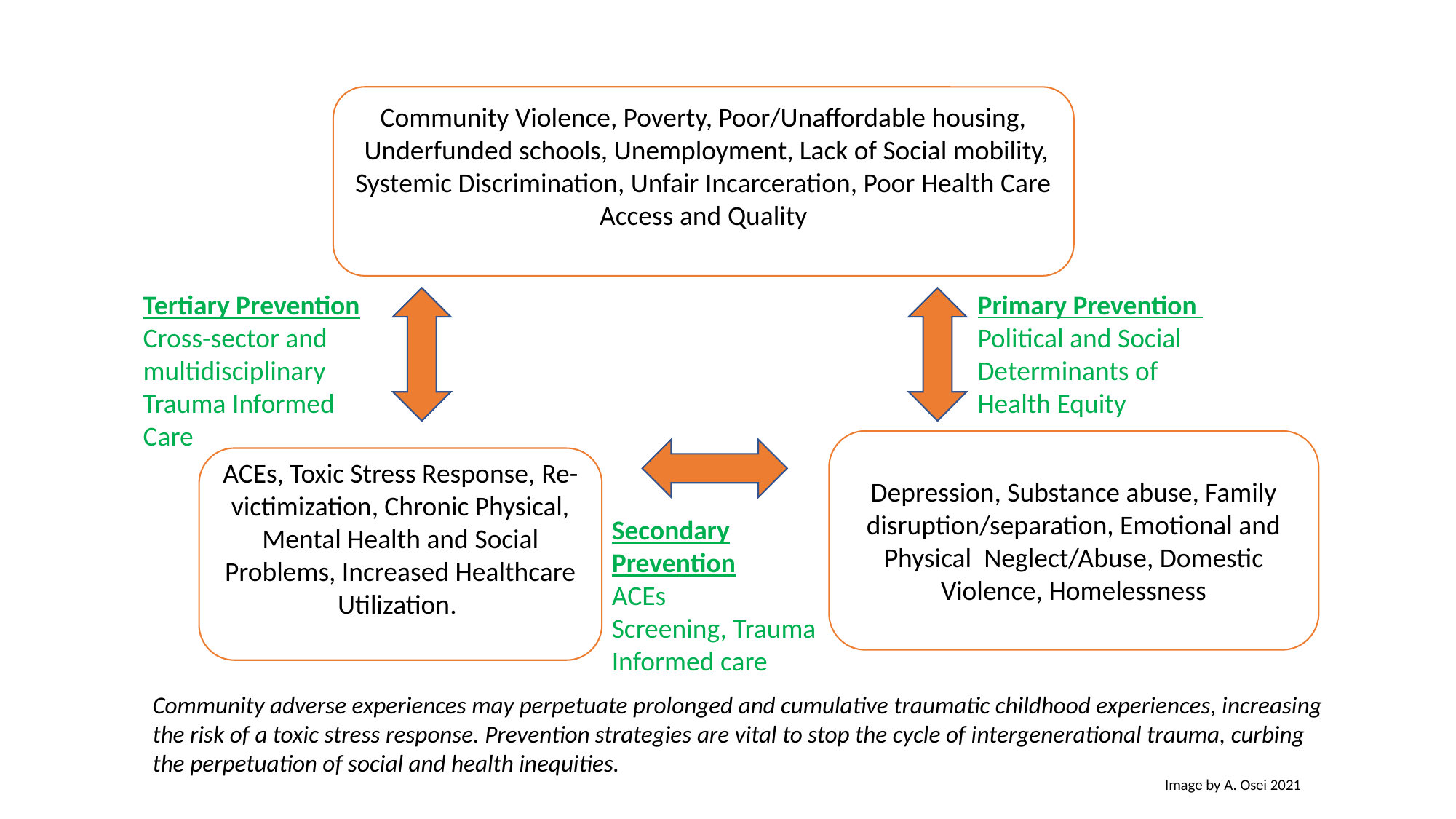

Community Violence, Poverty, Poor/Unaffordable housing,  Underfunded schools, Unemployment, Lack of Social mobility, Systemic Discrimination, Unfair Incarceration, Poor Health Care Access and Quality
Primary Prevention
Political and Social Determinants of Health Equity
Tertiary Prevention
Cross-sector and multidisciplinary Trauma Informed Care
Depression, Substance abuse, Family disruption/separation, Emotional and Physical  Neglect/Abuse, Domestic Violence, Homelessness
ACEs, Toxic Stress Response, Re-victimization, Chronic Physical, Mental Health and Social Problems, Increased Healthcare Utilization.
Secondary
Prevention
ACEs Screening, Trauma Informed care
Community adverse experiences may perpetuate prolonged and cumulative traumatic childhood experiences, increasing the risk of a toxic stress response. Prevention strategies are vital to stop the cycle of intergenerational trauma, curbing the perpetuation of social and health inequities.
Image by A. Osei 2021

## Slide 13
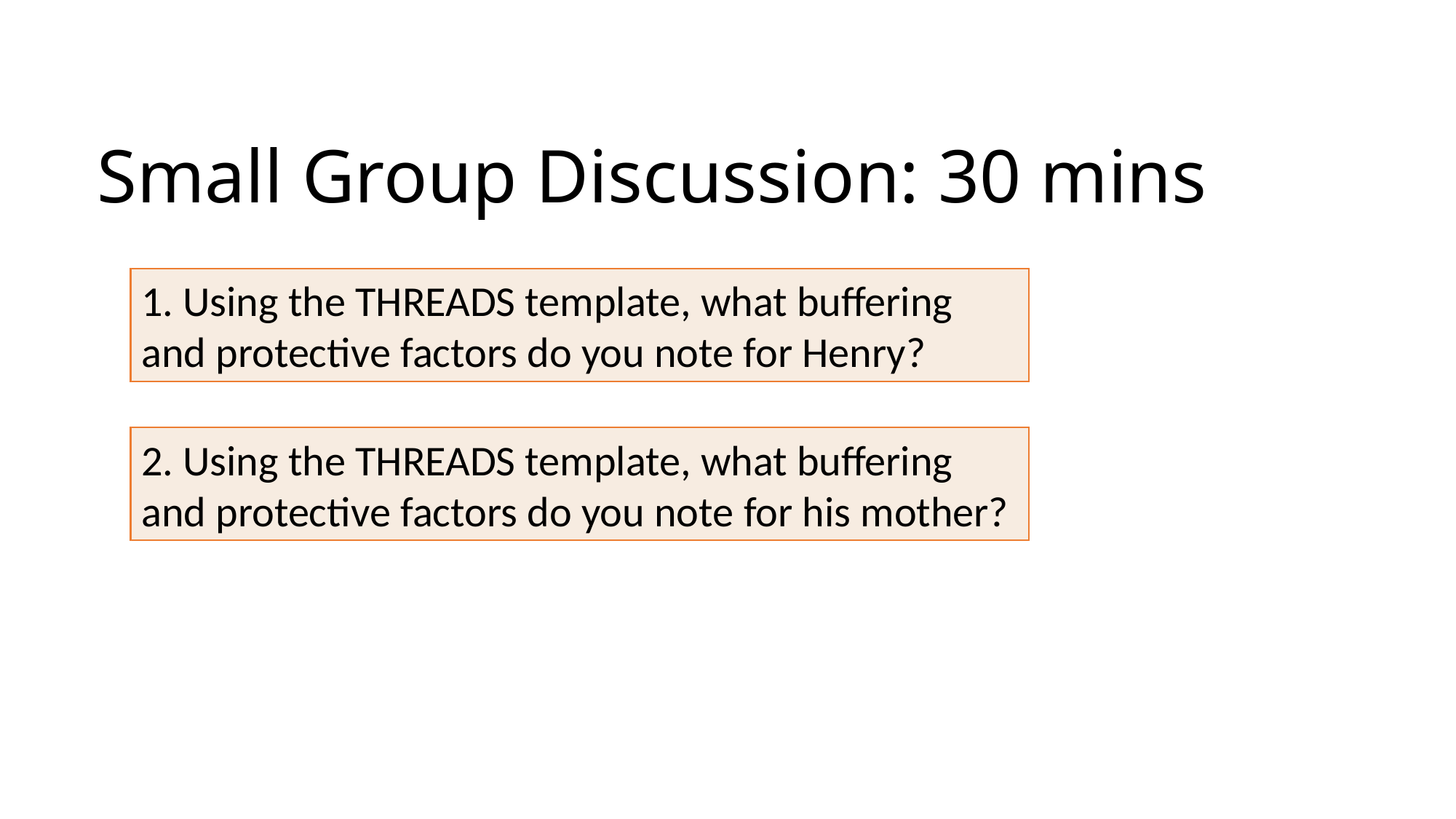

Small Group Discussion: 30 mins
1. Using the THREADS template, what buffering and protective factors do you note for Henry?
2. Using the THREADS template, what buffering and protective factors do you note for his mother?

## Slide 14
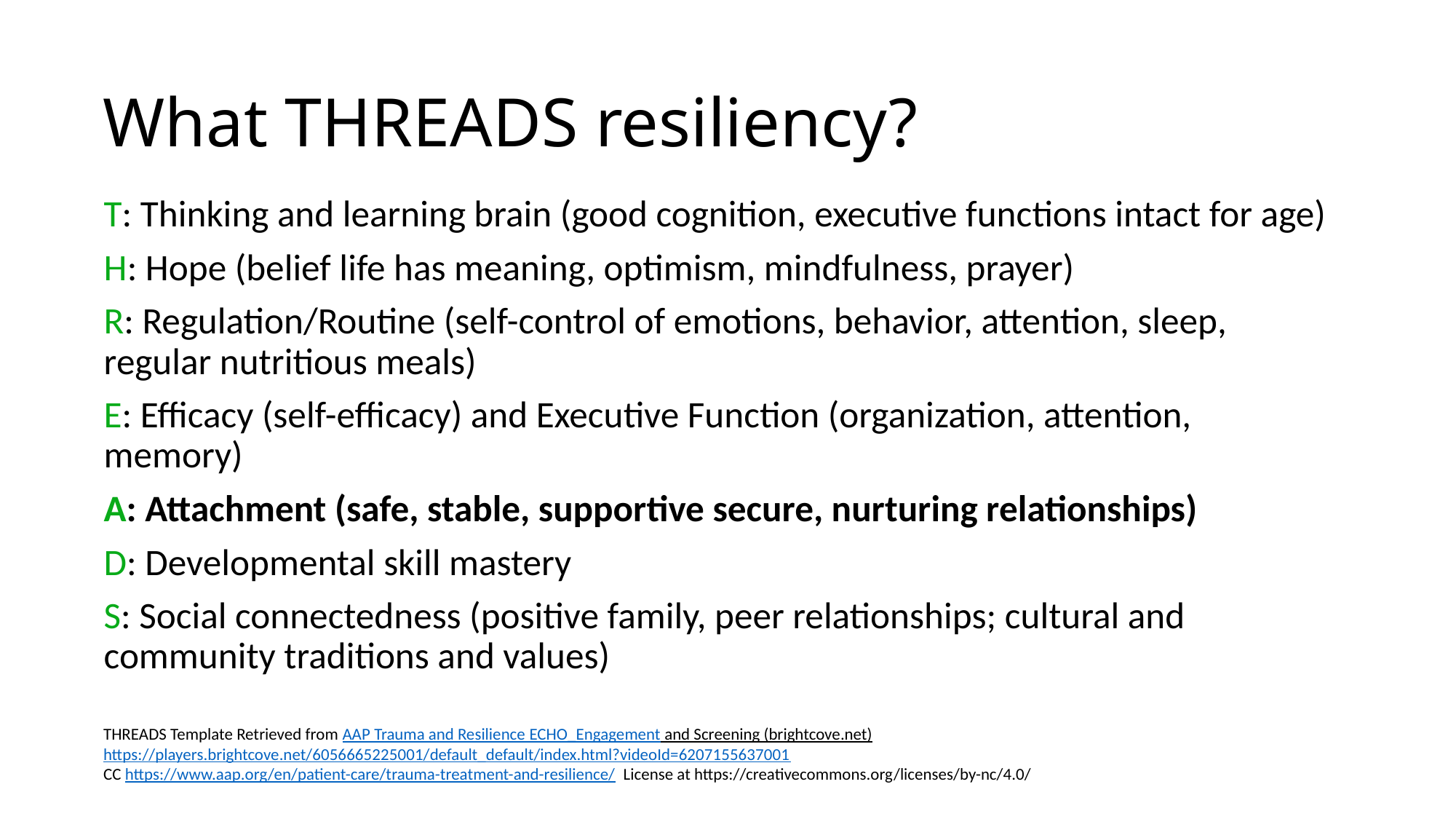

# What THREADS resiliency?
T: Thinking and learning brain (good cognition, executive functions intact for age)
H: Hope (belief life has meaning, optimism, mindfulness, prayer)
R: Regulation/Routine (self-control of emotions, behavior, attention, sleep, regular nutritious meals)
E: Efficacy (self-efficacy) and Executive Function (organization, attention, memory)
A: Attachment (safe, stable, supportive secure, nurturing relationships)
D: Developmental skill mastery
S: Social connectedness (positive family, peer relationships; cultural and community traditions and values)
THREADS Template Retrieved from AAP Trauma and Resilience ECHO_Engagement and Screening (brightcove.net)   https://players.brightcove.net/6056665225001/default_default/index.html?videoId=6207155637001
CC https://www.aap.org/en/patient-care/trauma-treatment-and-resilience/  License at https://creativecommons.org/licenses/by-nc/4.0/

## Slide 15
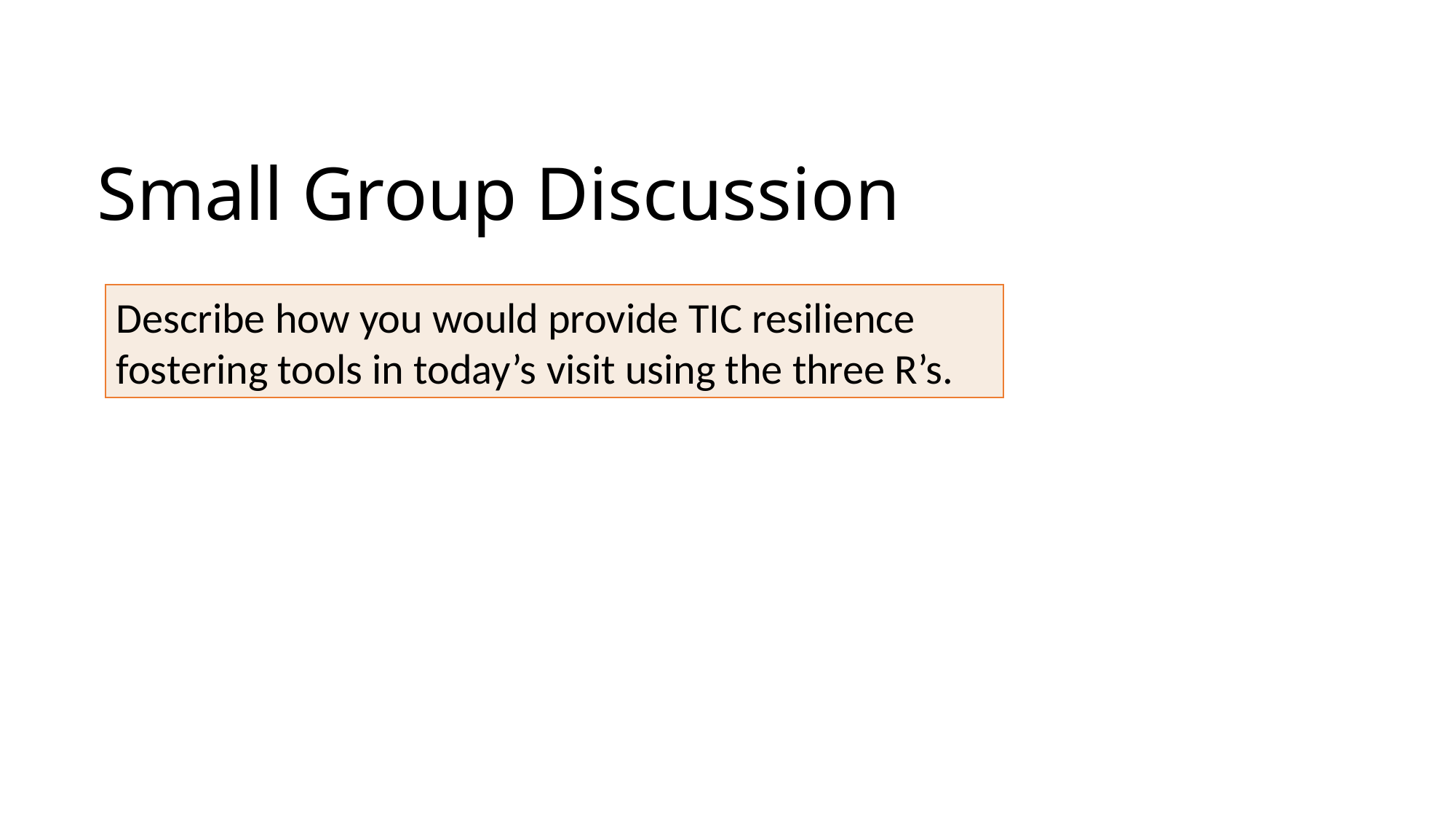

Small Group Discussion
Describe how you would provide TIC resilience fostering tools in today’s visit using the three R’s.

## Slide 16
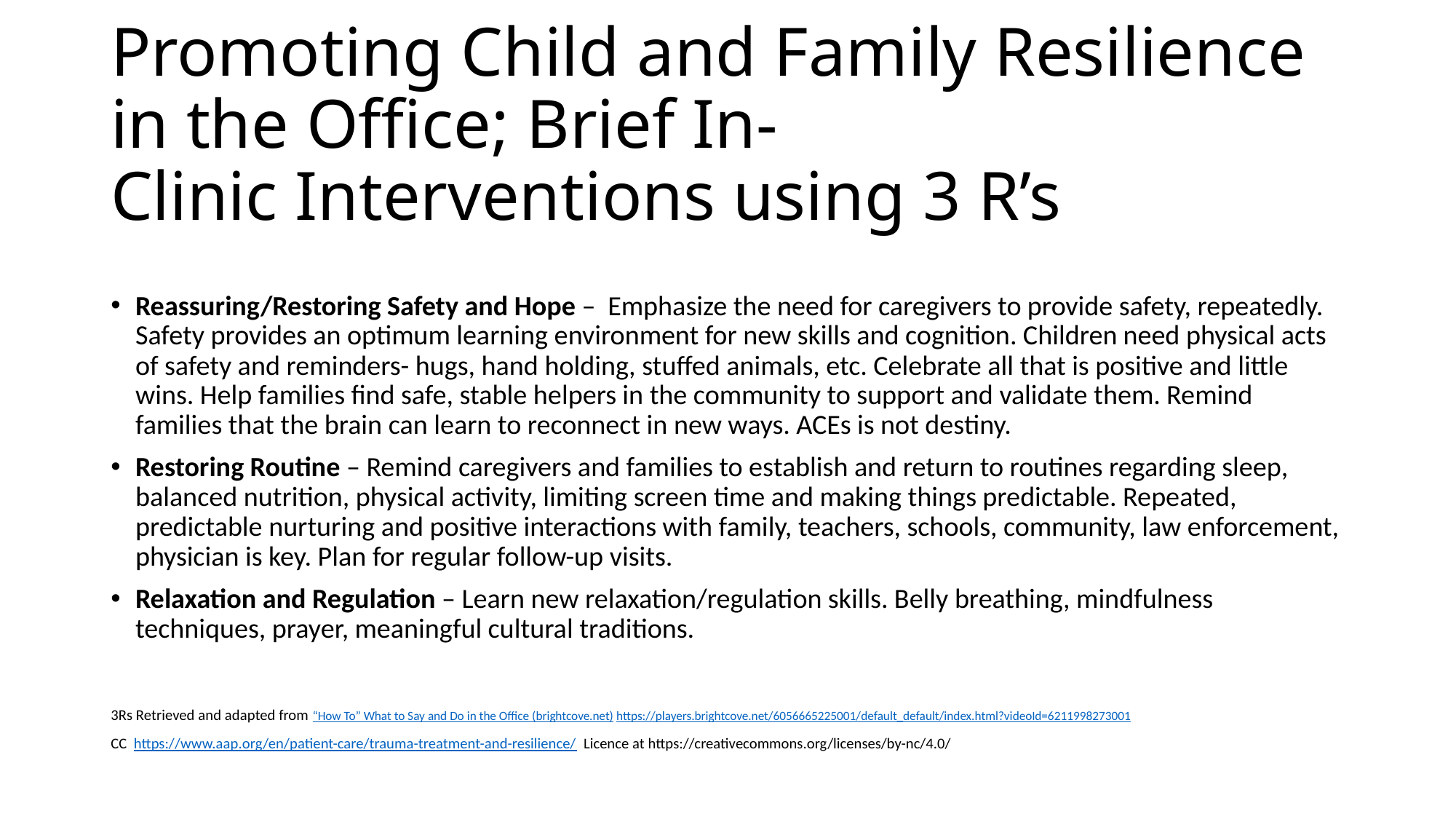

# Promoting Child and Family Resilience in the Office; Brief In-Clinic Interventions using 3 R’s
Reassuring/Restoring Safety and Hope –  Emphasize the need for caregivers to provide safety, repeatedly. Safety provides an optimum learning environment for new skills and cognition. Children need physical acts of safety and reminders- hugs, hand holding, stuffed animals, etc. Celebrate all that is positive and little wins. Help families find safe, stable helpers in the community to support and validate them. Remind families that the brain can learn to reconnect in new ways. ACEs is not destiny.
Restoring Routine – Remind caregivers and families to establish and return to routines regarding sleep, balanced nutrition, physical activity, limiting screen time and making things predictable. Repeated, predictable nurturing and positive interactions with family, teachers, schools, community, law enforcement, physician is key. Plan for regular follow-up visits.
Relaxation and Regulation – Learn new relaxation/regulation skills. Belly breathing, mindfulness techniques, prayer, meaningful cultural traditions.
3Rs Retrieved and adapted from “How To” What to Say and Do in the Office (brightcove.net) https://players.brightcove.net/6056665225001/default_default/index.html?videoId=6211998273001
CC  https://www.aap.org/en/patient-care/trauma-treatment-and-resilience/  Licence at https://creativecommons.org/licenses/by-nc/4.0/

## Slide 17
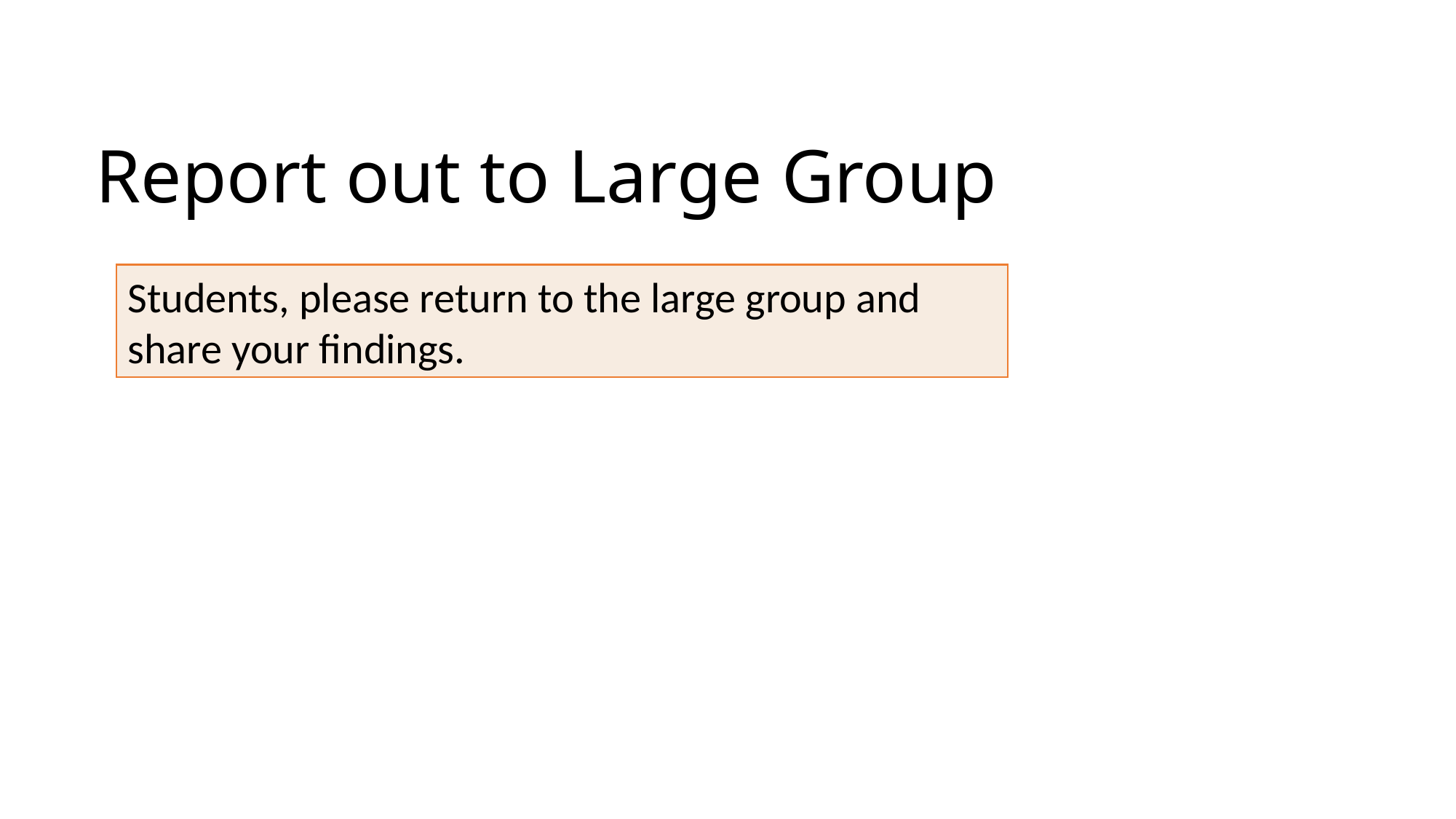

Report out to Large Group
Students, please return to the large group and share your findings.
