# Supplementary material for: Screening for Toxic Stress Response and Buffering Factors: A Case-Based, Trauma-Informed Approach to Health Equity
Source: MedEdPORTAL. 2022 Mar 4;18:11224. doi: 10.15766/mep_2374-8265.11224 (PMC8894523; doi:10.15766/mep_2374-8265.11224)
Supplement: Supplementary file 1 — ACEs and Health Equity Slides.pptxFacilitator Guide.docxFacilitator Slides.pptxStudent Handout.docxPre-, Post-, and 3-Month Follow-up Surveys.docx [file mep_2374-8265.11224-s001.zip › E. Pre-, Post-, and 3-Month Follow-up Surveys.docx]

**Pre-Workshop Survey**

I can define Adverse Childhood Experiences (ACEs).

- Strongly Agree
- Somewhat agree
- Neither agree nor disagree
- Somewhat disagree
- Strongly Disagree

I can describe their impacts on physical, mental and social health.

- Strongly Agree
- Somewhat agree
- Neither agree nor disagree
- Somewhat disagree
- Strongly Disagree

I can describe ACEs as a Health Equity determinant.

- Strongly Agree
- Somewhat agree
- Neither agree nor disagree
- Somewhat disagree
- Strongly Disagree

I can screen for ACEs and Toxic stress in clinical care.

- Strongly Agree
- Somewhat agree
- Neither agree nor disagree
- Somewhat disagree
- Strongly Disagree

I can identify buffering factors in patients.

- Strongly Agree
- Somewhat agree
- Neither agree nor disagree
- Somewhat disagree
- Strongly Disagree

I know of at least 3 brief in-clinic trauma informed resiliency fostering tools to use in the clinic with my patients.

- Strongly Agree
- Somewhat agree
- Neither agree nor disagree
- Somewhat disagree
- Strongly Disagree

I am currently using ACEs and Toxic Stress screening and identification of buffering factors in patient care.

- Yes
- No

**Post- Workshop Survey**

I can define Adverse Childhood Experiences (ACES).

- Strongly Agree
- Somewhat agree
- Neither agree nor disagree
- Somewhat disagree
- Strongly Disagree

I can describe their impacts on physical, mental and social health.

- Strongly Agree
- Somewhat agree
- Neither agree nor disagree
- Somewhat disagree
- Strongly Disagree

I can describe ACEs as a Health Equity determinant.

- Strongly Agree
- Somewhat agree
- Neither agree nor disagree
- Somewhat disagree
- Strongly Disagree

I can screen for ACES and Toxic stress in clinical care.

- Strongly Agree
- Somewhat agree
- Neither agree nor disagree
- Somewhat disagree
- Strongly Disagree

I can identify buffering factors in patients.

- Strongly Agree
- Somewhat agree
- Neither agree nor disagree
- Somewhat disagree
- Strongly Disagree

I know of at least 3 brief in-clinic trauma informed resiliency fostering tools to use in the office with my patients.

- Strongly Agree
- Somewhat agree
- Neither agree nor disagree
- Somewhat disagree
- Strongly Disagree

How likely are you to apply ACEs, toxic stress screening and identification of buffering factors in patient care?

- Likely
- Somewhat likely
- Unlikely

*Qualitative comments:*

1. Describe one way you plan to incorporate ACES and toxic stress screening into patient care.
2. Describe one way you plan to promote buffering and protective factors in patient care.
3. What did you like about this session?
4. What suggestions do you have to improve this session?

**3-Month Follow up Survey**

Thank you for taking our ACES 3-month follow up survey! We are interested in knowing how you have applied the learning objectives of our case-based learning session introducing you to ACEs and Trauma-Informed Care across clinical settings (including but not limited to free clinics, LACE, etc.) Please note, that "patients" refers to **patients of all ages**. We appreciate your time and effort in helping us improve this course.

In the last three months, have you identified protective/buffering factors in patients using THREADS?

- Yes
- No

In the last three months, have you screened for ACEs and Toxic Stress in patients?

- Yes
- No

In the last three months, have you promoted resiliency in the setting of stressors using the 3R's (relaxation/regulation skills, restoring routine, and reassuring safety) with patients?

- Yes
- No

In the last three months, have you incorporated TIC (a patient's history of ACEs, toxic stress and buffering system as part of their clinical encounter) by completing at least one of the following: screening, counseling, or documenting?

- Yes
- No

*Qualitative comments:*

1. In the last three months, what barriers have you faced in incorporating ACEs and toxic stress screening in a patient care setting?
2. In the last three months, what barriers have you faced in promoting resilience factors in a patient care setting?
