# Supplementary material for: Screening for Toxic Stress Response and Buffering Factors: A Case-Based, Trauma-Informed Approach to Health Equity
Source: MedEdPORTAL. 2022 Mar 4;18:11224. doi: 10.15766/mep_2374-8265.11224 (PMC8894523; doi:10.15766/mep_2374-8265.11224)
Supplement: Supplementary file 1 — ACEs and Health Equity Slides.pptxFacilitator Guide.docxFacilitator Slides.pptxStudent Handout.docxPre-, Post-, and 3-Month Follow-up Surveys.docx [file mep_2374-8265.11224-s001.zip › A. ACEs and Health Equity Slides.pptx]

## Slide 1
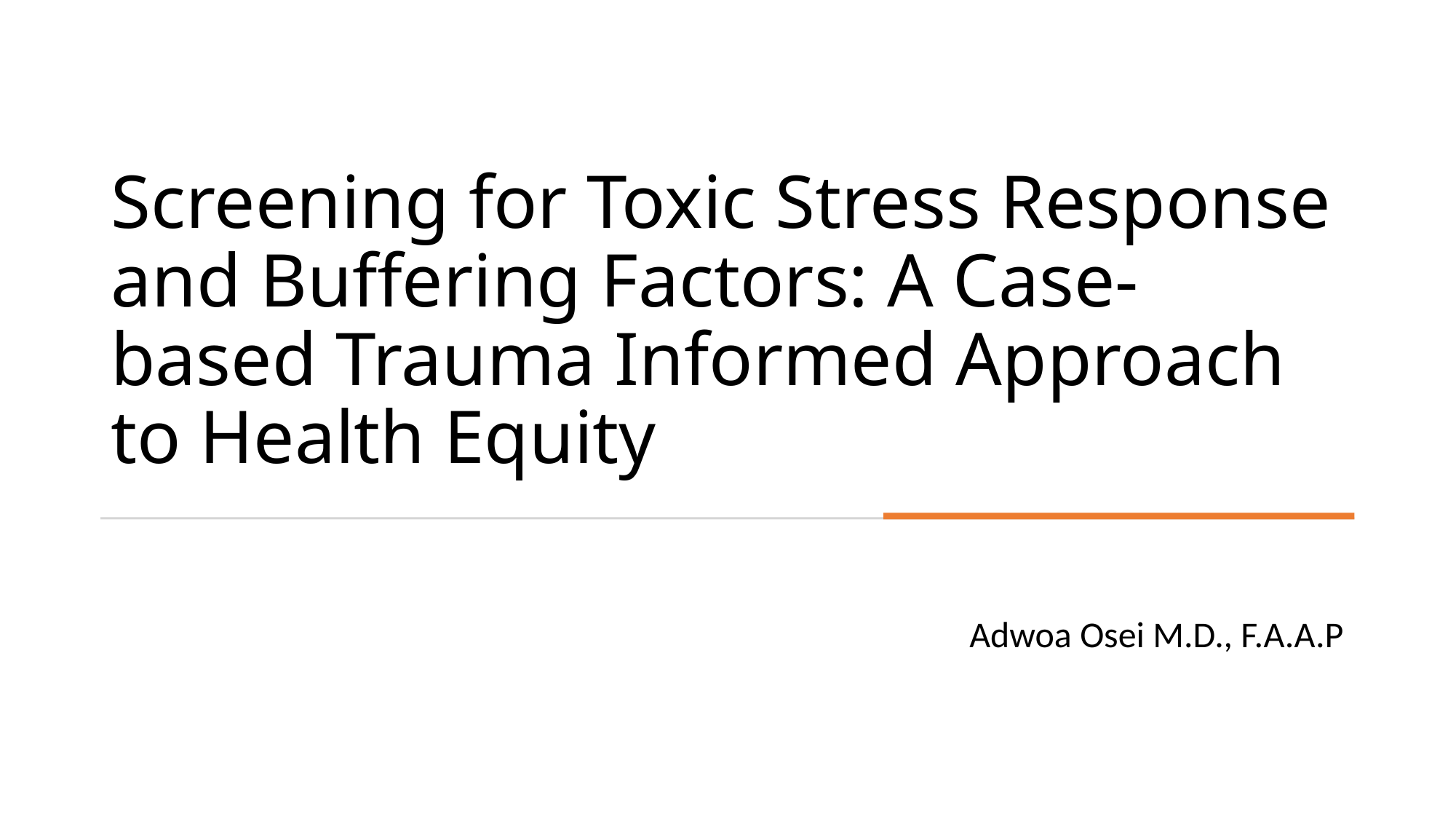

# Screening for Toxic Stress Response and Buffering Factors: A Case-based Trauma Informed Approach to Health Equity
Adwoa Osei M.D., F.A.A.P

## Slide 2
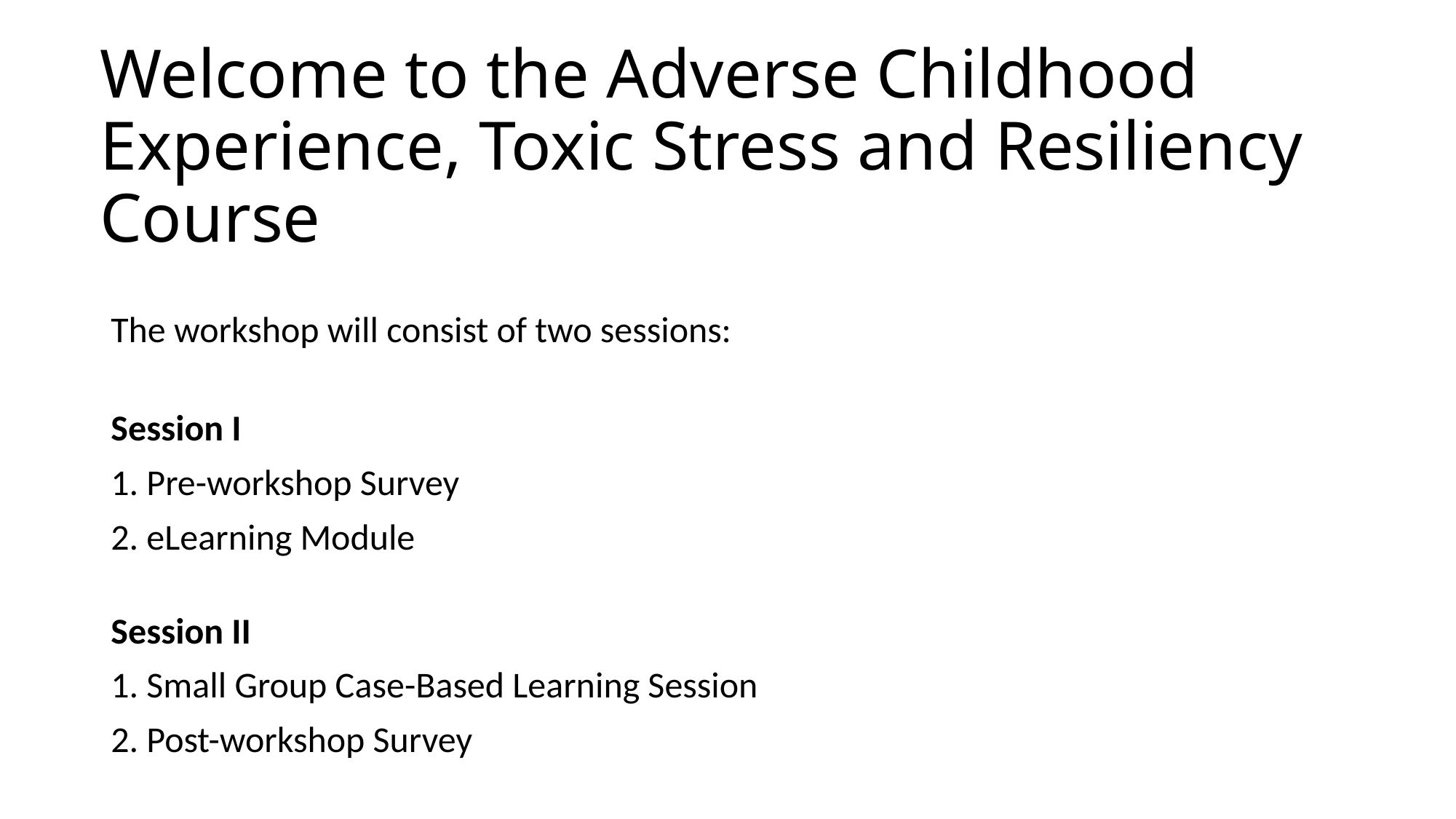

# Welcome to the Adverse Childhood Experience, Toxic Stress and Resiliency Course
The workshop will consist of two sessions:
Session I
1. Pre-workshop Survey
2. eLearning Module
Session II
1. Small Group Case-Based Learning Session
2. Post-workshop Survey

## Slide 3
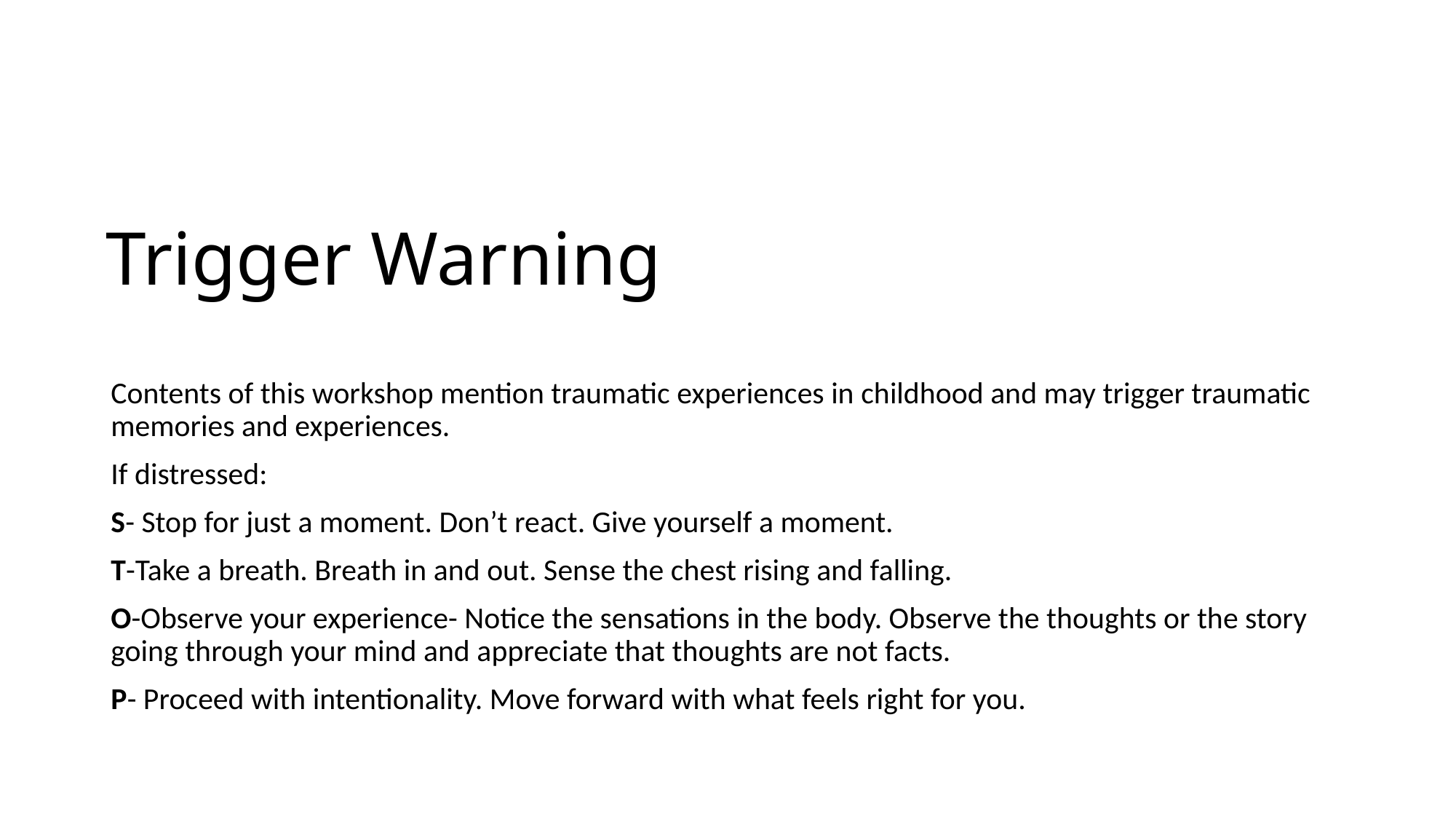

# Trigger Warning
Contents of this workshop mention traumatic experiences in childhood and may trigger traumatic memories and experiences.
If distressed:
S- Stop for just a moment. Don’t react. Give yourself a moment.
T-Take a breath. Breath in and out. Sense the chest rising and falling.
O-Observe your experience- Notice the sensations in the body. Observe the thoughts or the story going through your mind and appreciate that thoughts are not facts.
P- Proceed with intentionality. Move forward with what feels right for you.

## Slide 4
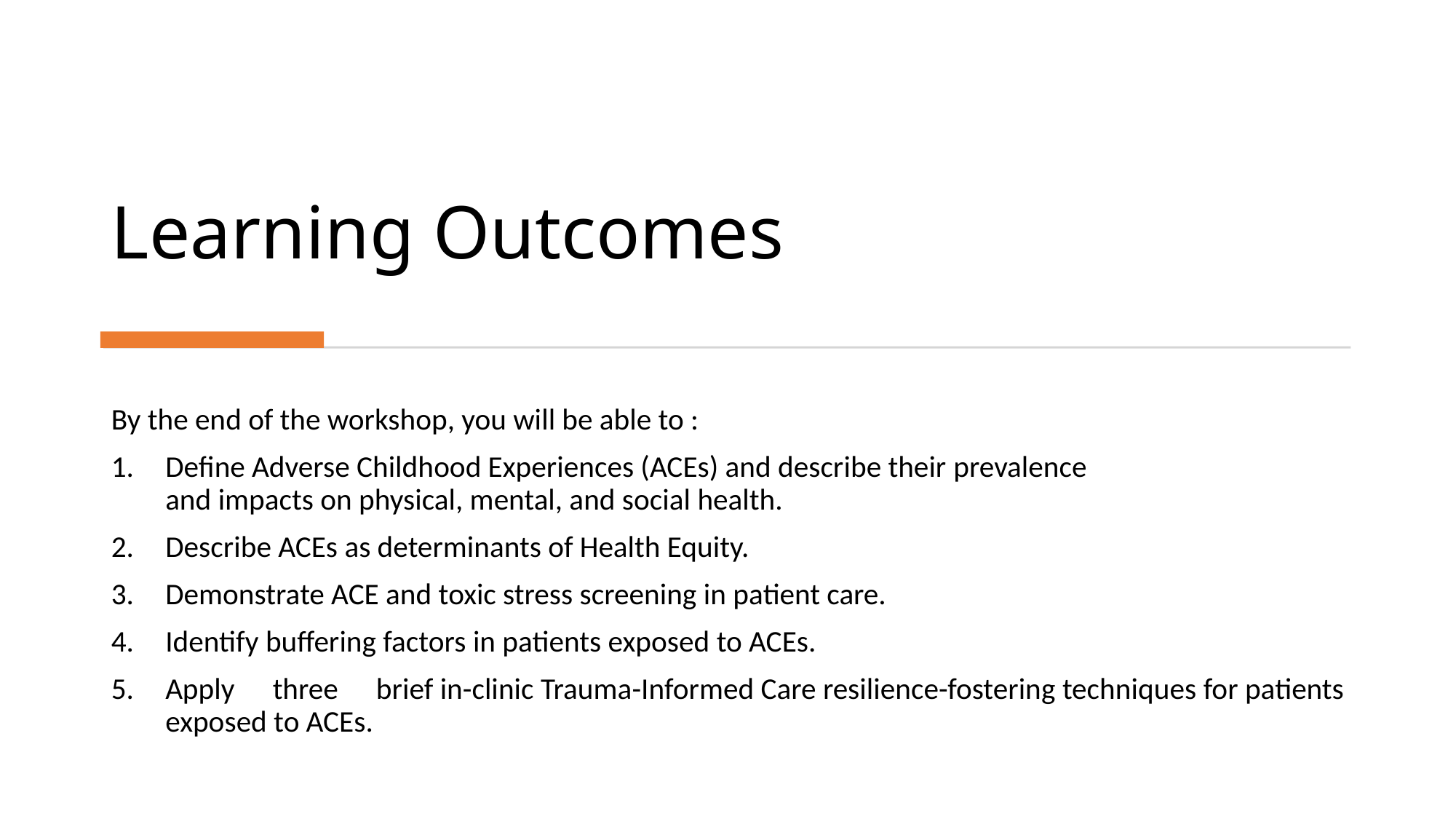

# Learning Outcomes
By the end of the workshop, you will be able to :
Define Adverse Childhood Experiences (ACEs) and describe their prevalence and impacts on physical, mental, and social health.
Describe ACEs as determinants of Health Equity.
Demonstrate ACE and toxic stress screening in patient care.
Identify buffering factors in patients exposed to ACEs.
Apply three brief in-clinic Trauma-Informed Care resilience-fostering techniques for patients exposed to ACEs.

## Slide 5
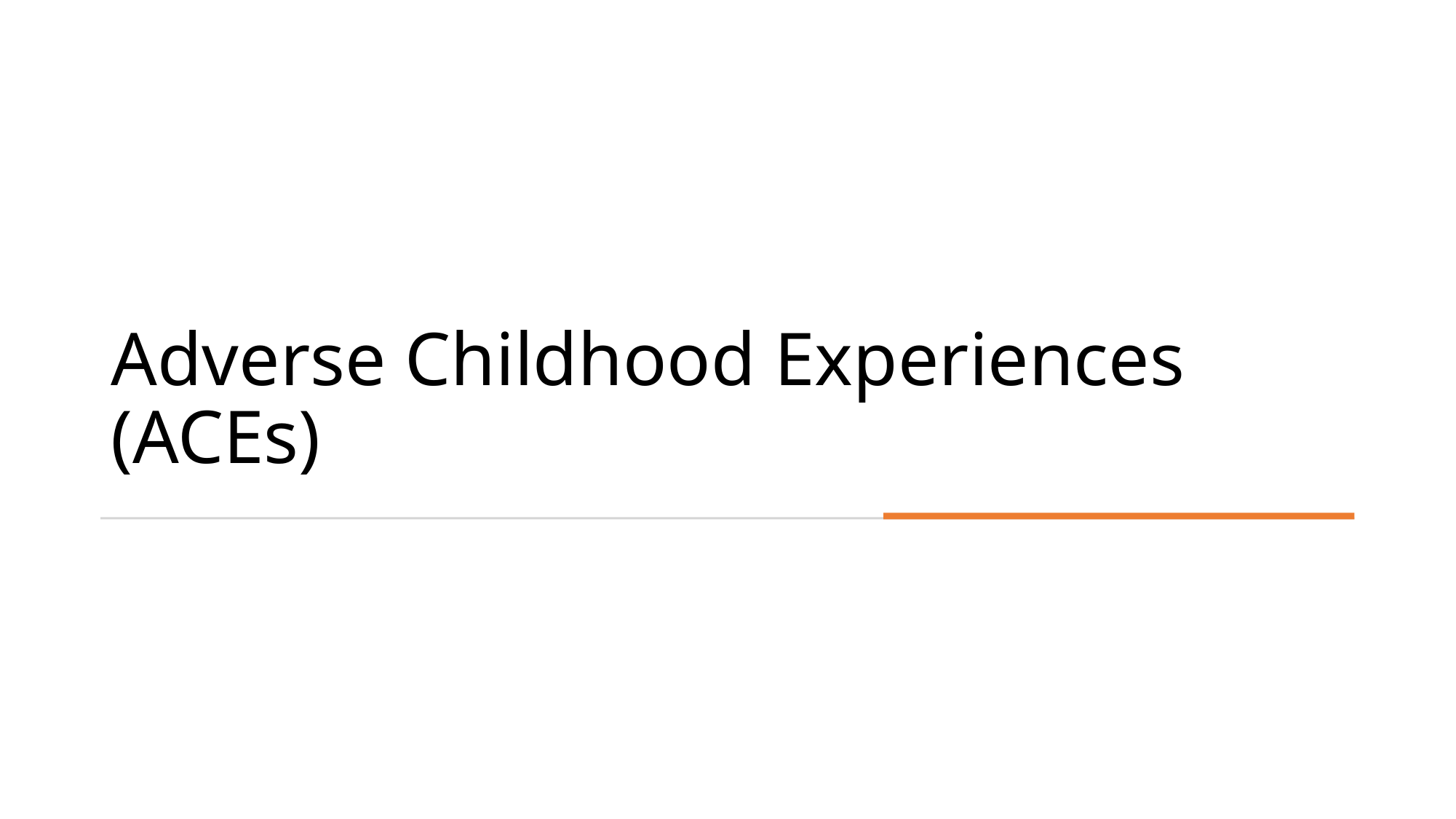

# Adverse Childhood Experiences (ACEs)

## Slide 6
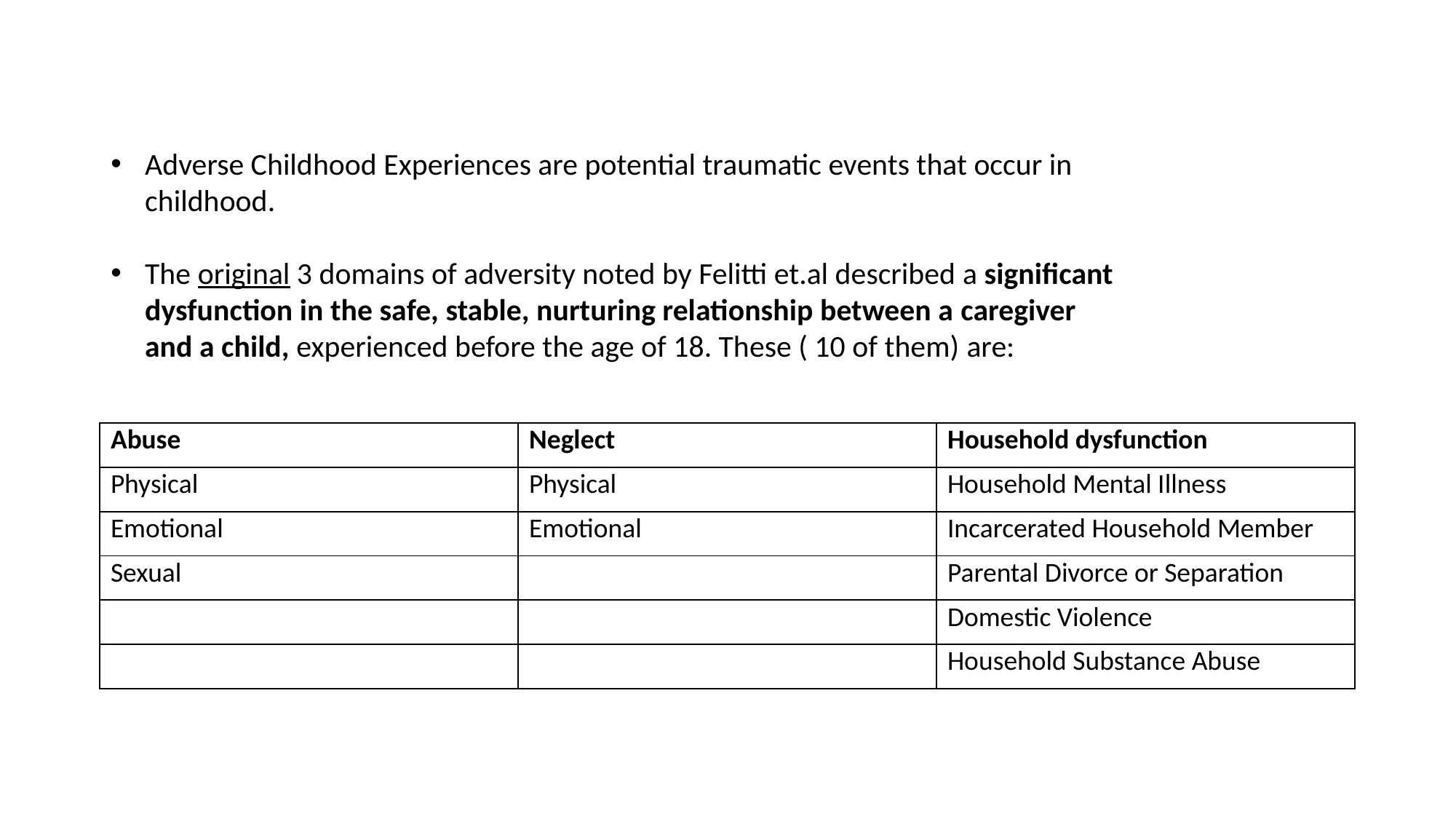

Adverse Childhood Experiences are potential traumatic events that occur in childhood.
The original 3 domains of adversity noted by Felitti et.al described a significant dysfunction in the safe, stable, nurturing relationship between a caregiver and a child, experienced before the age of 18. These ( 10 of them) are:
| Abuse | Neglect | Household dysfunction |
| --- | --- | --- |
| Physical | Physical | Household Mental Illness |
| Emotional | Emotional | Incarcerated Household Member |
| Sexual | | Parental Divorce or Separation |
| | | Domestic Violence |
| | | Household Substance Abuse |

## Slide 7
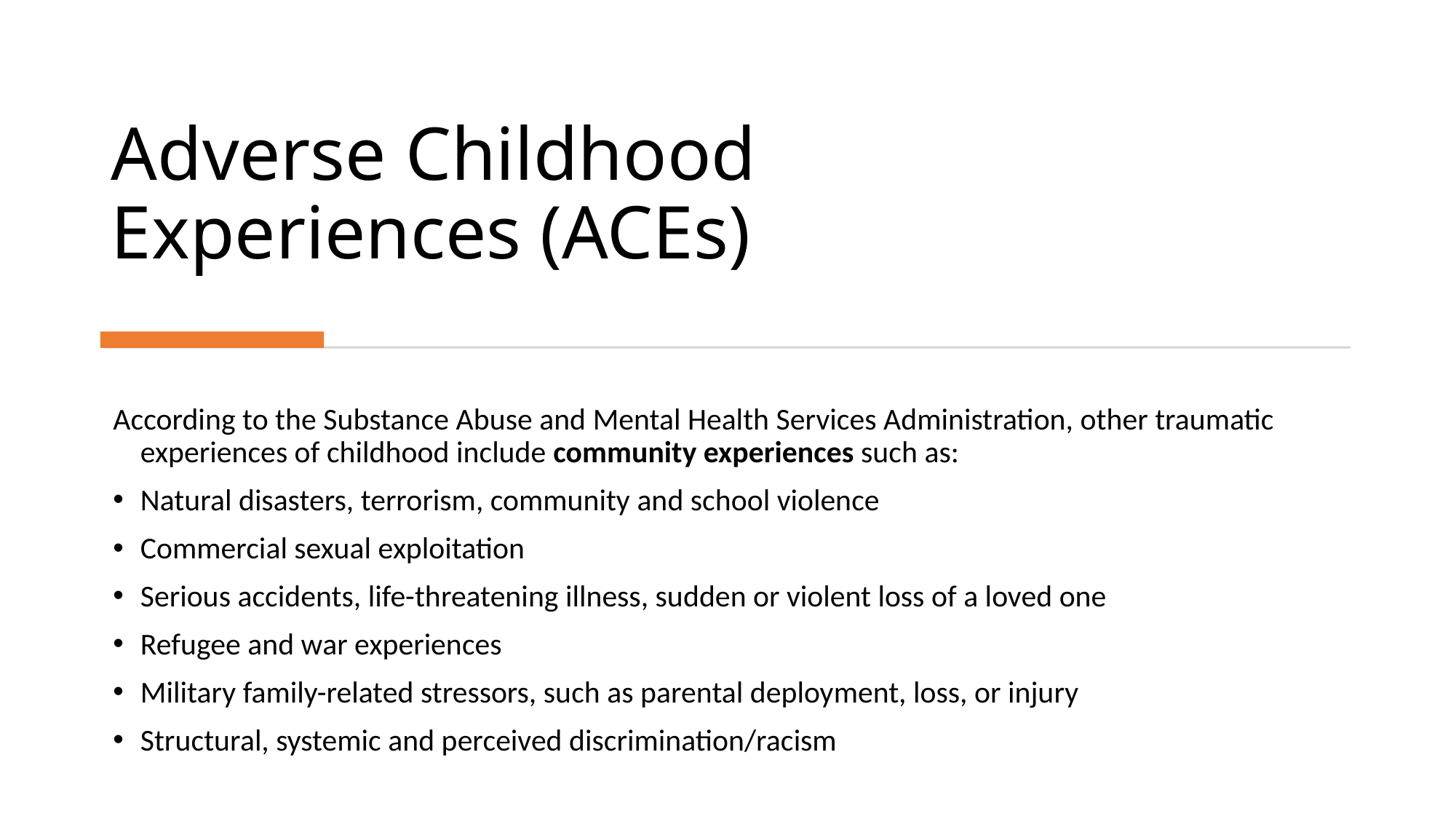

# Adverse Childhood Experiences (ACEs)
According to the Substance Abuse and Mental Health Services Administration, other traumatic experiences of childhood include community experiences such as:
Natural disasters, terrorism, community and school violence
Commercial sexual exploitation
Serious accidents, life-threatening illness, sudden or violent loss of a loved one
Refugee and war experiences
Military family-related stressors, such as parental deployment, loss, or injury
Structural, systemic and perceived discrimination/racism

## Slide 8
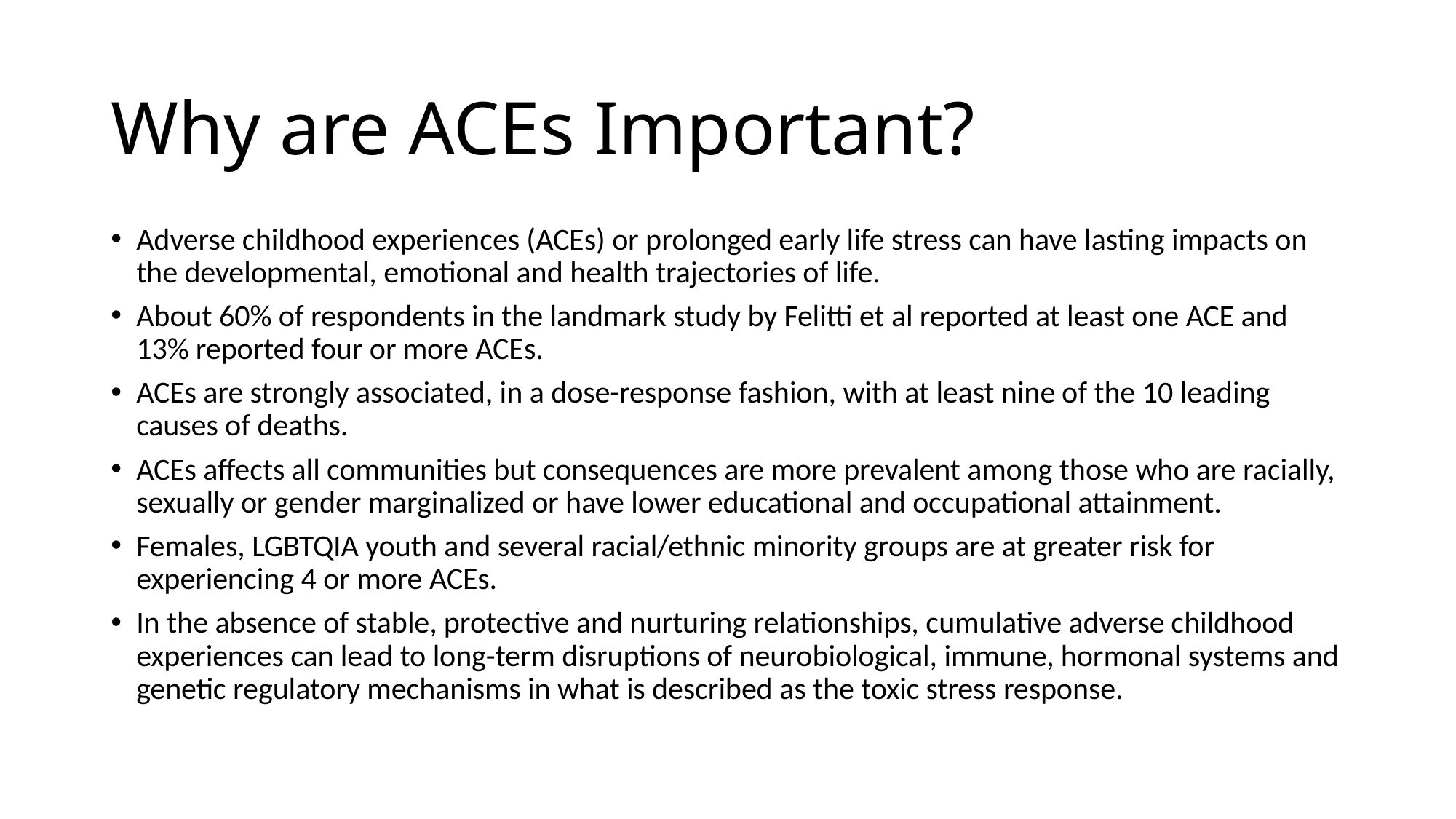

# Why are ACEs Important?
Adverse childhood experiences (ACEs) or prolonged early life stress can have lasting impacts on the developmental, emotional and health trajectories of life.
About 60% of respondents in the landmark study by Felitti et al reported at least one ACE and 13% reported four or more ACEs.
ACEs are strongly associated, in a dose-response fashion, with at least nine of the 10 leading causes of deaths.
ACEs affects all communities but consequences are more prevalent among those who are racially, sexually or gender marginalized or have lower educational and occupational attainment.
Females, LGBTQIA youth and several racial/ethnic minority groups are at greater risk for experiencing 4 or more ACEs.
In the absence of stable, protective and nurturing relationships, cumulative adverse childhood experiences can lead to long-term disruptions of neurobiological, immune, hormonal systems and genetic regulatory mechanisms in what is described as the toxic stress response.

## Slide 9
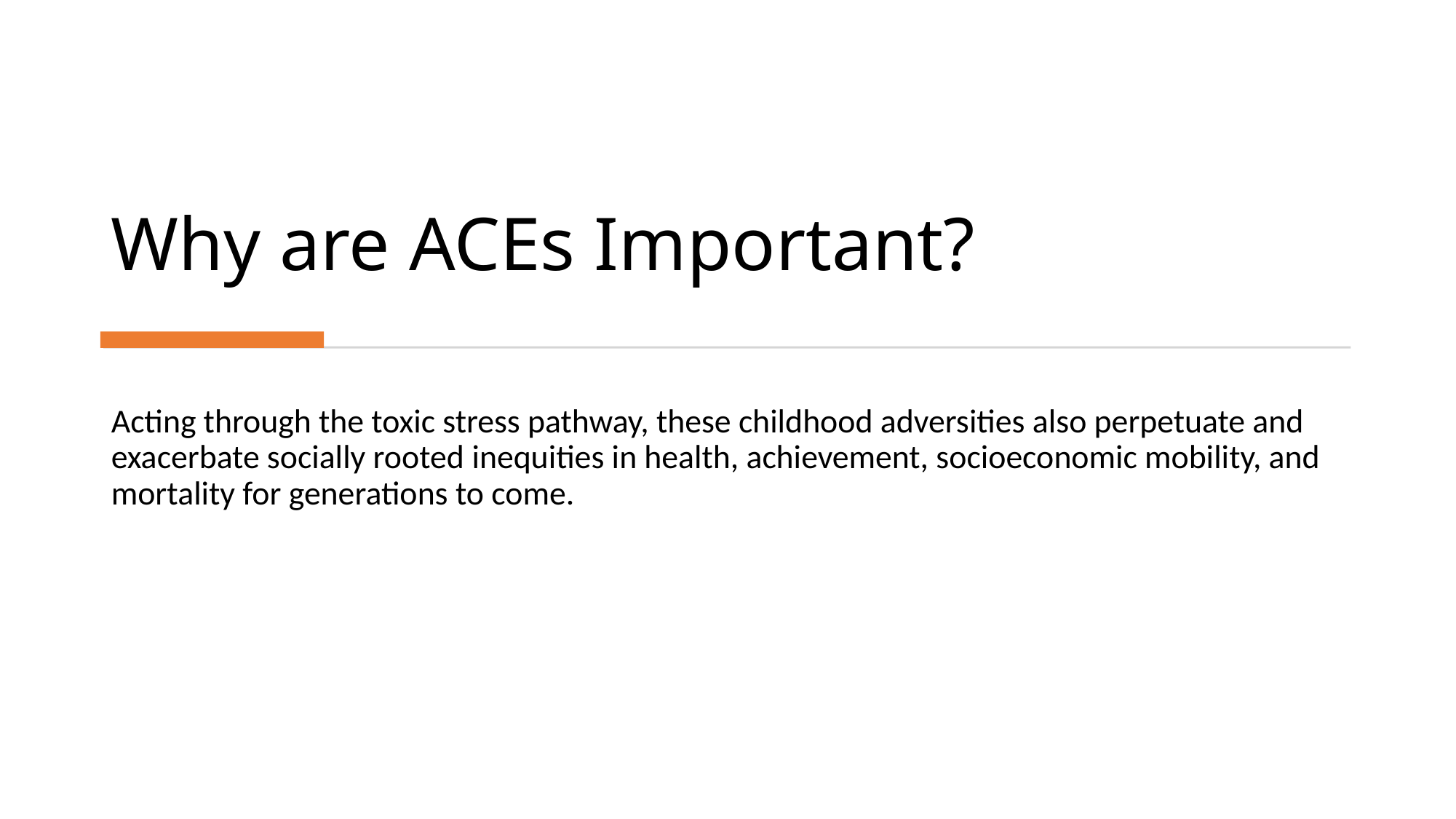

# Why are ACEs Important?
Acting through the toxic stress pathway, these childhood adversities also perpetuate and exacerbate socially rooted inequities in health, achievement, socioeconomic mobility, and mortality for generations to come.

## Slide 10
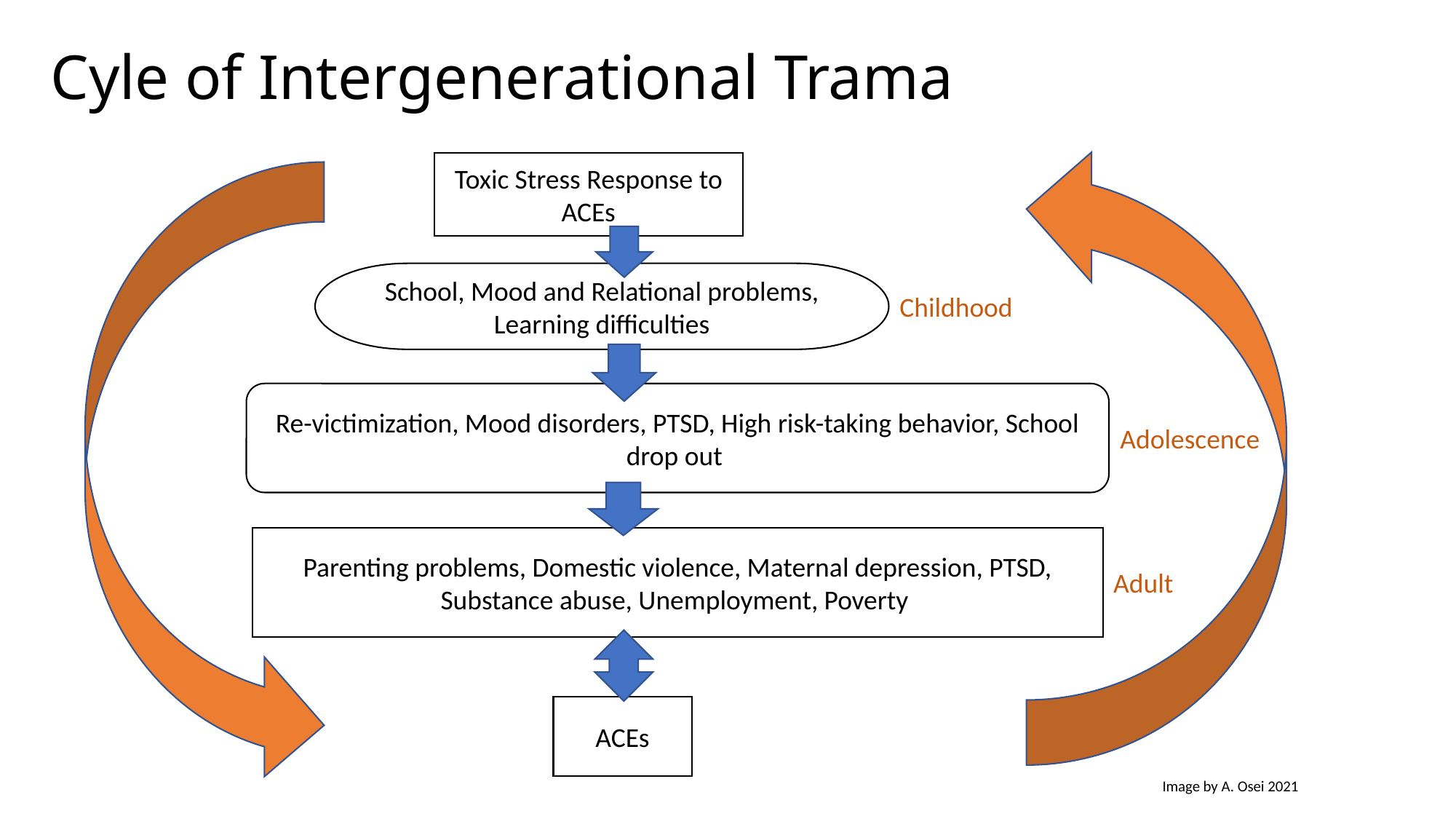

# Cyle of Intergenerational Trama
Toxic Stress Response to ACEs
School, Mood and Relational problems, Learning difficulties
Childhood
Re-victimization, Mood disorders, PTSD, High risk-taking behavior, School drop out
Adolescence
Parenting problems, Domestic violence, Maternal depression, PTSD, Substance abuse, Unemployment, Poverty
Adult
ACEs
Image by A. Osei 2021
Click to add te

## Slide 11
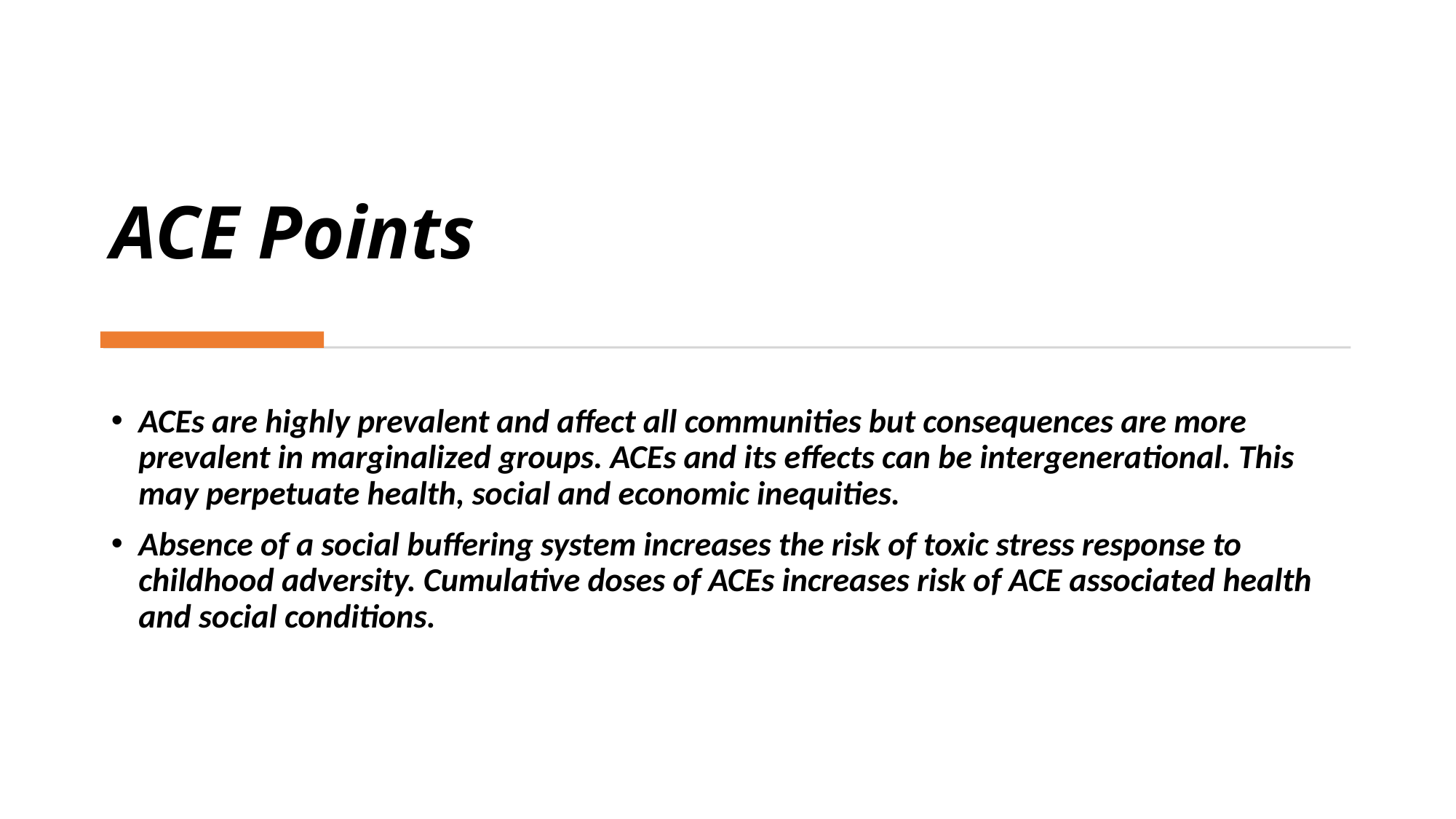

# ACE Points
ACEs are highly prevalent and affect all communities but consequences are more prevalent in marginalized groups. ACEs and its effects can be intergenerational. This may perpetuate health, social and economic inequities.
Absence of a social buffering system increases the risk of toxic stress response to childhood adversity. Cumulative doses of ACEs increases risk of ACE associated health and social conditions.

## Slide 12
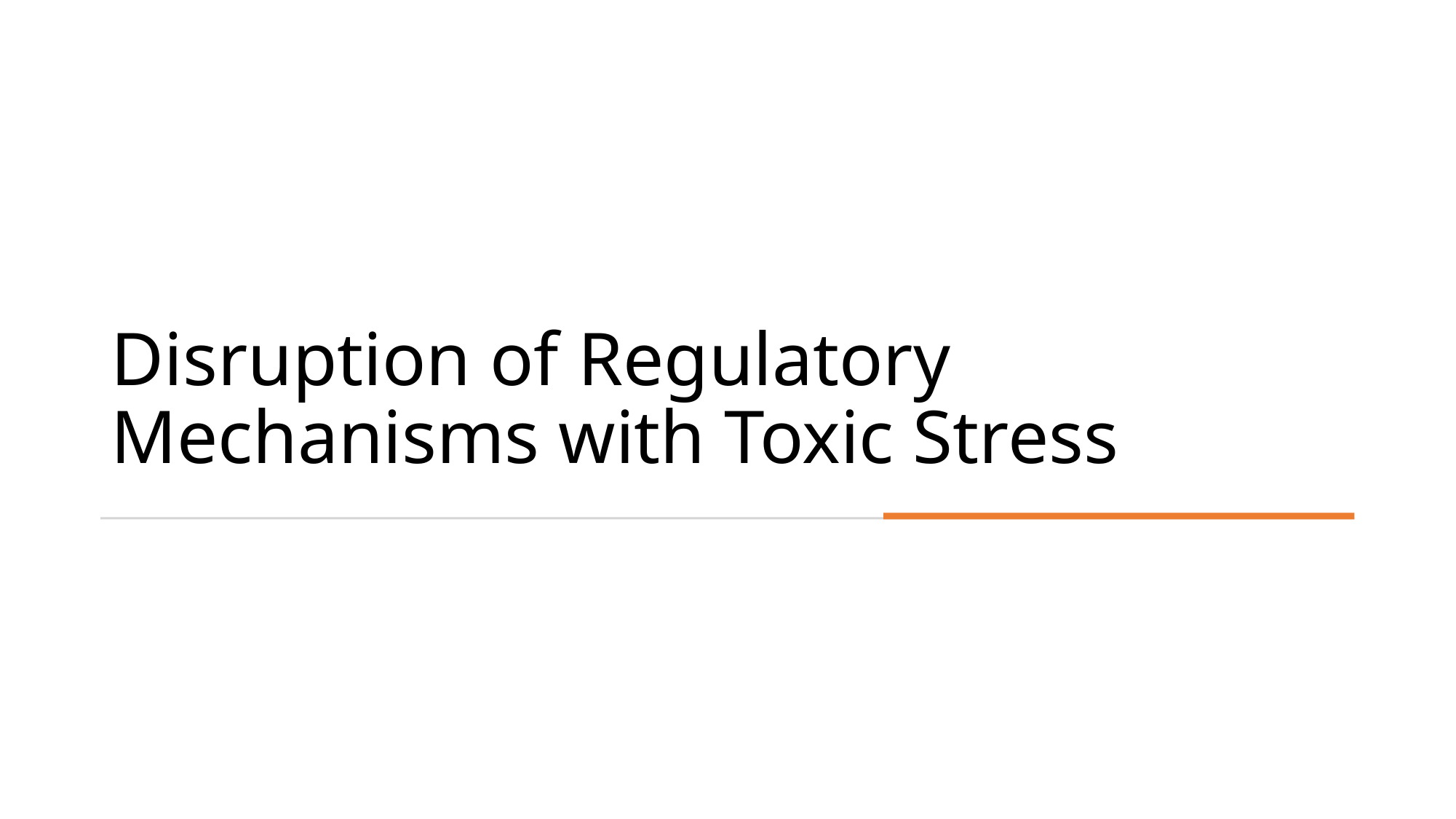

# Disruption of Regulatory Mechanisms with Toxic Stress

## Slide 13
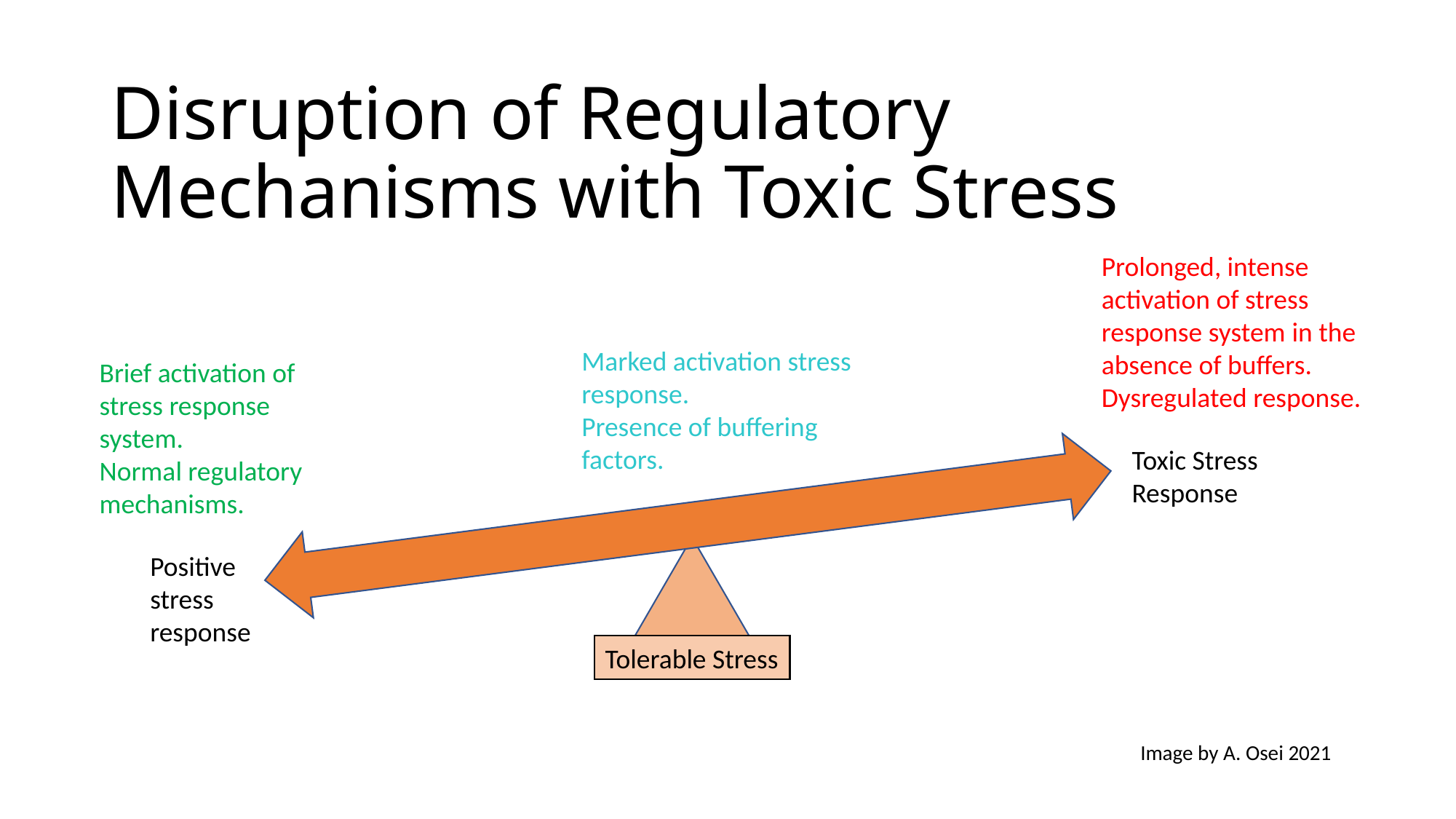

# Disruption of Regulatory Mechanisms with Toxic Stress
Prolonged, intense activation of stress response system in the absence of buffers.
Dysregulated response.
Marked activation stress response.
Presence of buffering factors.
Brief activation of stress response system.
Normal regulatory mechanisms.
Toxic Stress Response
Positive stress response
Tolerable Stress
Image by A. Osei 2021

## Slide 14
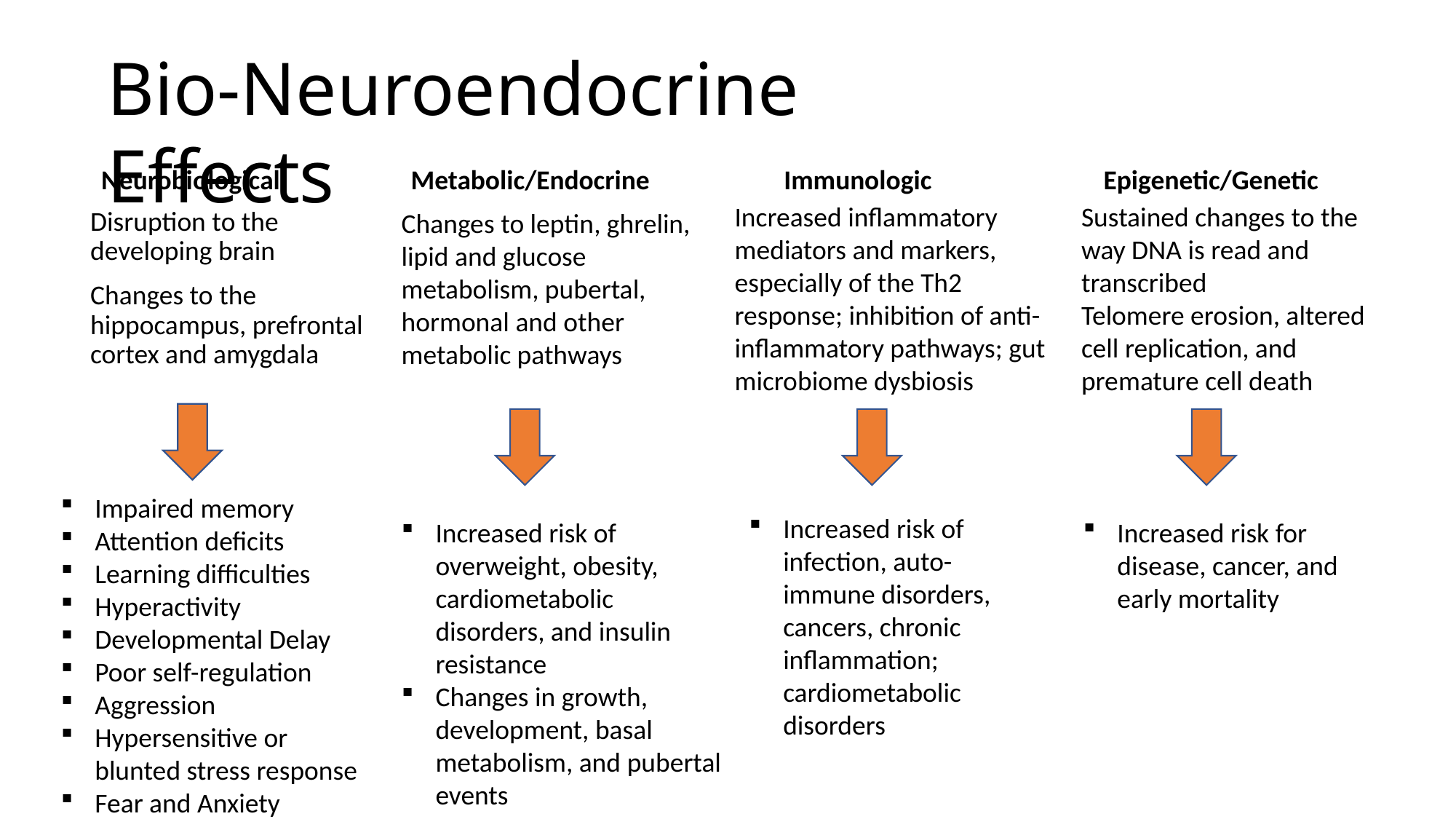

Bio-Neuroendocrine Effects
Neurobiological
Metabolic/Endocrine
Epigenetic/Genetic
Immunologic
Increased inflammatory mediators and markers, especially of the Th2 response; inhibition of anti-inflammatory pathways; gut microbiome dysbiosis
Sustained changes to the way DNA is read and transcribed
Telomere erosion, altered cell replication, and premature cell death
Changes to leptin, ghrelin, lipid and glucose metabolism, pubertal, hormonal and other metabolic pathways
Disruption to the developing brain
Changes to the hippocampus, prefrontal cortex and amygdala
Impaired memory
Attention deficits
Learning difficulties
Hyperactivity
Developmental Delay
Poor self-regulation
Aggression
Hypersensitive or blunted stress response
Fear and Anxiety
Increased risk of infection, auto-immune disorders, cancers, chronic inflammation; cardiometabolic disorders
Increased risk for disease, cancer, and early mortality
Increased risk of overweight, obesity, cardiometabolic disorders, and insulin resistance
Changes in growth, development, basal metabolism, and pubertal events

## Slide 15
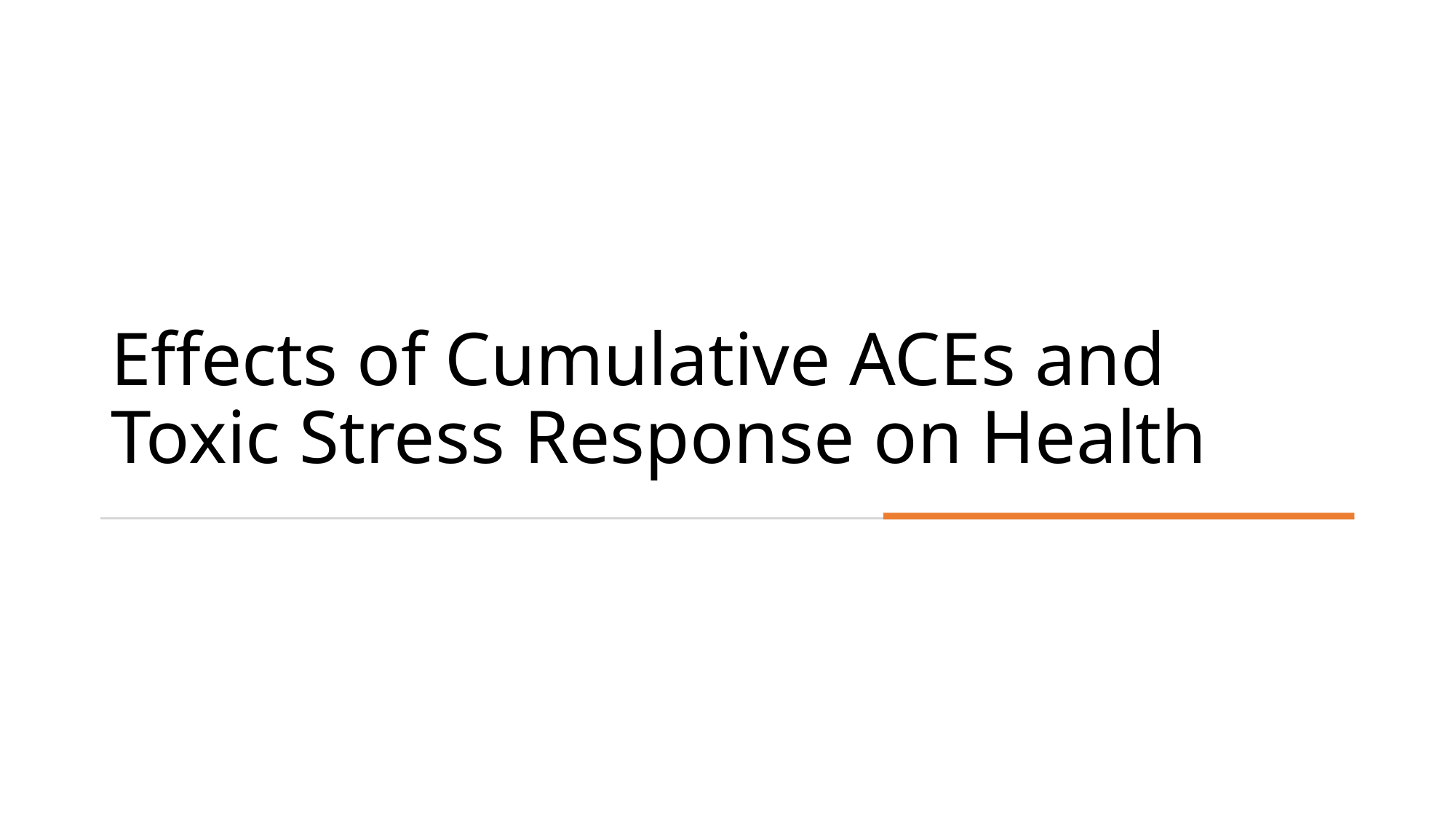

# Effects of Cumulative ACEs and Toxic Stress Response on Health

## Slide 16
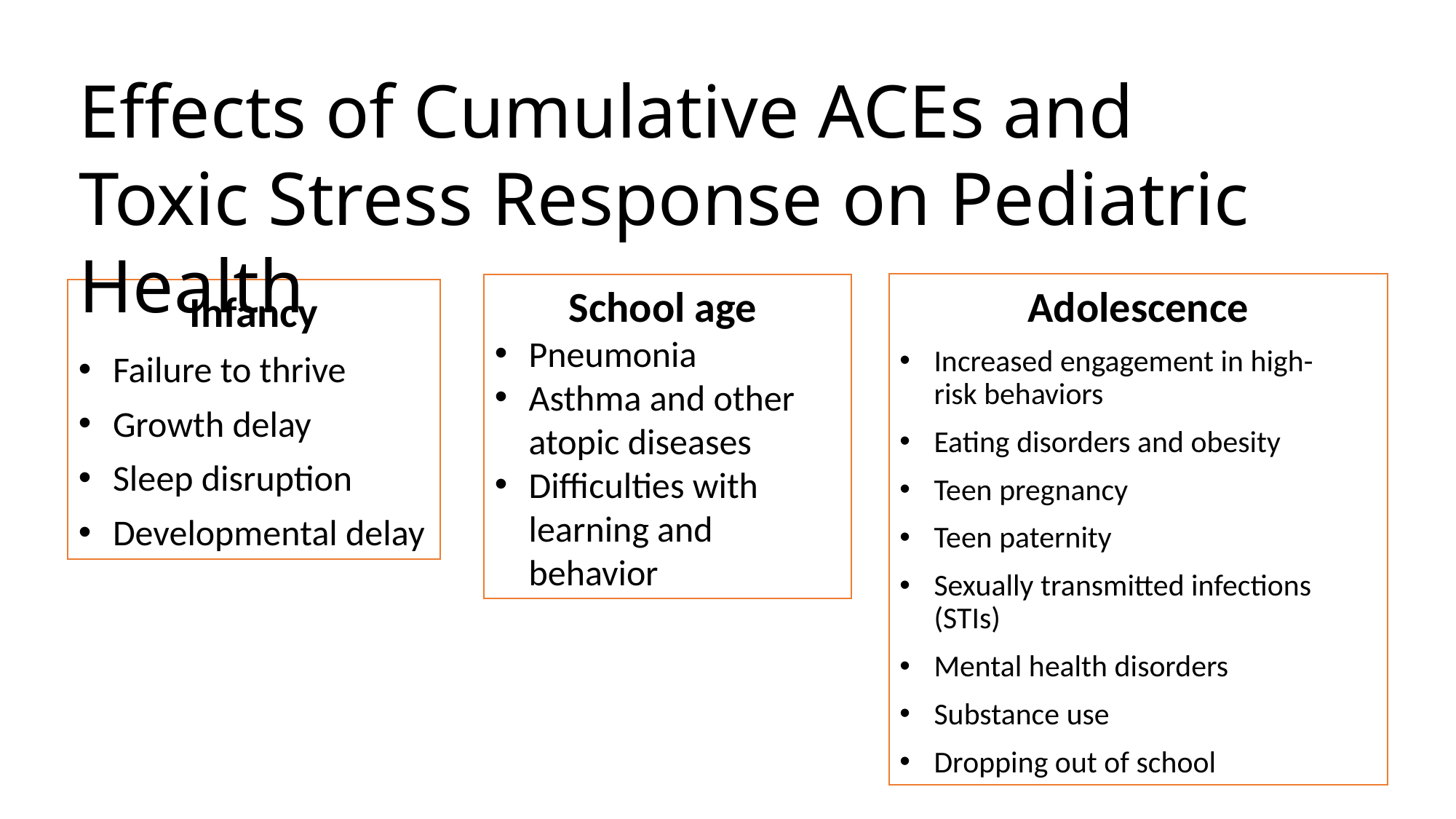

Effects of Cumulative ACEs and Toxic Stress Response on Pediatric Health
Adolescence
Increased engagement in high-risk behaviors
Eating disorders and obesity
Teen pregnancy
Teen paternity
Sexually transmitted infections (STIs)
Mental health disorders
Substance use
Dropping out of school
School age
Pneumonia
Asthma and other atopic diseases
Difficulties with learning and behavior
Infancy
Failure to thrive
Growth delay
Sleep disruption
Developmental delay

## Slide 17
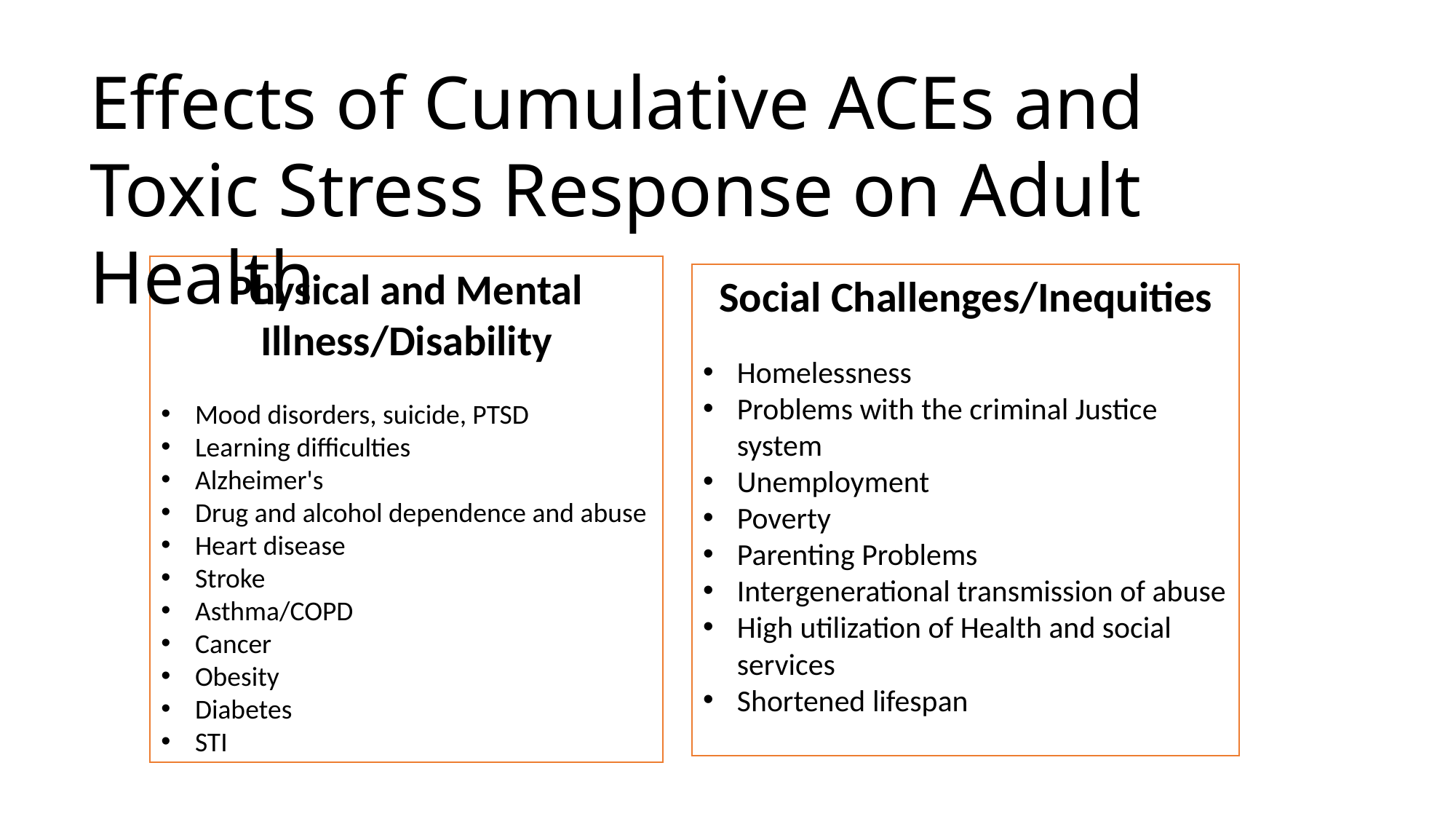

Effects of Cumulative ACEs and Toxic Stress Response on Adult Health
Physical and Mental Illness/Disability
Mood disorders, suicide, PTSD
Learning difficulties
Alzheimer's
Drug and alcohol dependence and abuse
Heart disease
Stroke
Asthma/COPD
Cancer
Obesity
Diabetes
STI
Social Challenges/Inequities
Homelessness
Problems with the criminal Justice system
Unemployment
Poverty
Parenting Problems
Intergenerational transmission of abuse
High utilization of Health and social services
Shortened lifespan

## Slide 18
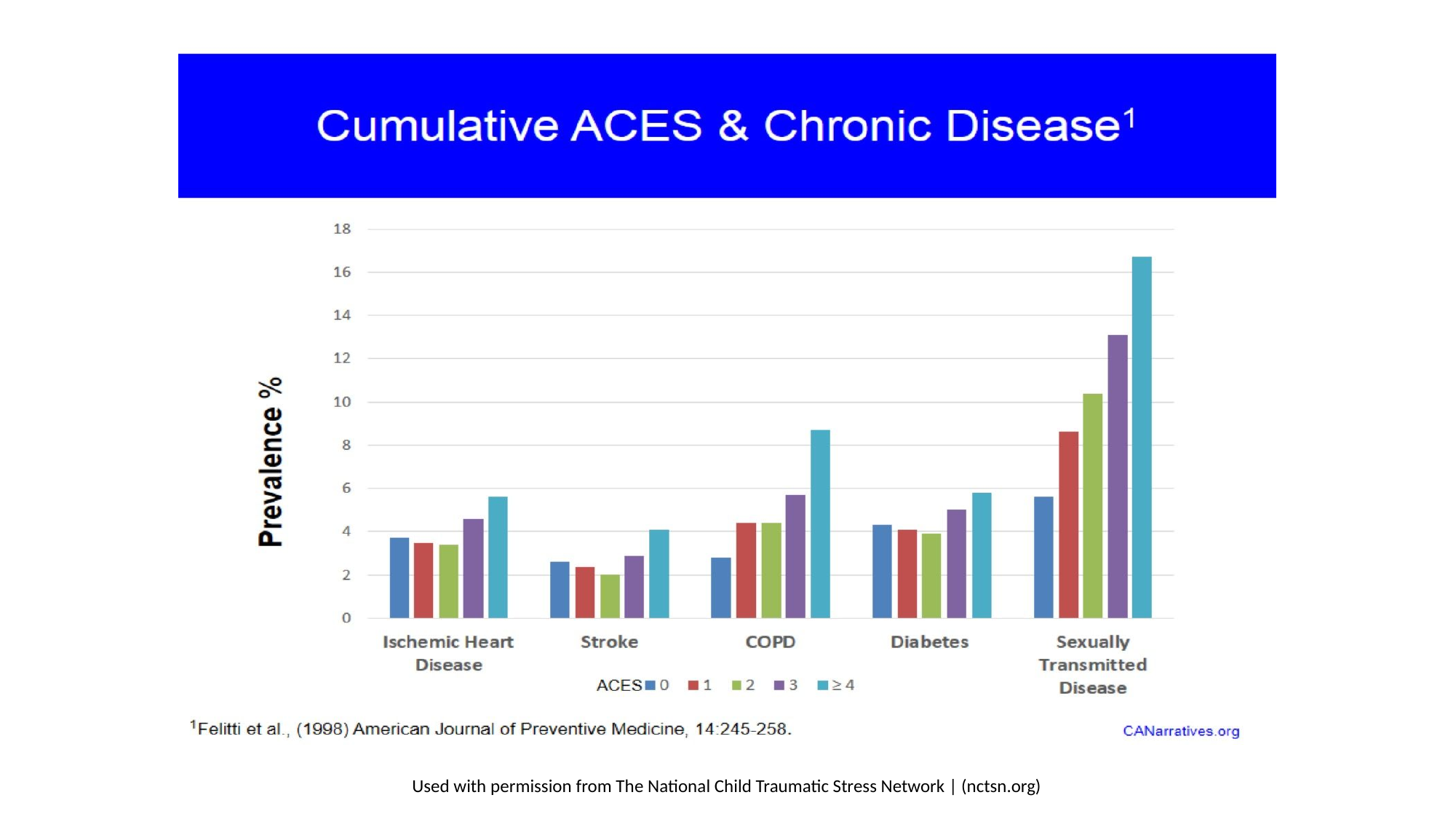

Used with permission from The National Child Traumatic Stress Network | (nctsn.org)

## Slide 19
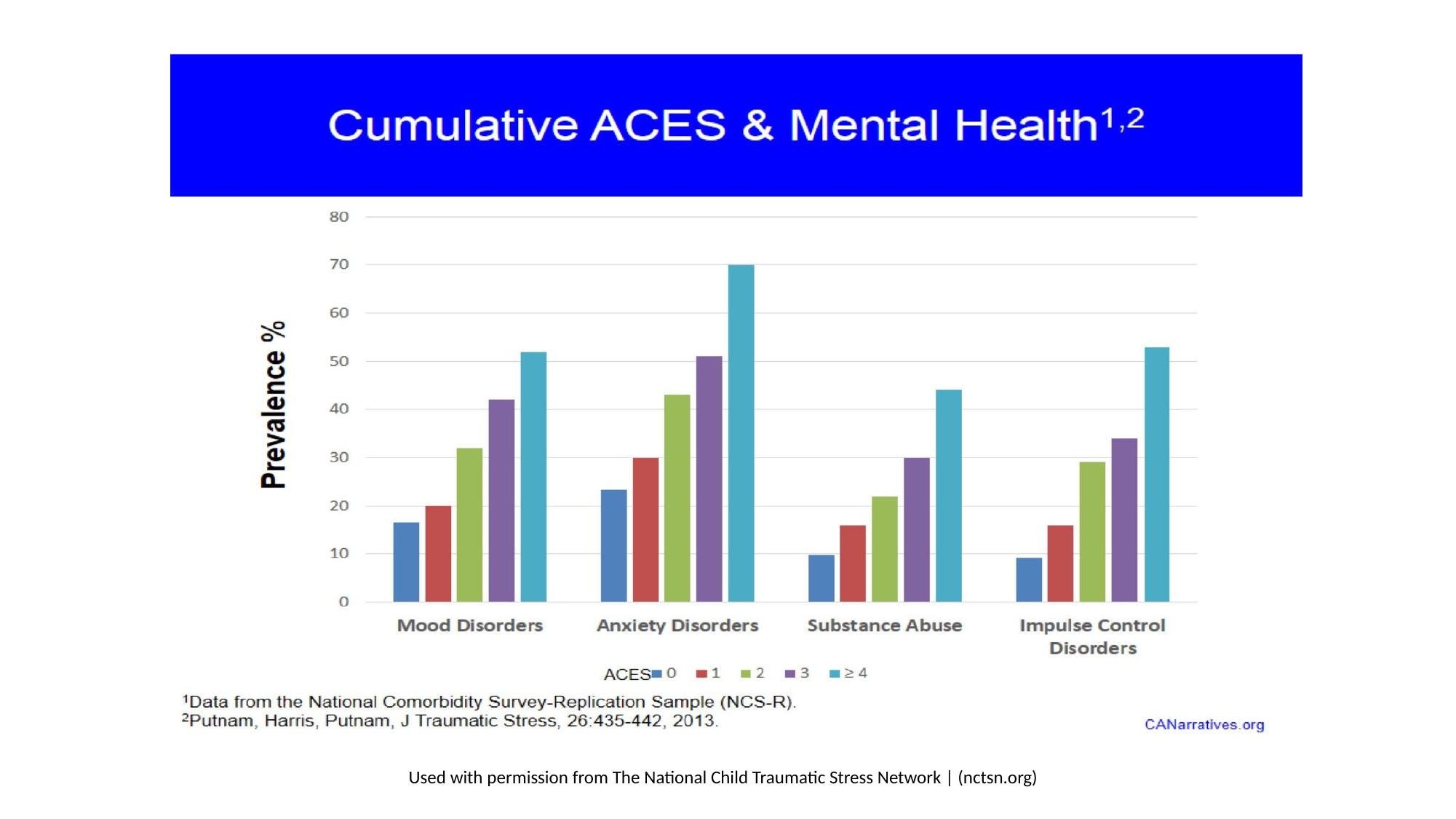

Used with permission from The National Child Traumatic Stress Network | (nctsn.org)

## Slide 20
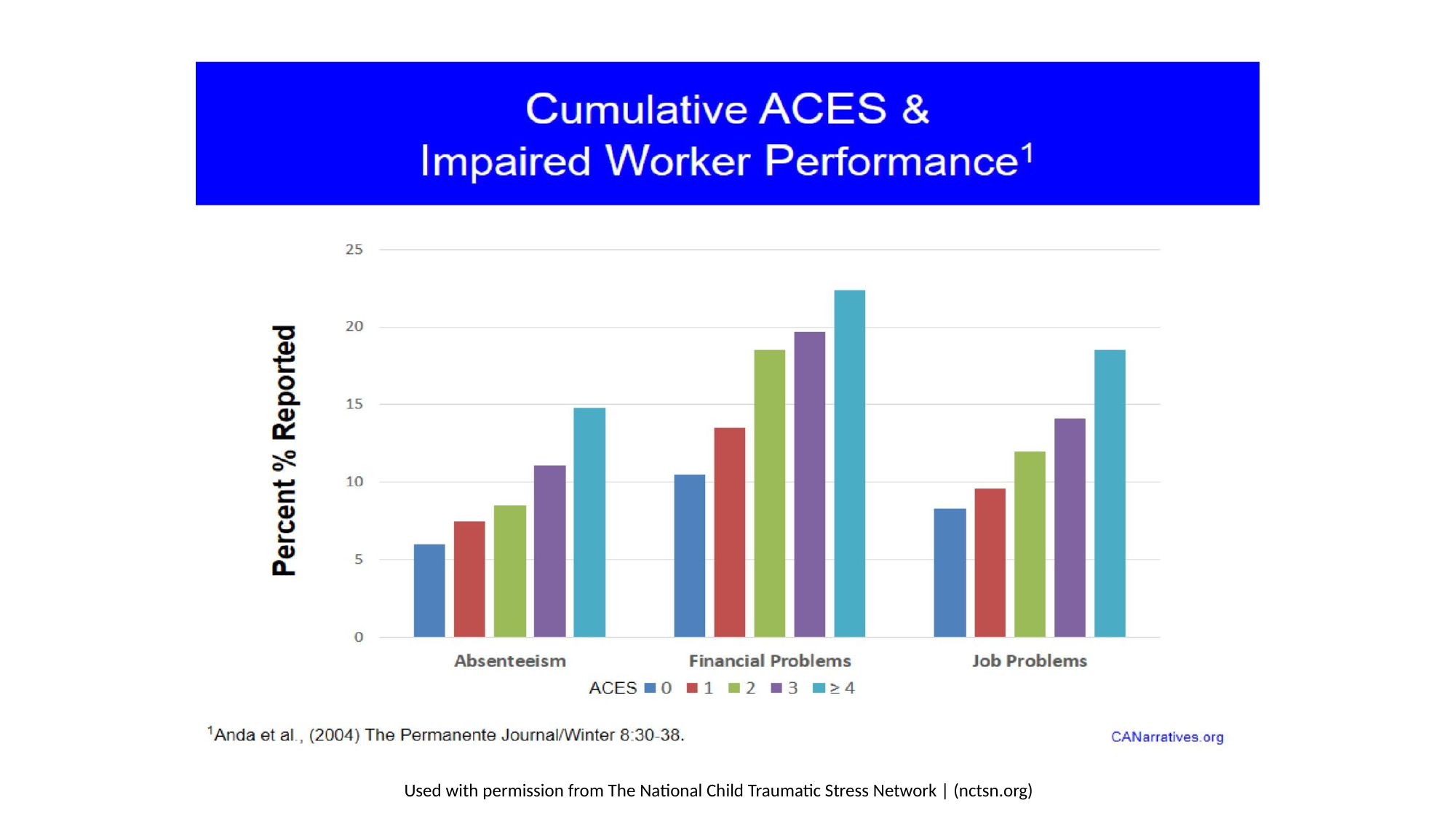

Used with permission from The National Child Traumatic Stress Network | (nctsn.org)

## Slide 21
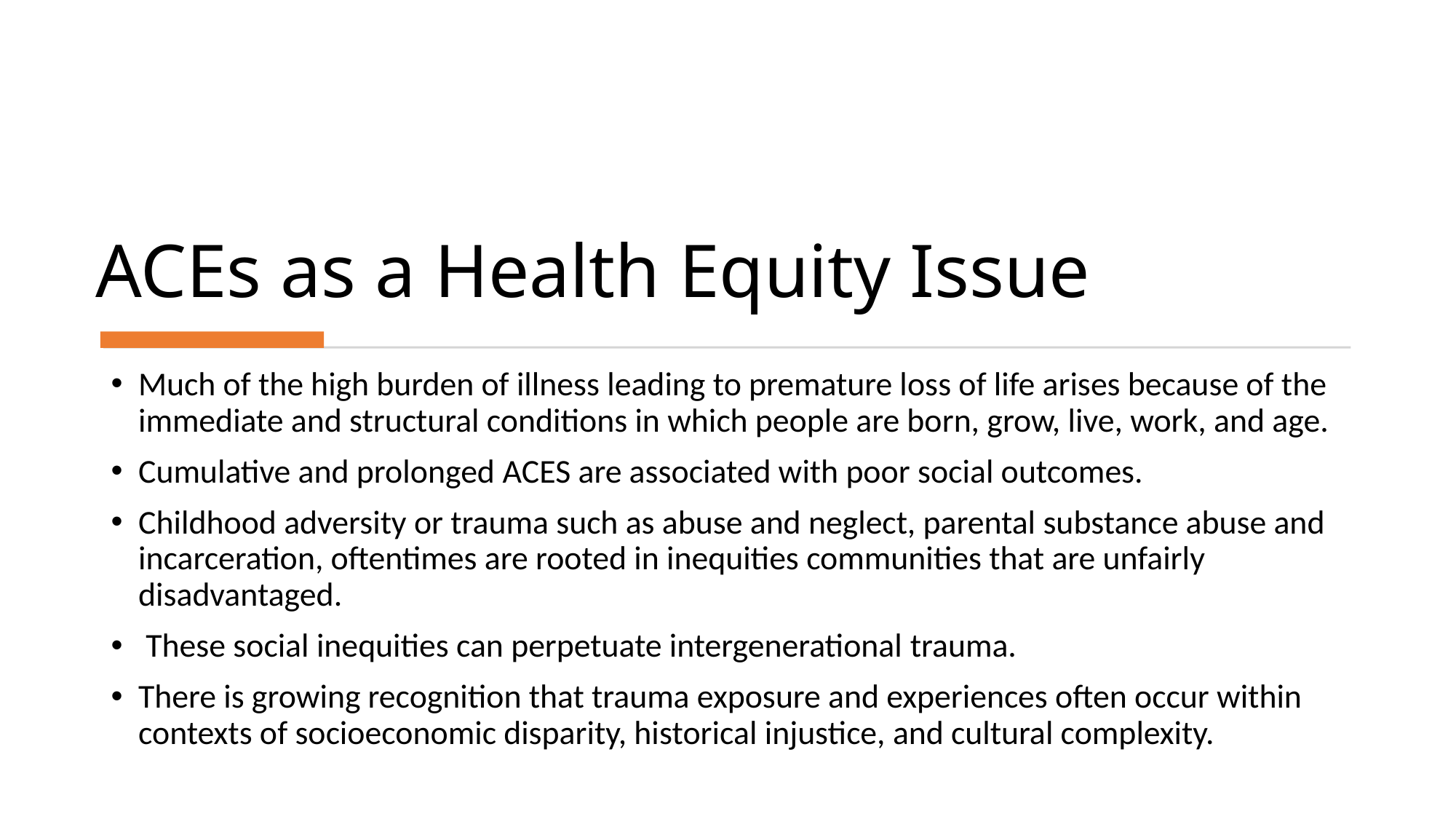

# ACEs as a Health Equity Issue
Much of the high burden of illness leading to premature loss of life arises because of the immediate and structural conditions in which people are born, grow, live, work, and age.
Cumulative and prolonged ACES are associated with poor social outcomes.
Childhood adversity or trauma such as abuse and neglect, parental substance abuse and incarceration, oftentimes are rooted in inequities communities that are unfairly disadvantaged.
 These social inequities can perpetuate intergenerational trauma.
There is growing recognition that trauma exposure and experiences often occur within contexts of socioeconomic disparity, historical injustice, and cultural complexity.

## Slide 22
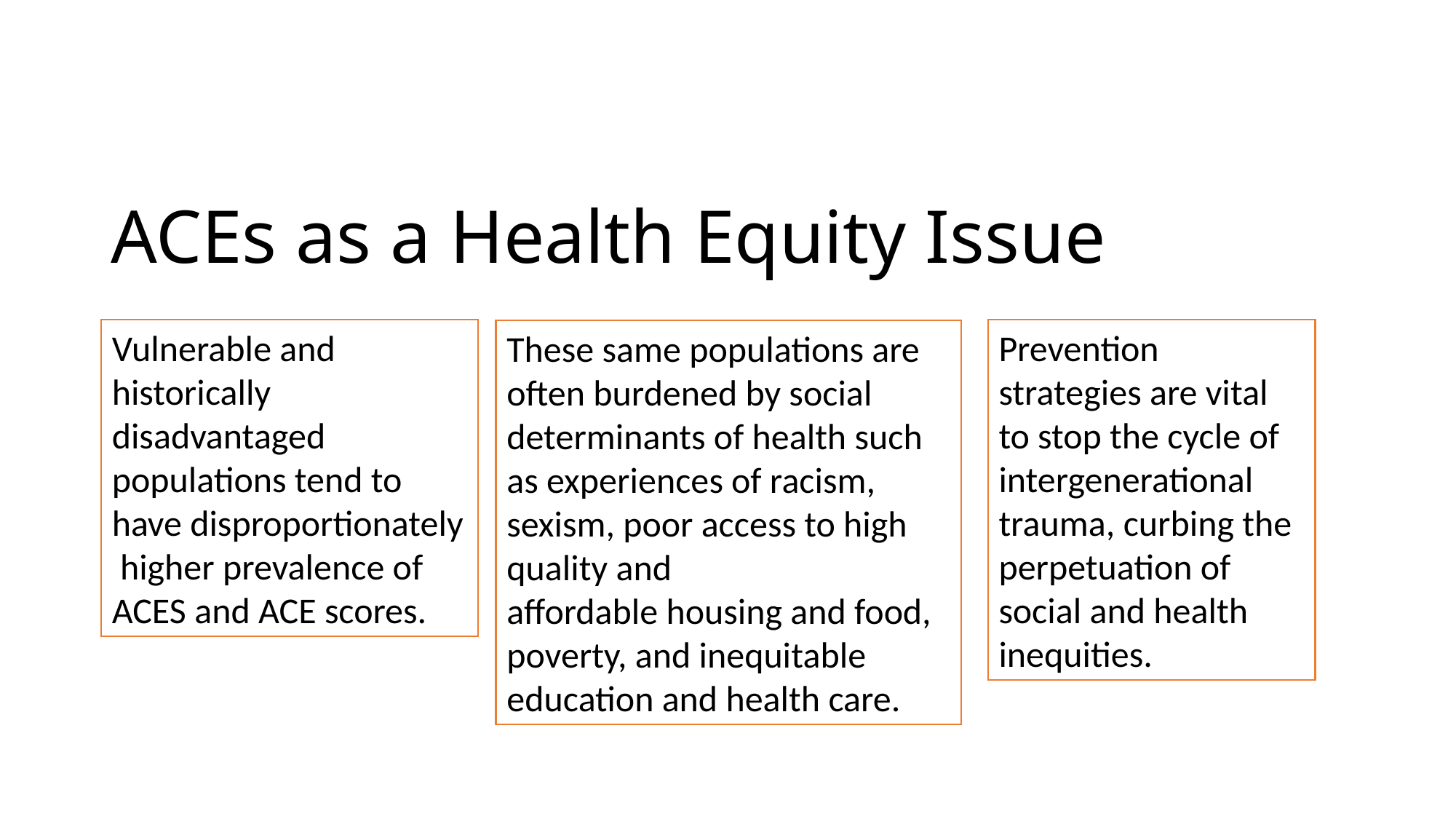

# ACEs as a Health Equity Issue
Prevention strategies are vital to stop the cycle of intergenerational trauma, curbing the perpetuation of social and health inequities.
Vulnerable and historically disadvantaged populations tend to have disproportionately higher prevalence of ACES and ACE scores.
These same populations are often burdened by social determinants of health such as experiences of racism, sexism, poor access to high quality and affordable housing and food, poverty, and inequitable education and health care.

## Slide 23
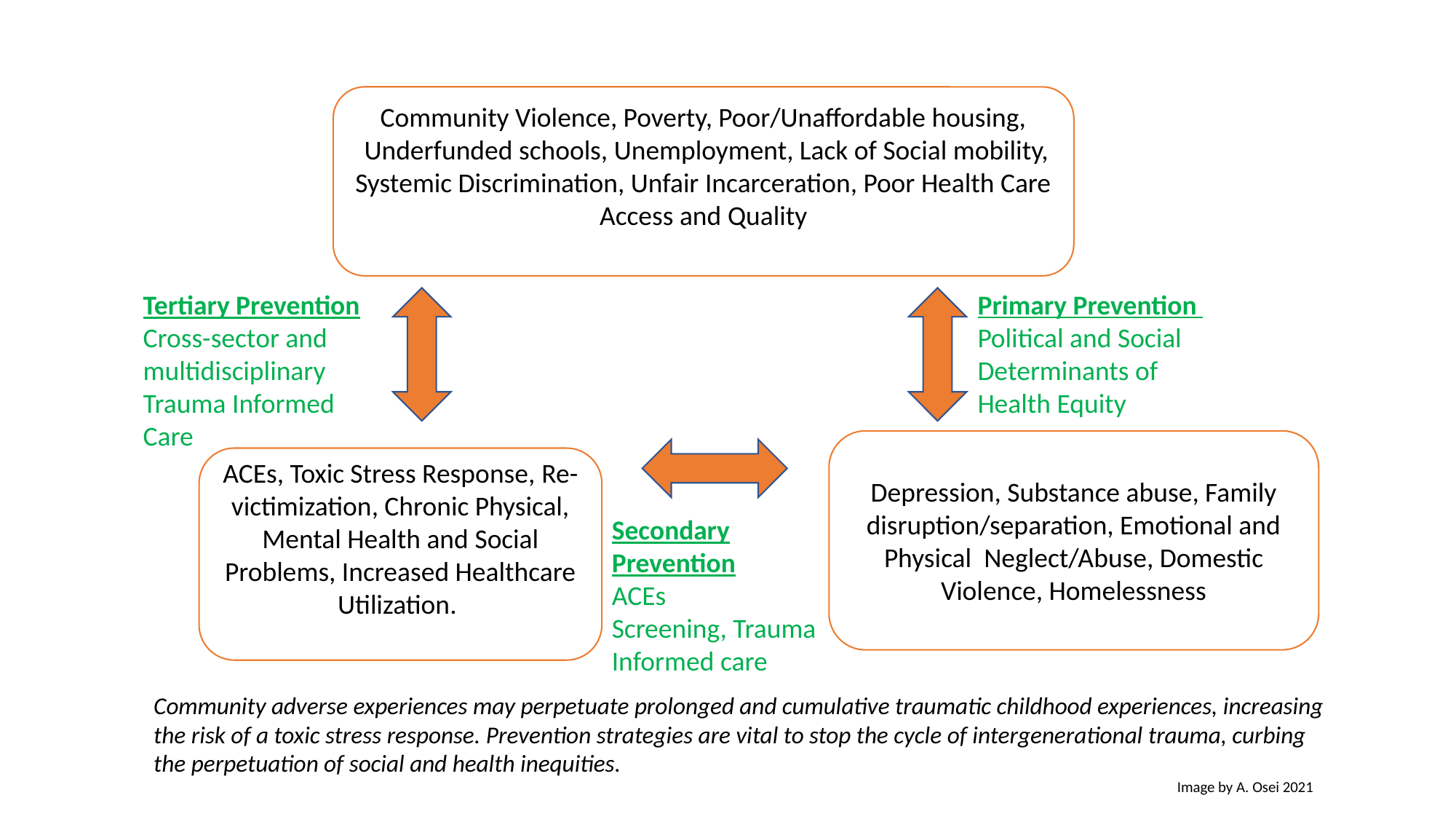

Community Violence, Poverty, Poor/Unaffordable housing,  Underfunded schools, Unemployment, Lack of Social mobility, Systemic Discrimination, Unfair Incarceration, Poor Health Care Access and Quality
Primary Prevention
Political and Social Determinants of Health Equity
Tertiary Prevention
Cross-sector and multidisciplinary Trauma Informed Care
Depression, Substance abuse, Family disruption/separation, Emotional and Physical  Neglect/Abuse, Domestic Violence, Homelessness
ACEs, Toxic Stress Response, Re-victimization, Chronic Physical, Mental Health and Social Problems, Increased Healthcare Utilization.
Secondary
Prevention
ACEs Screening, Trauma Informed care
Community adverse experiences may perpetuate prolonged and cumulative traumatic childhood experiences, increasing the risk of a toxic stress response. Prevention strategies are vital to stop the cycle of intergenerational trauma, curbing the perpetuation of social and health inequities.
Image by A. Osei 2021

## Slide 24
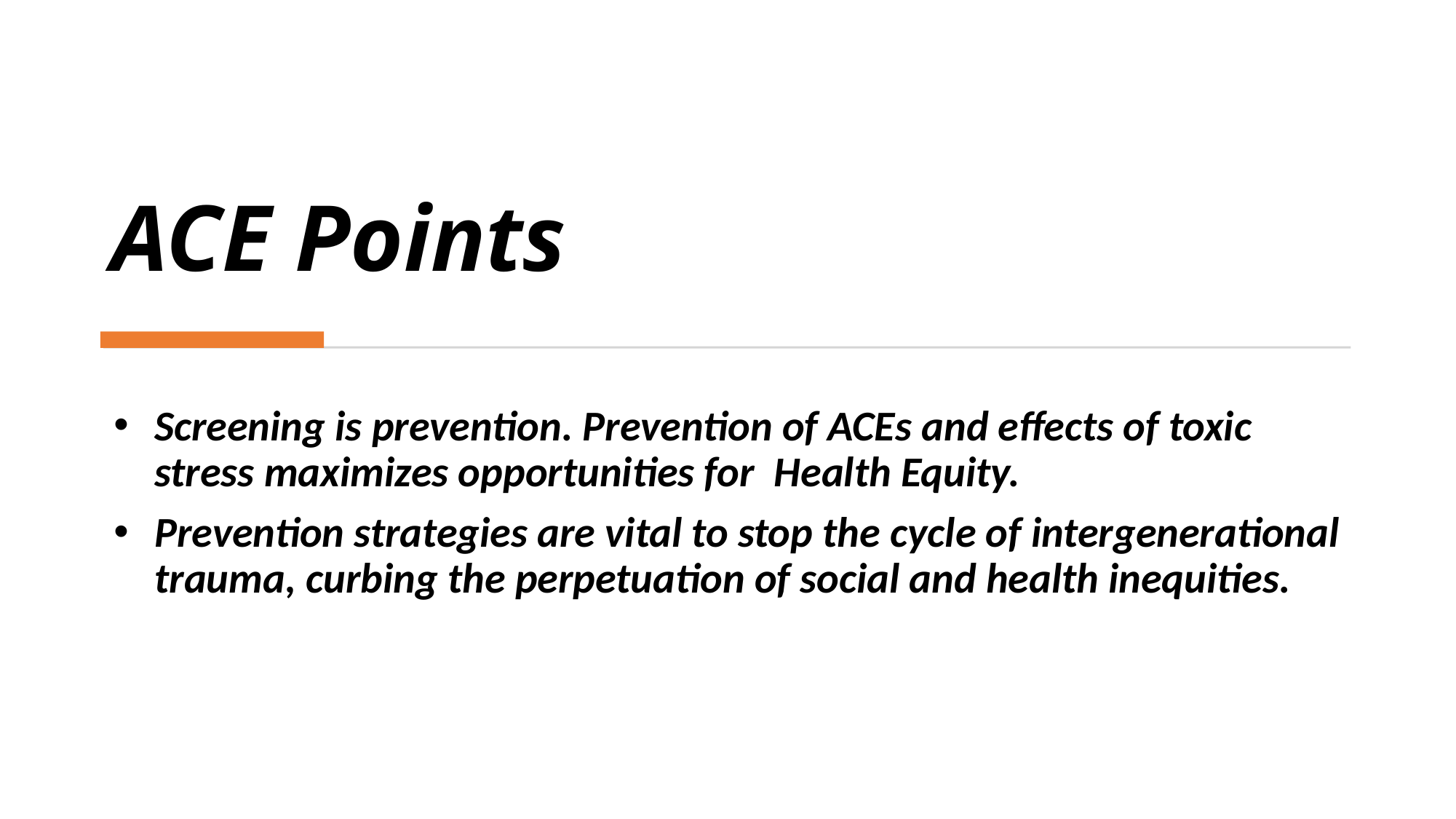

# ACE Points
Screening is prevention. Prevention of ACEs and effects of toxic stress maximizes opportunities for  Health Equity.
Prevention strategies are vital to stop the cycle of intergenerational trauma, curbing the perpetuation of social and health inequities.

## Slide 25
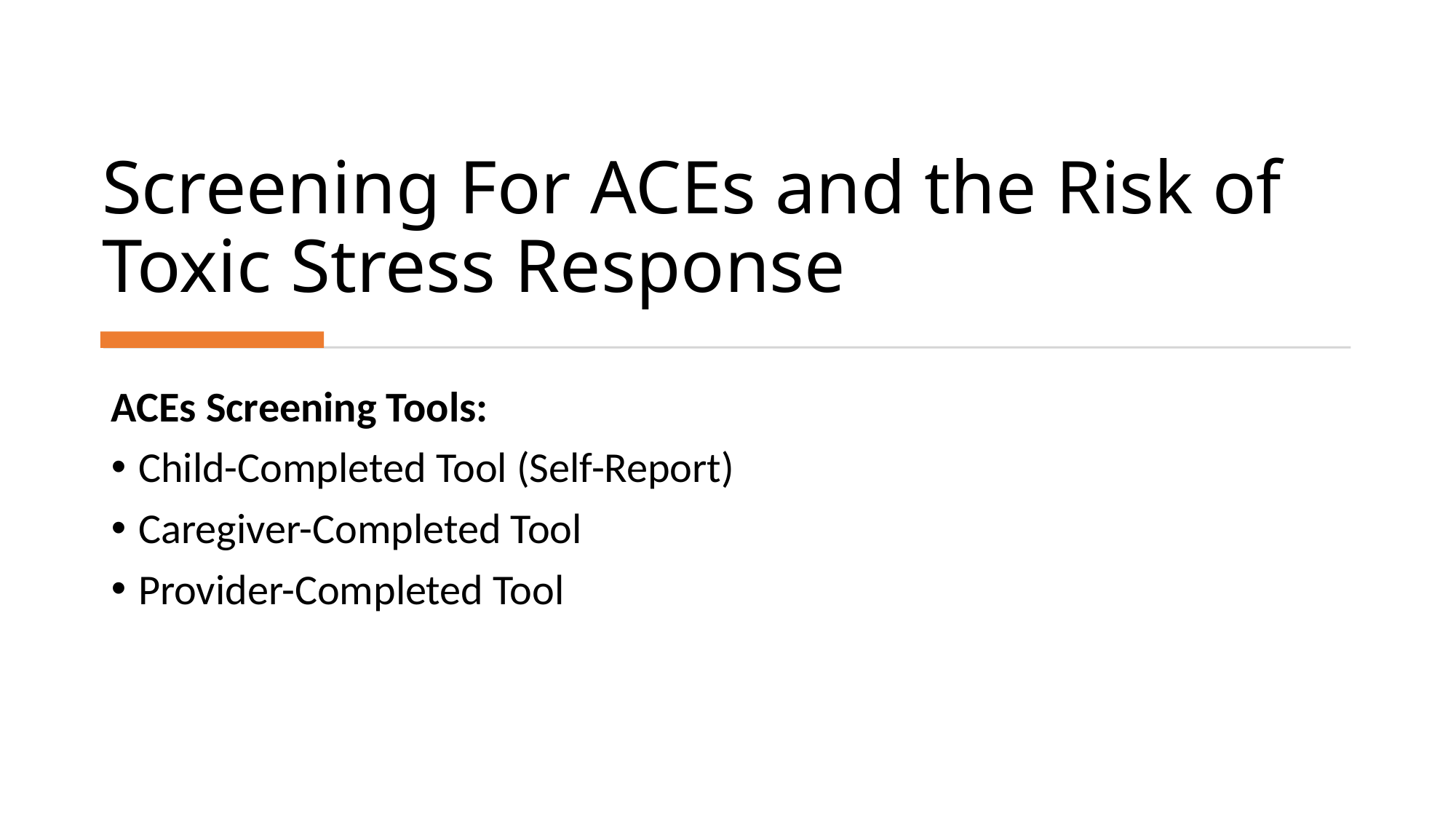

# Screening For ACEs and the Risk of Toxic Stress Response
ACEs Screening Tools:
Child-Completed Tool (Self-Report)
Caregiver-Completed Tool
Provider-Completed Tool

## Slide 26
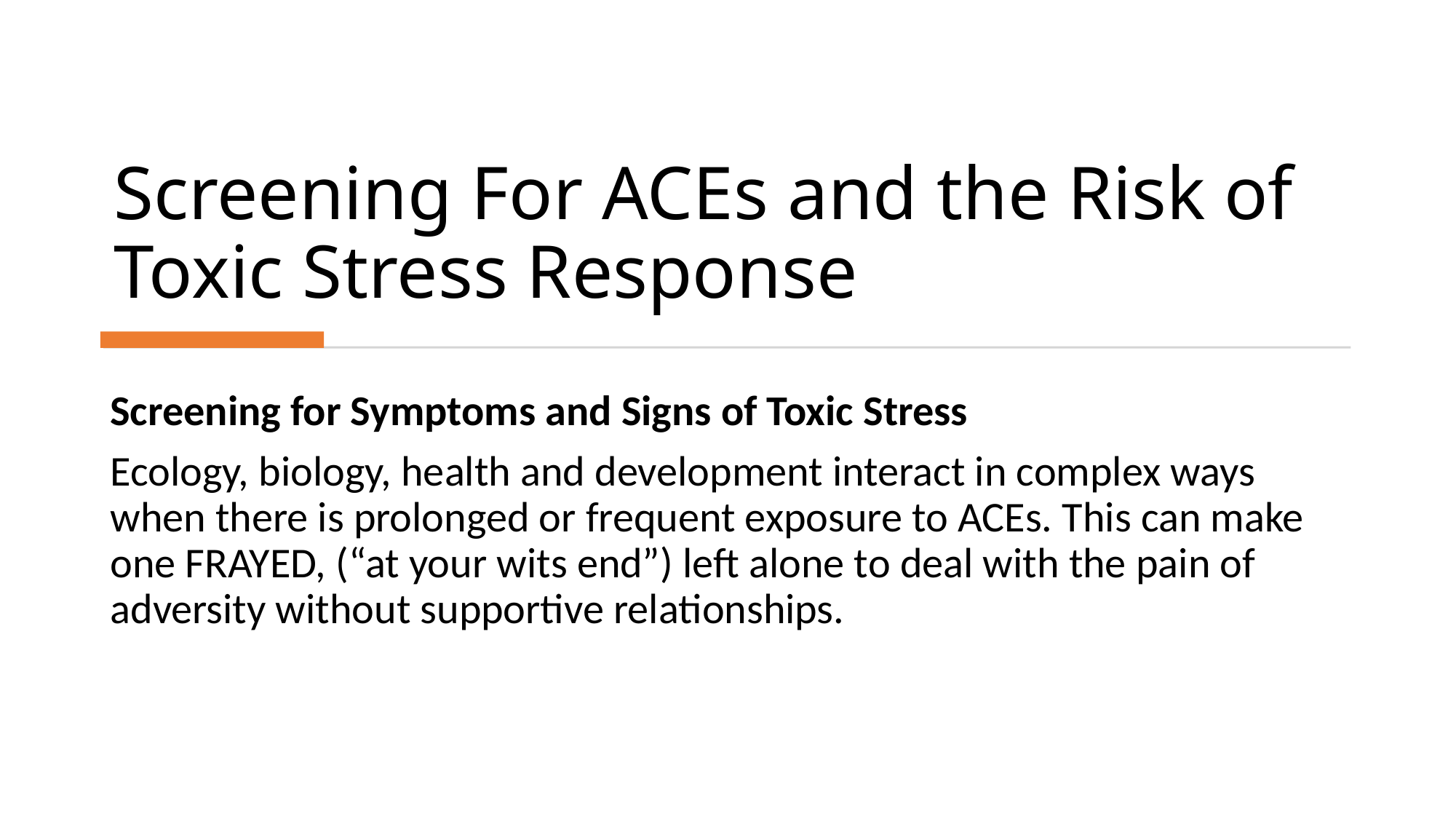

Screening For ACEs and the Risk of Toxic Stress Response
Screening for Symptoms and Signs of Toxic Stress
Ecology, biology, health and development interact in complex ways when there is prolonged or frequent exposure to ACEs. This can make one FRAYED, (“at your wits end”) left alone to deal with the pain of adversity without supportive relationships.

## Slide 27
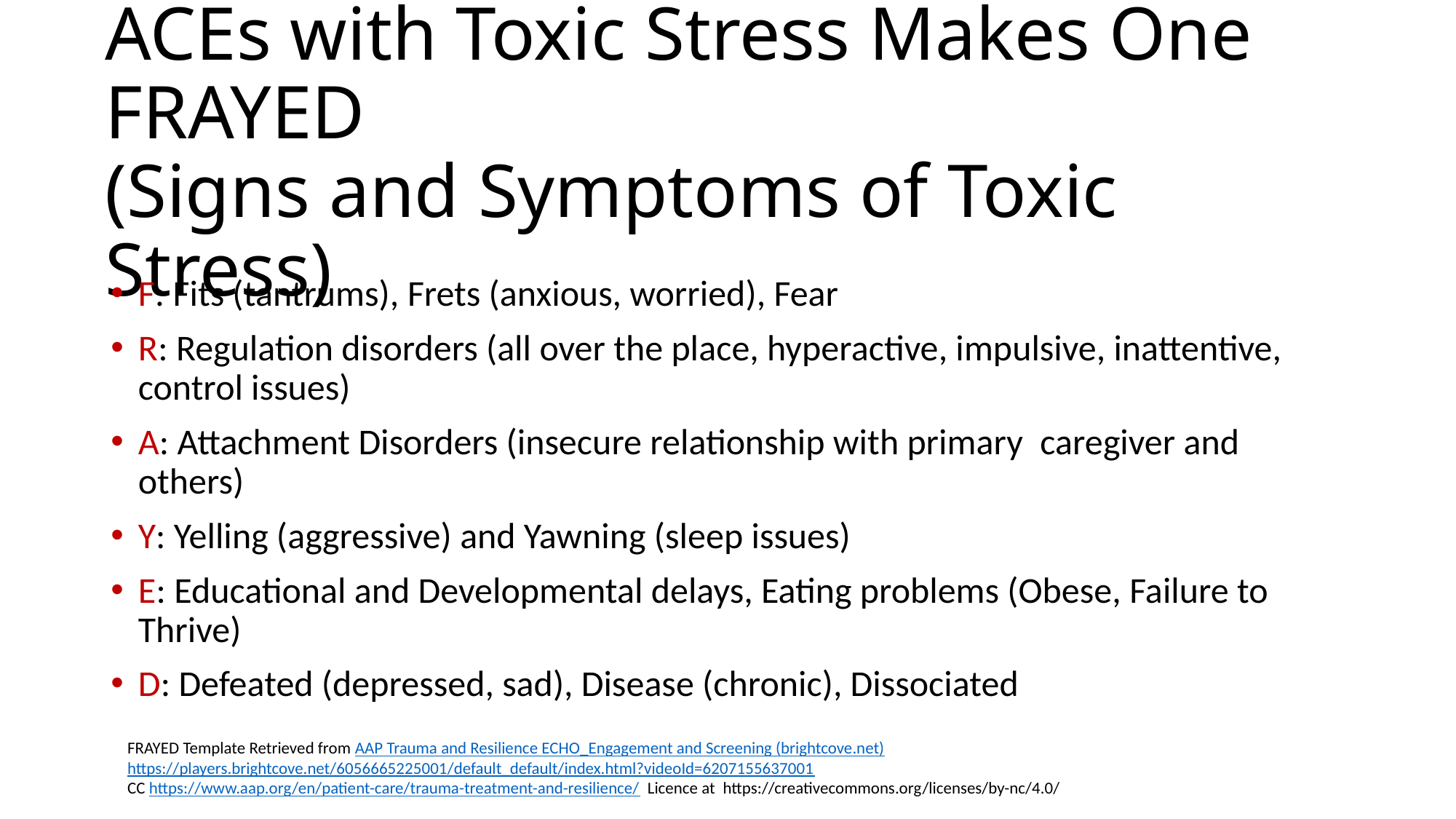

# ACEs with Toxic Stress Makes One FRAYED (Signs and Symptoms of Toxic Stress)
F: Fits (tantrums), Frets (anxious, worried), Fear
R: Regulation disorders (all over the place, hyperactive, impulsive, inattentive, control issues)
A: Attachment Disorders (insecure relationship with primary  caregiver and others)
Y: Yelling (aggressive) and Yawning (sleep issues)
E: Educational and Developmental delays, Eating problems (Obese, Failure to Thrive)
D: Defeated (depressed, sad), Disease (chronic), Dissociated
FRAYED Template Retrieved from AAP Trauma and Resilience ECHO_Engagement and Screening (brightcove.net)   https://players.brightcove.net/6056665225001/default_default/index.html?videoId=6207155637001
CC https://www.aap.org/en/patient-care/trauma-treatment-and-resilience/  Licence at  https://creativecommons.org/licenses/by-nc/4.0/

## Slide 28
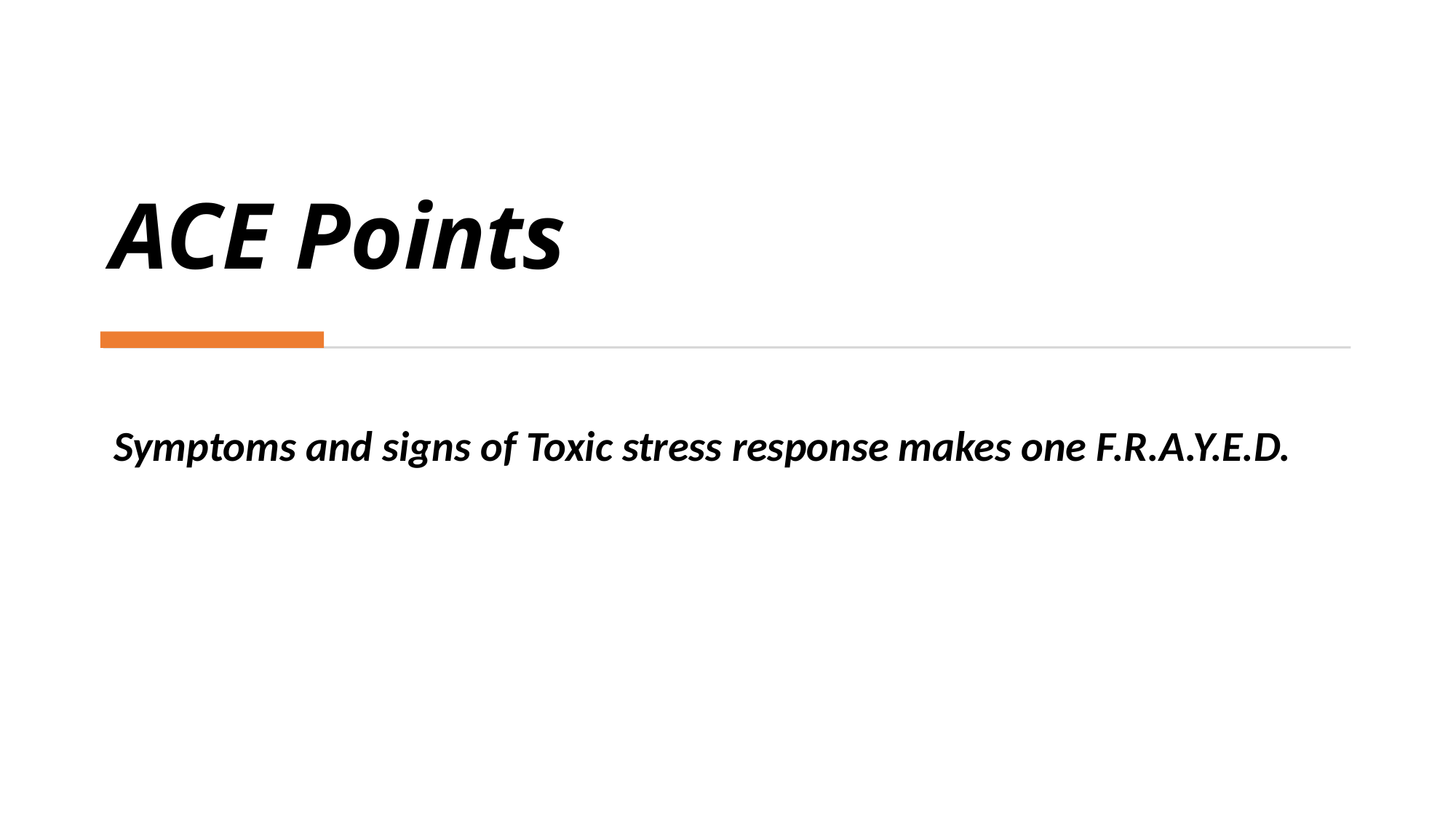

# ACE Points
Symptoms and signs of Toxic stress response makes one F.R.A.Y.E.D.

## Slide 29
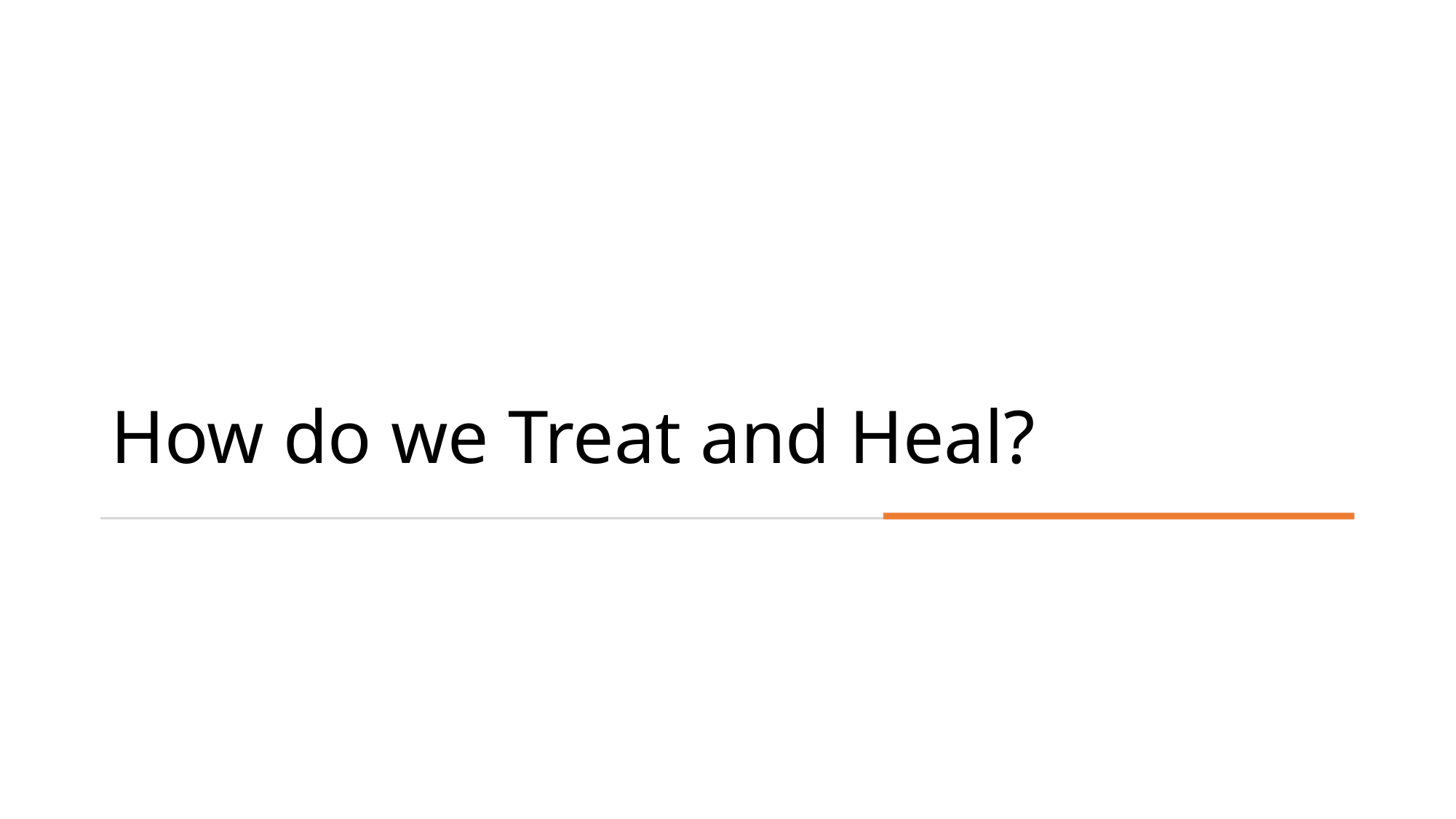

# How do we Treat and Heal?

## Slide 30
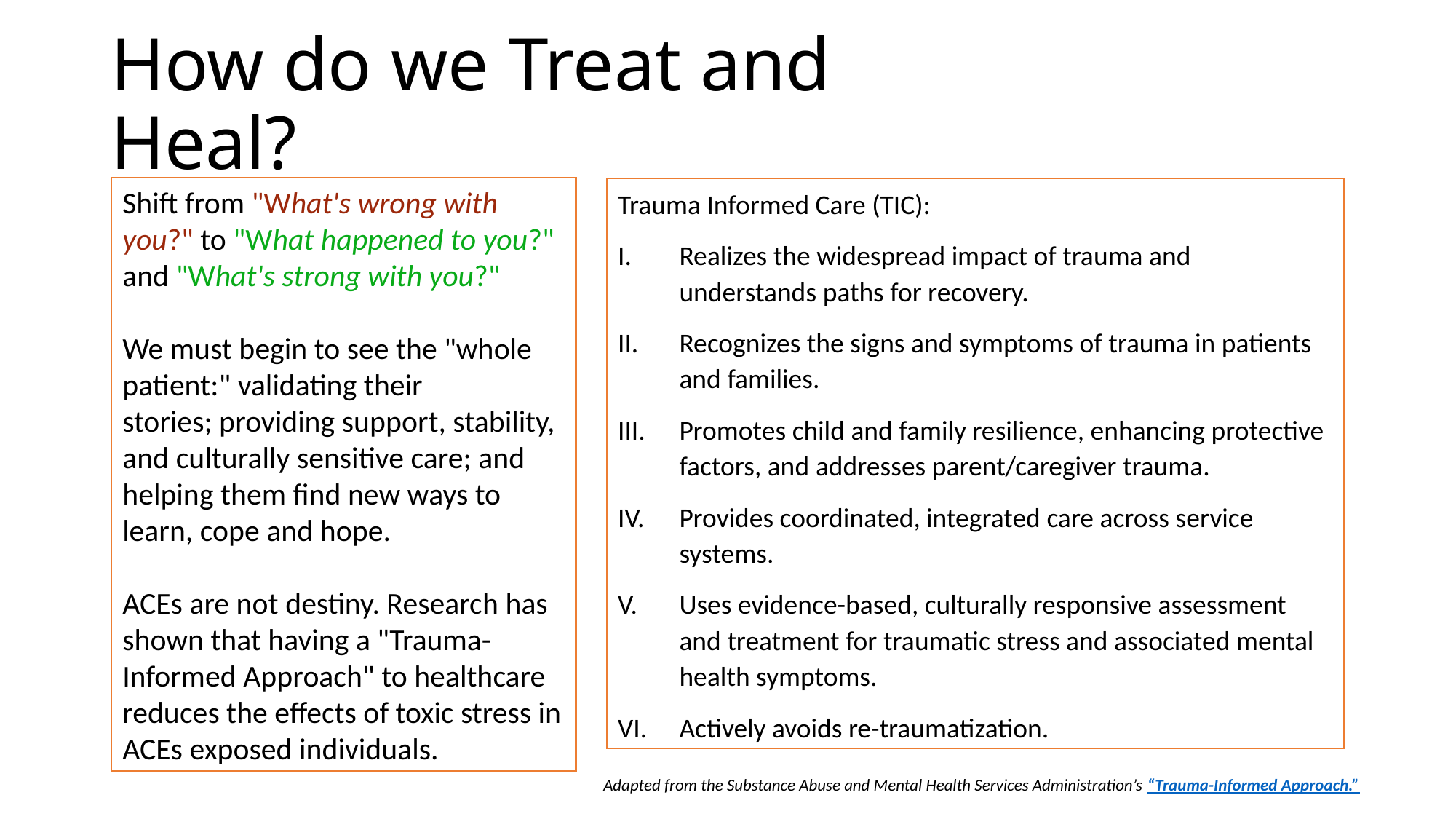

How do we Treat and Heal?
Shift from "What's wrong with you?" to "What happened to you?" and "What's strong with you?"
We must begin to see the "whole patient:" validating their stories; providing support, stability, and culturally sensitive care; and
helping them find new ways to learn, cope and hope.
ACEs are not destiny. Research has shown that having a "Trauma-Informed Approach" to healthcare reduces the effects of toxic stress in ACEs exposed individuals.
Trauma Informed Care (TIC):
Realizes the widespread impact of trauma and understands paths for recovery.
Recognizes the signs and symptoms of trauma in patients and families.
Promotes child and family resilience, enhancing protective factors, and addresses parent/caregiver trauma.
Provides coordinated, integrated care across service systems.
Uses evidence-based, culturally responsive assessment and treatment for traumatic stress and associated mental health symptoms.
Actively avoids re-traumatization.
Adapted from the Substance Abuse and Mental Health Services Administration’s “Trauma-Informed Approach.”

## Slide 31
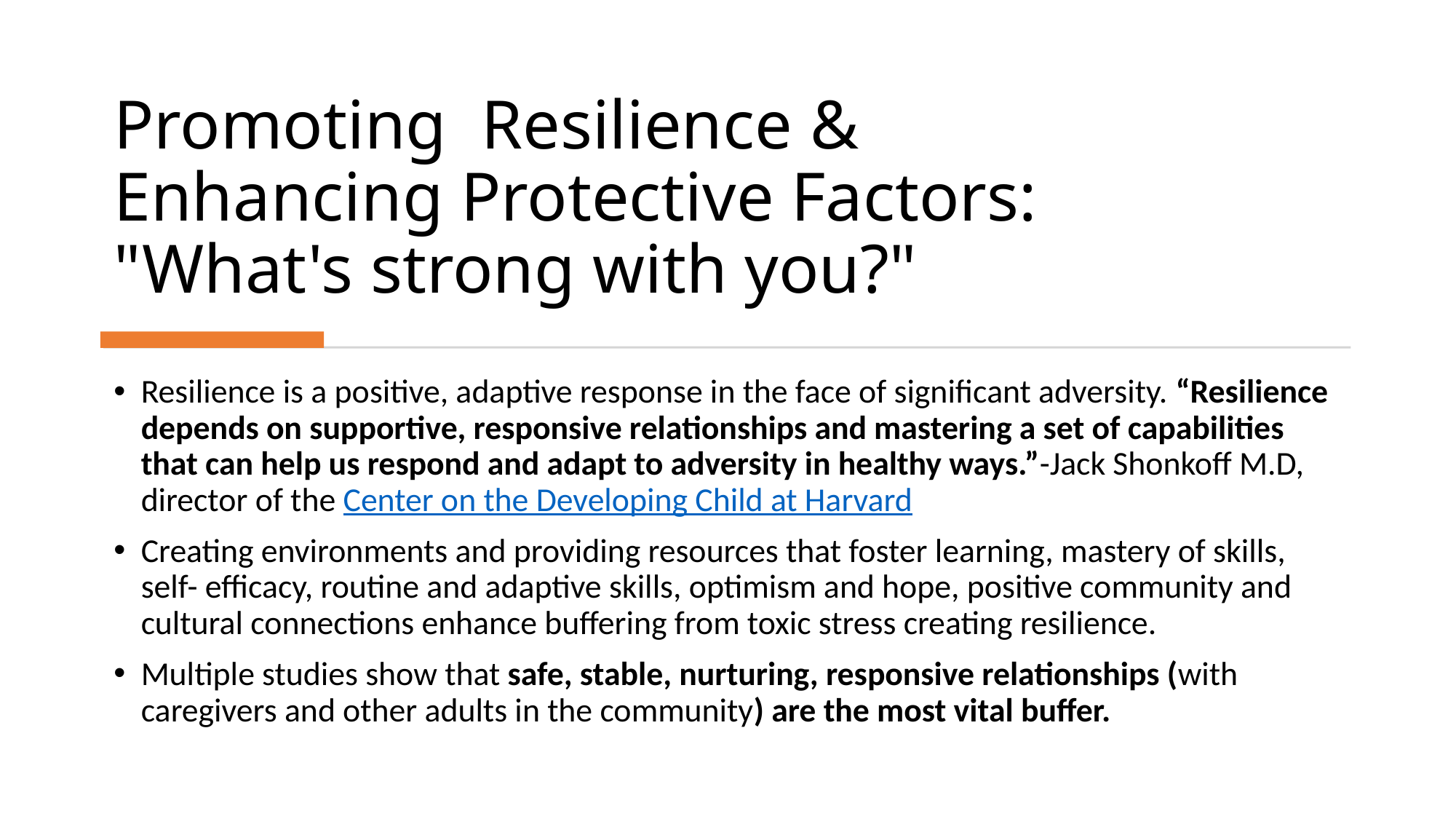

# Promoting  Resilience & Enhancing Protective Factors: "What's strong with you?"
Resilience is a positive, adaptive response in the face of significant adversity. “Resilience depends on supportive, responsive relationships and mastering a set of capabilities that can help us respond and adapt to adversity in healthy ways.”-Jack Shonkoff M.D, director of the Center on the Developing Child at Harvard
Creating environments and providing resources that foster learning, mastery of skills, self- efficacy, routine and adaptive skills, optimism and hope, positive community and cultural connections enhance buffering from toxic stress creating resilience.
Multiple studies show that safe, stable, nurturing, responsive relationships (with caregivers and other adults in the community) are the most vital buffer.

## Slide 32
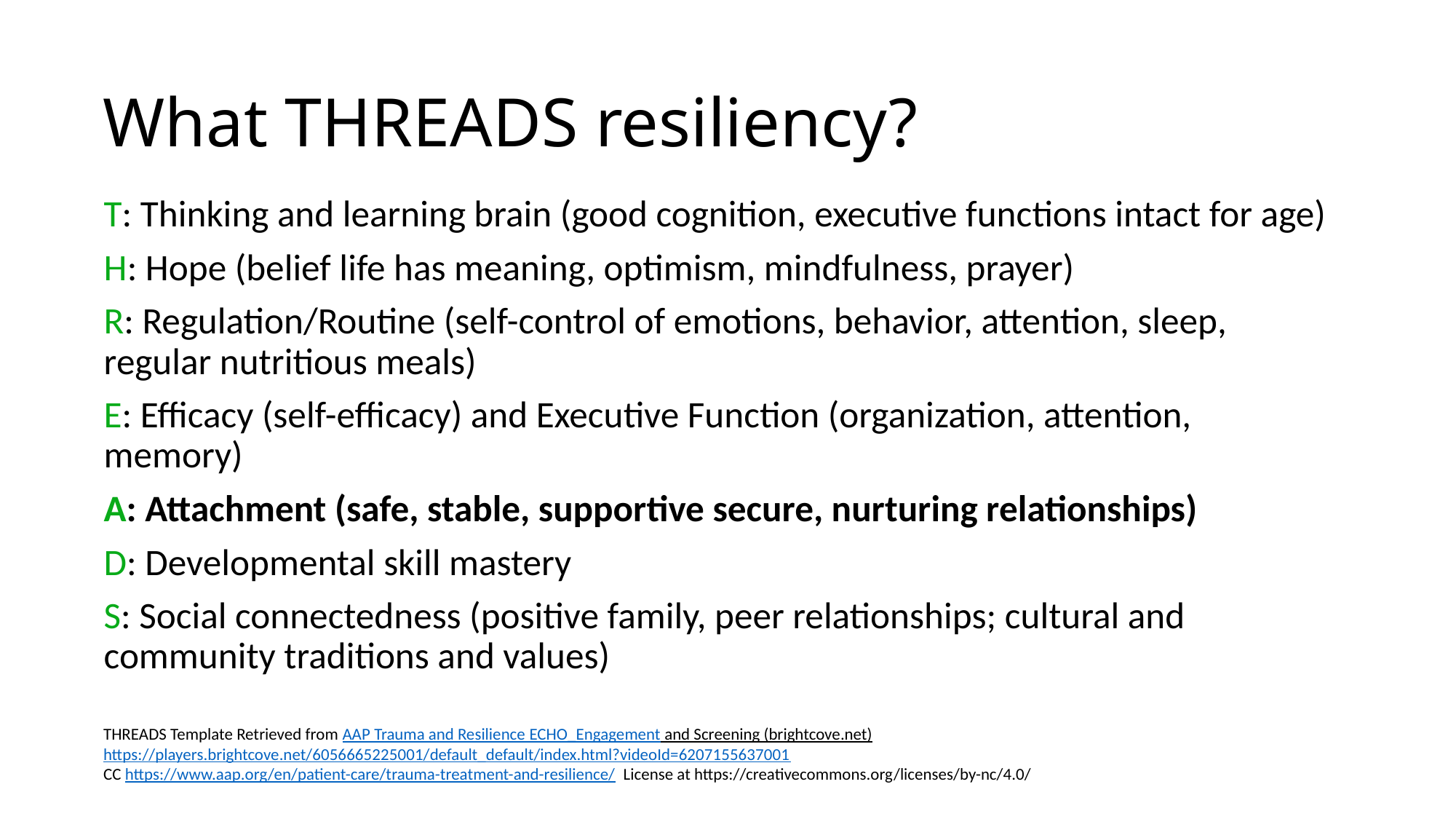

# What THREADS resiliency?
T: Thinking and learning brain (good cognition, executive functions intact for age)
H: Hope (belief life has meaning, optimism, mindfulness, prayer)
R: Regulation/Routine (self-control of emotions, behavior, attention, sleep, regular nutritious meals)
E: Efficacy (self-efficacy) and Executive Function (organization, attention, memory)
A: Attachment (safe, stable, supportive secure, nurturing relationships)
D: Developmental skill mastery
S: Social connectedness (positive family, peer relationships; cultural and community traditions and values)
THREADS Template Retrieved from AAP Trauma and Resilience ECHO_Engagement and Screening (brightcove.net)   https://players.brightcove.net/6056665225001/default_default/index.html?videoId=6207155637001
CC https://www.aap.org/en/patient-care/trauma-treatment-and-resilience/  License at https://creativecommons.org/licenses/by-nc/4.0/

## Slide 33
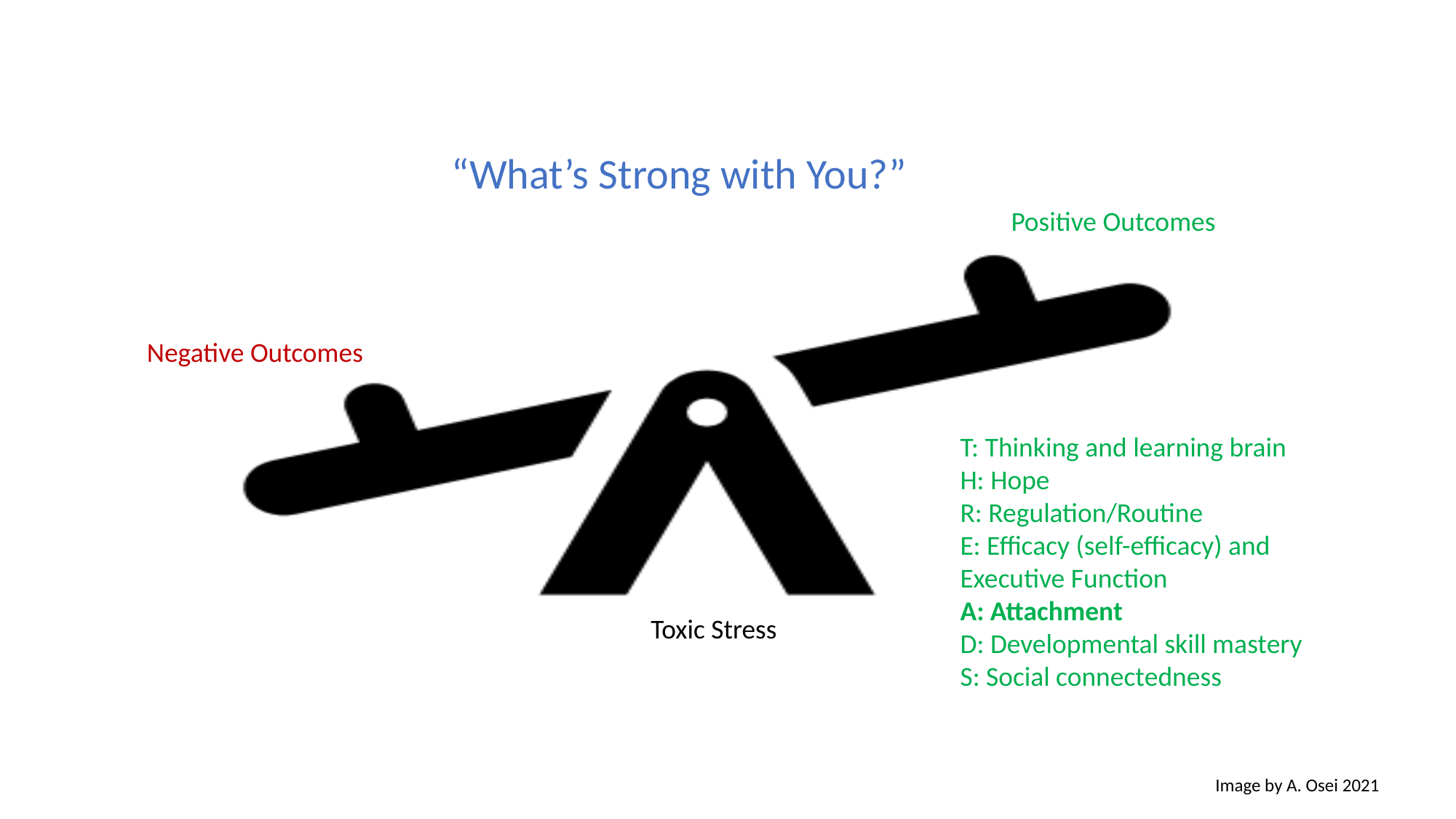

“What’s Strong with You?”
Positive Outcomes
Negative Outcomes
T: Thinking and learning brain
H: Hope
R: Regulation/Routine
E: Efficacy (self-efficacy) and Executive Function
A: Attachment
D: Developmental skill mastery
S: Social connectedness
 Toxic Stress
Image by A. Osei 2021

## Slide 34
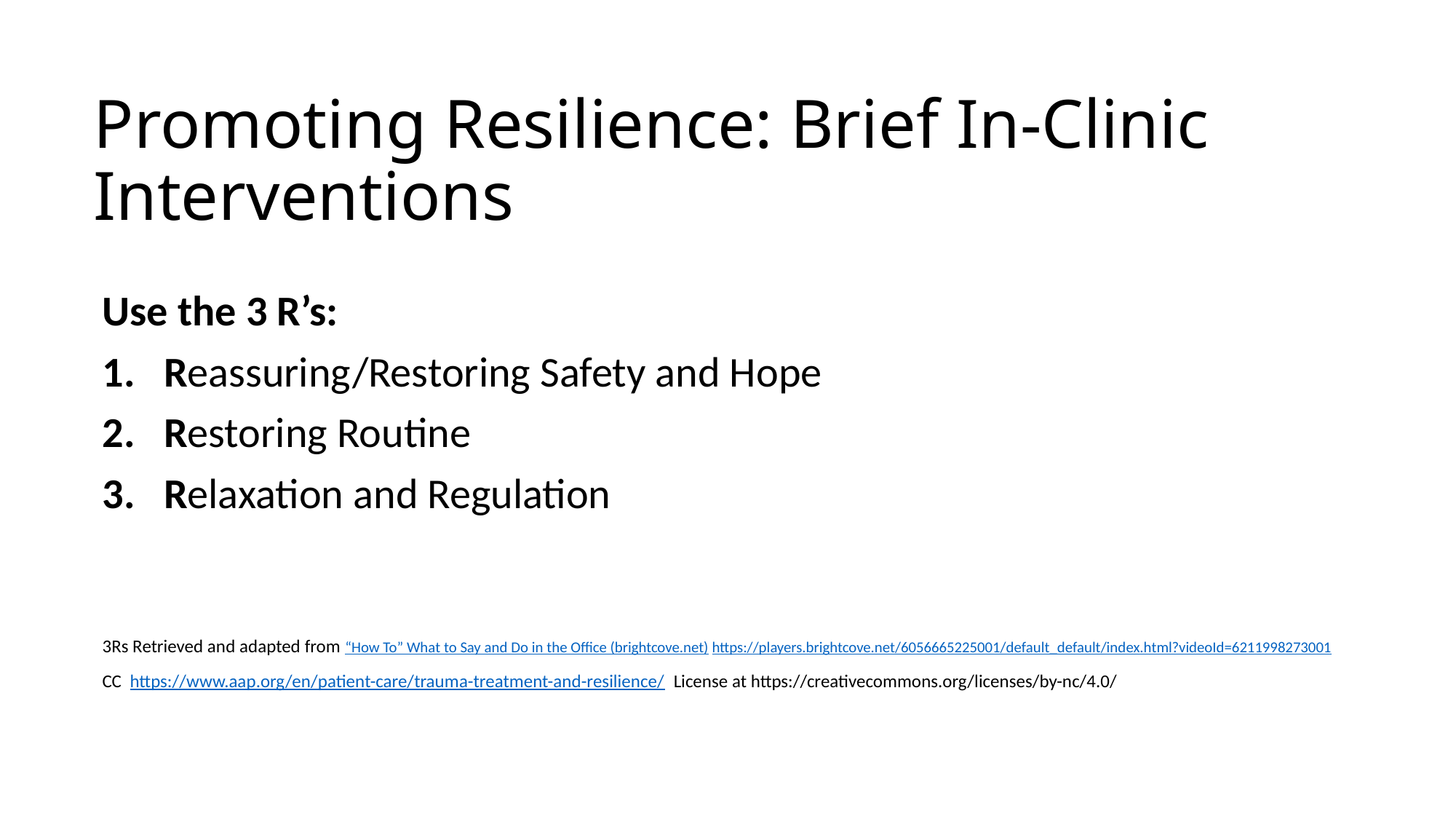

# Promoting Resilience: Brief In-Clinic Interventions
Use the 3 R’s:
Reassuring/Restoring Safety and Hope
Restoring Routine
Relaxation and Regulation
3Rs Retrieved and adapted from “How To” What to Say and Do in the Office (brightcove.net) https://players.brightcove.net/6056665225001/default_default/index.html?videoId=6211998273001
CC  https://www.aap.org/en/patient-care/trauma-treatment-and-resilience/  License at https://creativecommons.org/licenses/by-nc/4.0/

## Slide 35
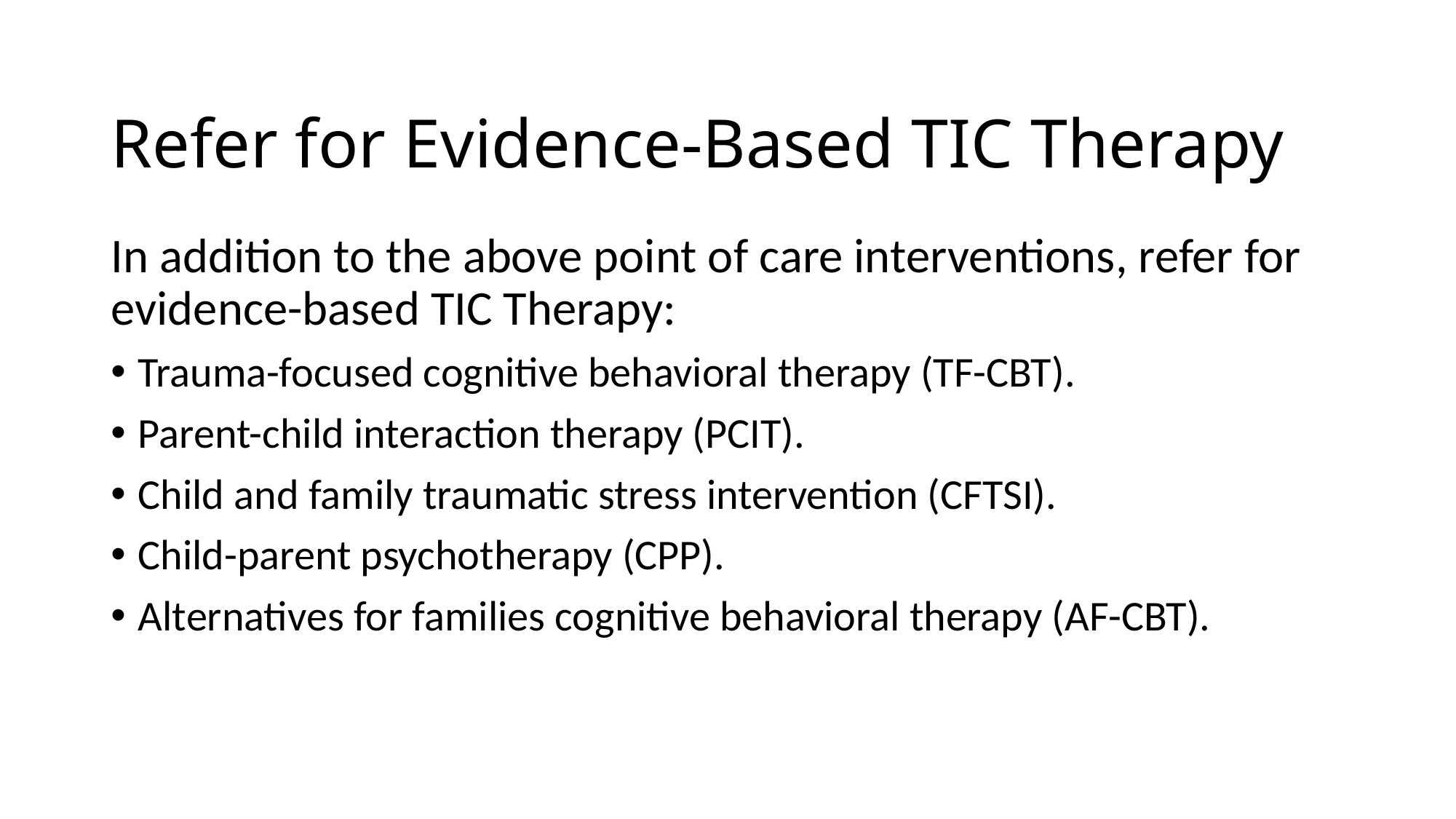

# Refer for Evidence-Based TIC Therapy
In addition to the above point of care interventions, refer for evidence-based TIC Therapy:
Trauma-focused cognitive behavioral therapy (TF-CBT).
Parent-child interaction therapy (PCIT).
Child and family traumatic stress intervention (CFTSI).
Child-parent psychotherapy (CPP).
Alternatives for families cognitive behavioral therapy (AF-CBT).

## Slide 36
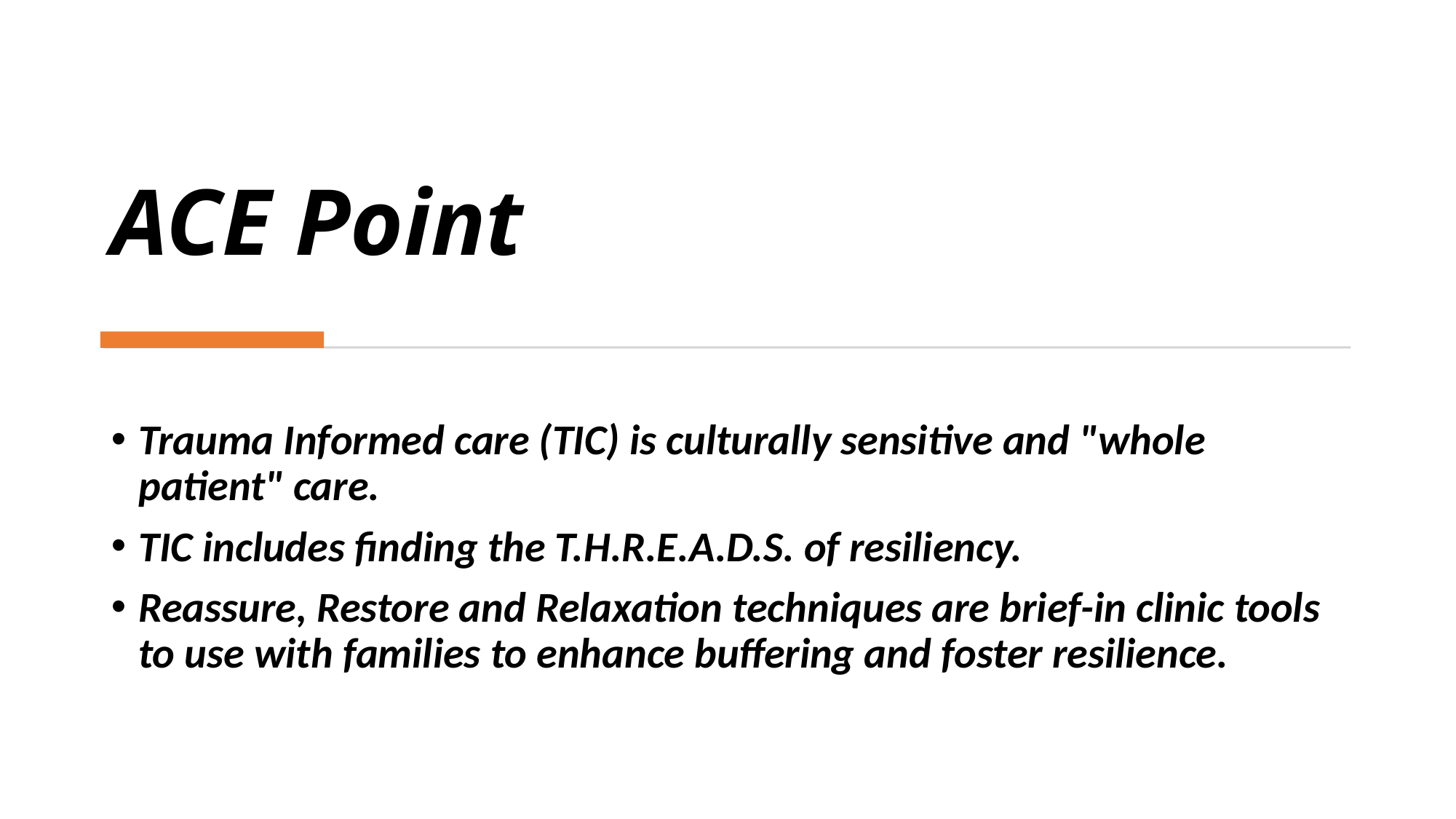

# ACE Point
Trauma Informed care (TIC) is culturally sensitive and "whole patient" care.
TIC includes finding the T.H.R.E.A.D.S. of resiliency.
Reassure, Restore and Relaxation techniques are brief-in clinic tools to use with families to enhance buffering and foster resilience.

## Slide 37
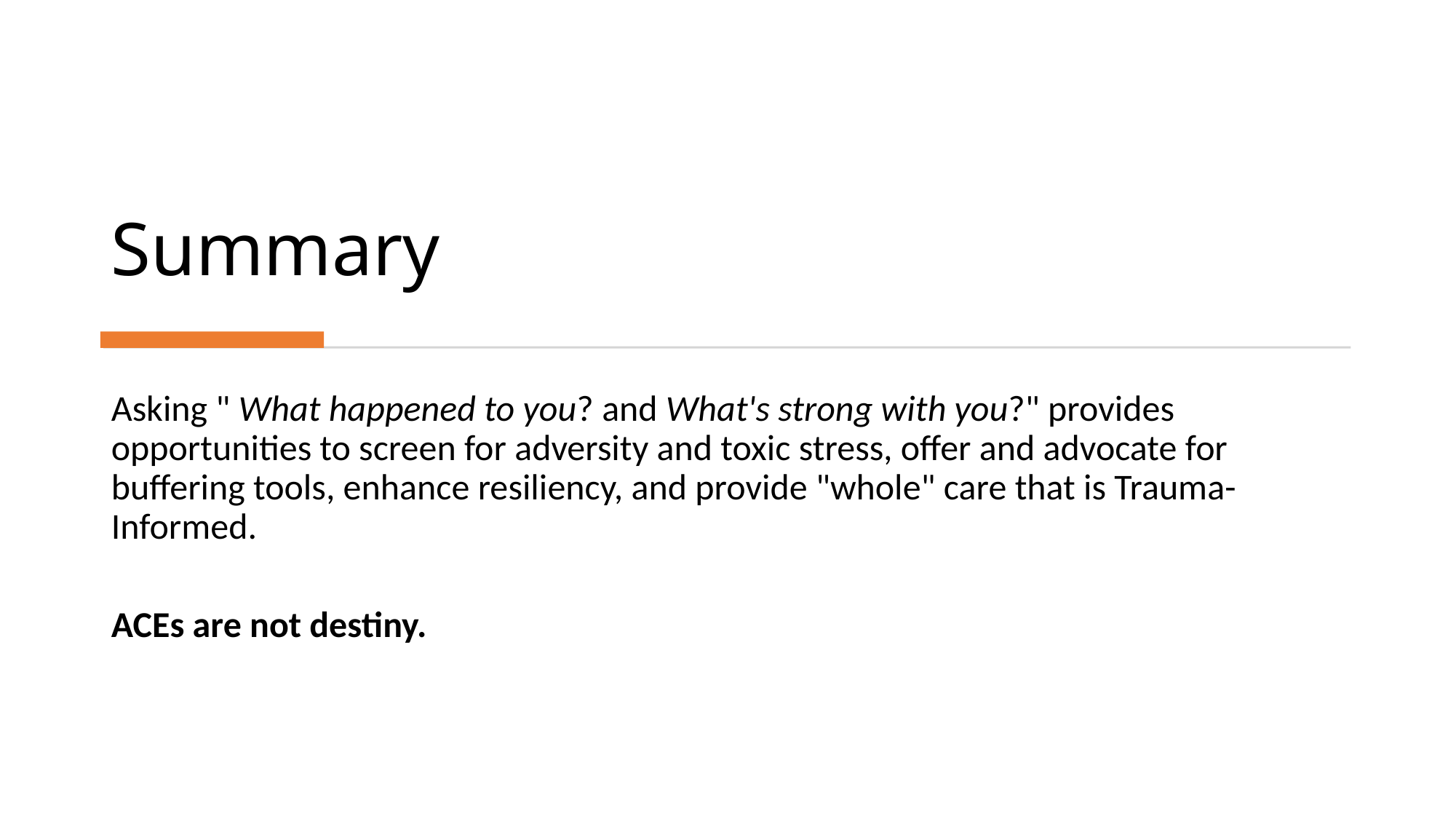

# Summary
Asking " What happened to you? and What's strong with you?" provides opportunities to screen for adversity and toxic stress, offer and advocate for buffering tools, enhance resiliency, and provide "whole" care that is Trauma-Informed.
ACEs are not destiny.

## Slide 38
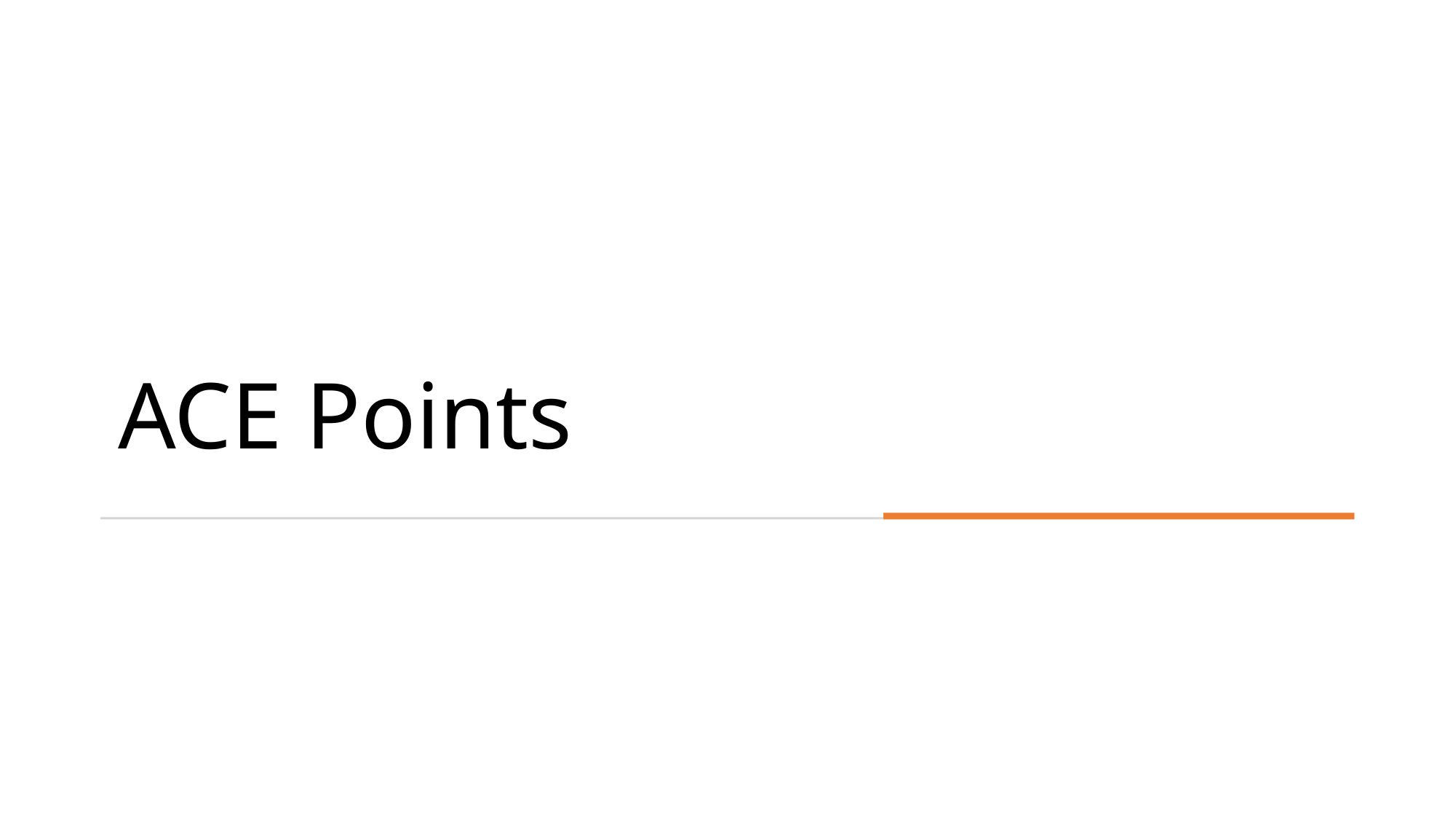

# ACE Points

## Slide 39
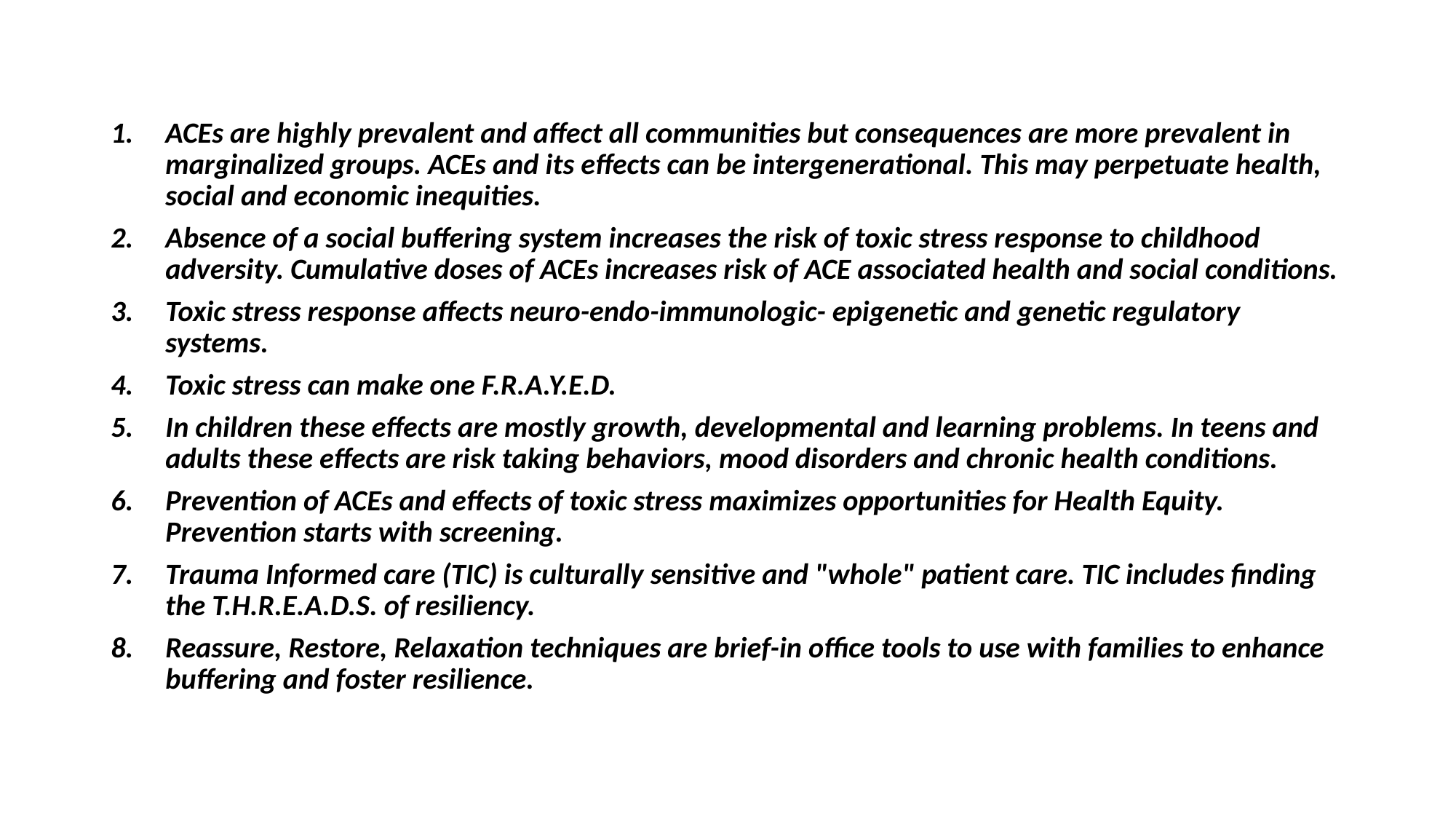

ACEs are highly prevalent and affect all communities but consequences are more prevalent in marginalized groups. ACEs and its effects can be intergenerational. This may perpetuate health, social and economic inequities.
Absence of a social buffering system increases the risk of toxic stress response to childhood adversity. Cumulative doses of ACEs increases risk of ACE associated health and social conditions.
Toxic stress response affects neuro-endo-immunologic- epigenetic and genetic regulatory systems.
Toxic stress can make one F.R.A.Y.E.D.
In children these effects are mostly growth, developmental and learning problems. In teens and adults these effects are risk taking behaviors, mood disorders and chronic health conditions.
Prevention of ACEs and effects of toxic stress maximizes opportunities for Health Equity. Prevention starts with screening.
Trauma Informed care (TIC) is culturally sensitive and "whole" patient care. TIC includes finding the T.H.R.E.A.D.S. of resiliency.
Reassure, Restore, Relaxation techniques are brief-in office tools to use with families to enhance buffering and foster resilience.

## Slide 40
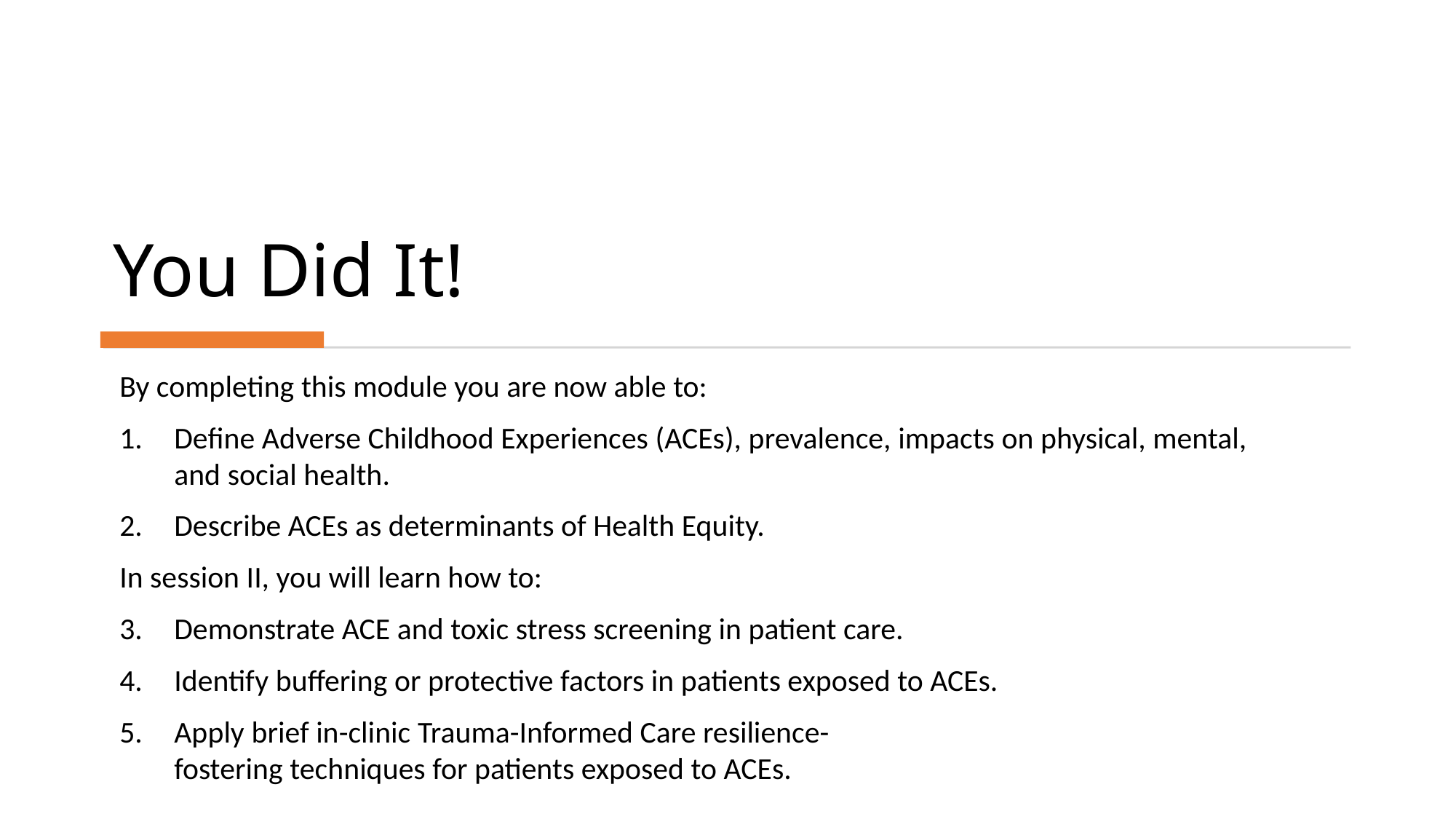

# You Did It!
By completing this module you are now able to:
Define Adverse Childhood Experiences (ACEs), prevalence, impacts on physical, mental, and social health.
Describe ACEs as determinants of Health Equity.
In session II, you will learn how to:
Demonstrate ACE and toxic stress screening in patient care.
Identify buffering or protective factors in patients exposed to ACEs.
Apply brief in-clinic Trauma-Informed Care resilience-fostering techniques for patients exposed to ACEs.
